# Optical Phased Arrays on Photonic Integrated Circuit Platforms: Challenges, Opportunities, and Future Applications

**DOI:** 10.1002/nap2.70182

**Published:** 2026-07-18

**Authors:** Jingwei Li, Yuyuan Han, Aoxiang Chen, Pengpeng Li, Xinglin Zhu, Guanbao Zhao, Luohan Peng, Huaibin Zheng

**Affiliations:** ^1^ Micius Laboratory Henan Academy of Sciences Zhengzhou Henan China; ^2^ Electronic Materials Research Laboratory Key Laboratory of the Ministry of Education and International Center for Dielectric Research School of Electronic Science and Engineering Xi'an Jiaotong University Xi'an Shaanxi China; ^3^ College of Materials Science and Engineering Key Laboratory of Interface Science and Engineering in Advanced Materials Ministry of Education Taiyuan University of Technology Taiyuan Shanxi China

**Keywords:** beam steering, optical phased array, photonic integrated circuits

## Abstract

On‐chip optical phased arrays (OPAs) have emerged as a core technology in photonic integrated circuits (PICs), enabling compact, solid‐state, and digitally reconfigurable beam steering with high scalability. By eliminating mechanical motion and leveraging semiconductor fabrication, OPAs provide a versatile platform for large‐aperture and high‐speed optical wavefront control in applications including LiDAR, free‐space optical communication, imaging, and holographic displays. Recent progress across silicon, silicon nitride, hybrid Si–${\text{Si}}_{3}N_{4}$, III–V, and lithium niobate‐based platforms has advanced OPAs from proof‐of‐concept demonstrations to highly integrated systems with sub‐milliradian resolution, wide fields of view, and megahertz‐to‐gigahertz update rates. This review presents a system‐level overview of on‐chip OPAs, covering fundamental beam‐steering principles, array theory, and key performance metrics, as well as the essential building blocks of laser sources, phase shifters, and grating antennas. Particular emphasis is placed on aperiodic array architectures, system‐level scalability challenges, and emerging electro‐optic platforms such as lithium niobate on insulator (LNOI), which are redefining the speed–power trade space. Finally, we discuss future directions toward programmable, large‐scale optical wavefront engines enabled by heterogeneous integration and intelligent control.

## Graphical abstract

Integrated optical phased arrays are emerging as scalable engines for solid‐state beam steering and programmable wavefront control. This Review maps the field from beam‐steering principles and core building blocks to material platforms, system‐level bottlenecks, and application frontiers, highlighting how heterogeneous integration, aperiodic architectures, and electro‐optic platforms are reshaping the speed‐power‐scalability trade space.

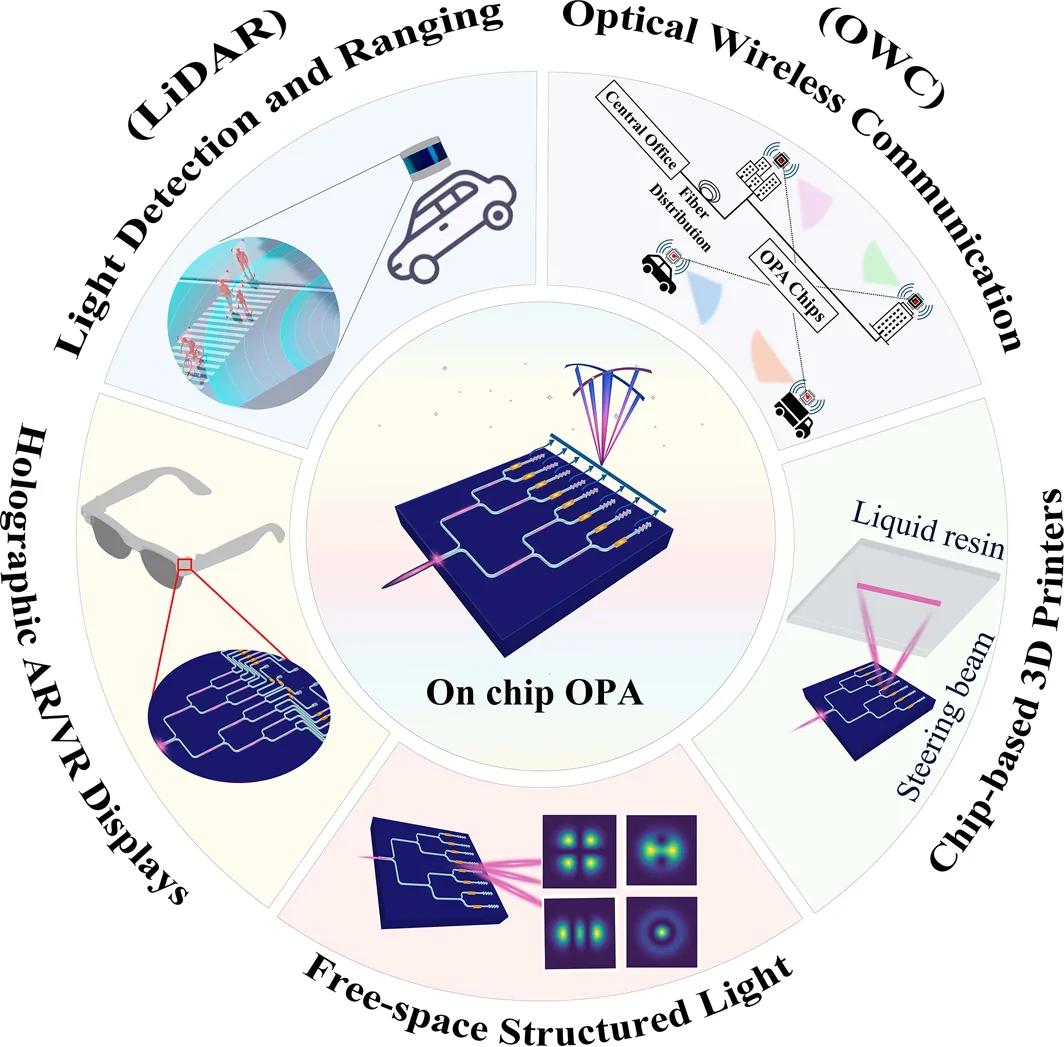

## 1 Introduction

Photonic integrated circuits (PICs) [1], often heralded as ”microelectronic chips of light,” aim to replicate within a single chip, the functions traditionally performed by bulk optical systems, including light generation, modulation, routing, processing, and detection. PIC technology enables the dense integration of hundreds of optical components, such as waveguides [2, 3, 4, 5, 6], modulators [7, 8, 9, 10], splitters [11, 12, 13], and filters [14, 15, 16], onto substrates with footprints on the order of square centimeters or smaller [17]. This unprecedented level of integration delivers transformative improvements in size, weight, power consumption, and cost (SWaP‐C) while also improving system stability and reliability. Such advances are opening new horizons across diverse fields ranging from high‐speed optical communications [18, 19] and high‐performance computing [20, 21] to sensing [22, 23] and quantum information processing [24, 25].

Among the many applications of PICs, on‐chip optical phased arrays (OPAs) stand out as a particularly compelling demonstration of integration and performance. OPAs steer and scan light beams in free space by precisely controlling the relative phases of multiple antenna elements, offering a nonmechanical route to rapid and accurate beam steering. Conventional bulk‐optic or fiber‐based phased‐array systems are typically large, complex, costly, and vulnerable to environmental perturbations. In contrast, PIC‐based OPAs integrate all the essential building blocks—laser sources, power distribution networks, phase modulators, optical antenna arrays, and control circuits—on a single chip or within a heterogeneously integrated platform. This miniaturized and scalable approach overcomes the intrinsic limitations of traditional architectures and paves the way for the mass production of compact, high‐performance beam scanners, with transformative implications for LiDAR [26, 27, 28, 29, 30, 31], free‐space optical communications [32], holographic displays [33], and biophotonic optical tweezers [34].

From the perspective of PIC technology, each major functional unit of an OPA can in principle be efficiently realized on‐chip. First, light sources and amplifiers, including III–V semiconductor lasers such as InP‐ or quantum‐dot‐based devices, can be heterogeneously integrated as on‐chip or external sources [35, 36, 37]. Second, power distribution networks based on low‐loss Si or SiN waveguides, configured as tree‐structured splitters or star couplers, can distribute light uniformly to hundreds of channels [11, 12, 13, 38]. Third, phase and amplitude modulators can be implemented using carrier‐dispersion effects in silicon [39, 40, 41] or the strong Pockels effect in ${\text{LiNbO}}_{3}$ [42, 43, 44, 45, 46]. Fourth, optical antennas, including grating couplers [47, 48] and edge emitters [49, 50], convert guided modes into free‐space beams with tailored radiation properties. Finally, drive and control electronics are increasingly being integrated with photonic OPAs; although several state‐of‐the‐art systems have already demonstrated electro‐photonic hybrid integration of CMOS driver circuits, the majority of current platforms still rely on off‐chip electronics, which continues to motivate scalable monolithic and heterogeneous integration of CMOS drivers with photonic devices.

Progress across diverse material platforms has highlighted complementary strengths. The silicon‐on‐insulator (SOI) platform offers unrivaled device density and miniaturization owing to its high index contrast, but its intrinsic two‐photon absorption limits optical power handling [51]. Silicon nitride (SiN) provides ultralow propagation loss and a broad transparency window, making it ideal for large‐scale distribution networks and high‐power applications, though it lacks strong electro‐optic tunability [52]. The indium phosphide (InP) platform has demonstrated the versatility and flexibility required for PICs with the most diverse component types. With methods including regrowth [53, 54], selective‐area growth [55], intermixing, and vertical active–passive integration [56], materials of different bandgaps, each individually and functionally optimized, can be integrated on the same substrate, providing the greatest flexibility in circuit floorplanning for large‐scale integration and very‐large‐scale integration [57]. Thin‐film ${\text{LiNbO}}_{3}$ (TFLN), with its outstanding electro‐optic coefficients, has recently emerged as a leading candidate for ultrafast, low‐power phase control, positioning itself as a rising star for next‐generation OPAs [58, 59, 60, 61].

In this review, we begin by outlining the operating principles of optical phased arrays and the performance metrics that define their capabilities. We then examine the fundamental building blocks of on‐chip OPAs—including integrated light sources, phase shifters, and grating couplers—highlighting recent advances in their design and implementation. This is followed by a survey of material platforms, from mature Si and ${\text{Si}}_{3}N_{4}$ technologies to hybrid Si–${\text{Si}}_{3}N_{4}$, emerging LNOI systems, and other alternatives, emphasizing their distinct advantages and limitations. We further analyze the major challenges that continue to constrain large‐scale deployment, such as electronic control overhead, crosstalk, and grating‐lobe suppression. Finally, we discuss representative applications and conclude with an outlook on future directions and opportunities for breakthroughs in on‐chip OPA technology.

## 2 On‐Chip OPA Architectures

On‐chip optical phased arrays (OPAs) can be categorized from two complementary perspectives: the spatial layout of the emitting elements, including one‐dimensional (1D) and two‐dimensional (2D) arrays, and the beam‐steering strategy, including electronic phase tuning and dispersive wavelength steering. To avoid ambiguity, the terms ”1D” and ”2D” in this section refer specifically to the dimensionality of the emitting array, whereas beam manipulation is described separately in terms of the steering mechanism or steering dimensionality. Based on these considerations, the representative on‐chip OPA architectures discussed below can be grouped into three categories: electronically driven 1D arrays for 2D beam steering, electronically driven 2D arrays with full element‐level control, and dispersive wavelength‐steered OPAs.

### 2.1 Electronically Driven 1D Arrays for 2D Beam Steering

In this architecture, the emitting elements are arranged in a one‐dimensional array, whereas two‐dimensional beam steering is achieved through a combination of active phase control along one axis and passive outcoupling or dispersive steering along the orthogonal axis. As illustrated in Figure 1a, beam steering along the array axis (e.g., the x‐direction) is typically realized by thermo‐optic or electro‐optic phase shifters integrated into each channel. The second steering dimension (e.g., the y‐direction) is then introduced not by a second electronically controlled array but by the emission structure itself, such as surface‐emitting gratings, fan‐shaped focusing gratings, or end‐fire line‐beam emitters. These outcoupling structures expand the radiation pattern into two dimensions and, in many cases, also provide wavelength‐dependent angular selectivity through their intrinsic dispersion. In such 1D‐array architectures enabling 2D steering, the electronically controlled axis provides high‐speed, fine‐resolution beam positioning through programmable phase tuning, whereas the orthogonal axis is mainly governed by the element factor and outcoupler dispersion, for example, through the grating equation and the effective grating length. This separation of roles substantially reduces circuit complexity, control‐channel count, and power consumption, making this class of architectures particularly attractive for large‐scale and hardware‐efficient OPA implementations.

**FIGURE 1:**
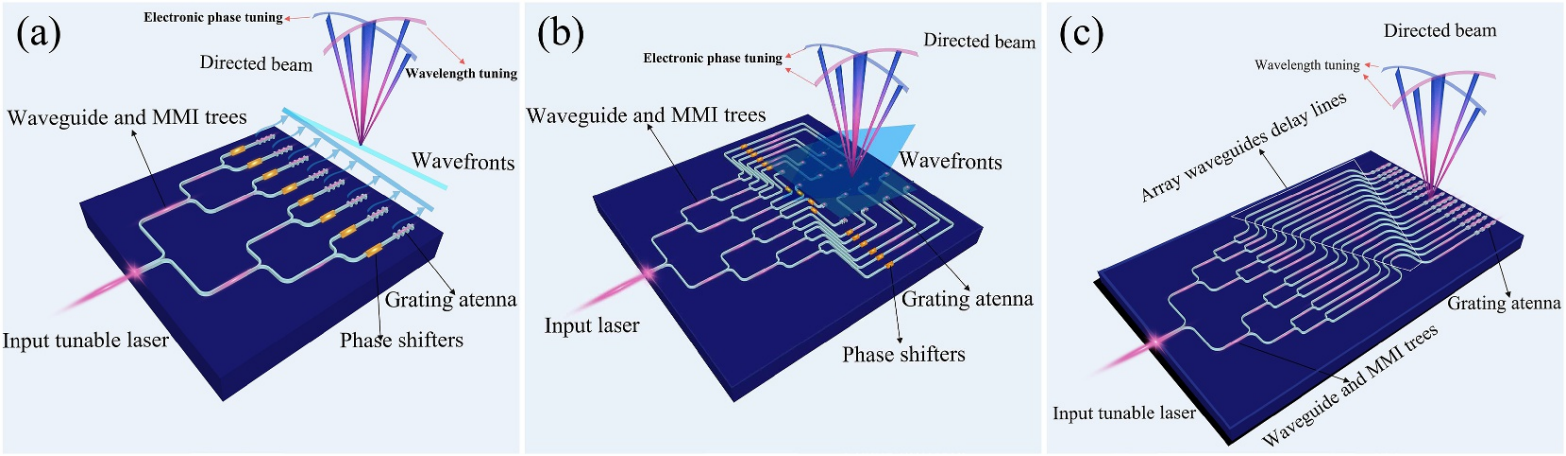
On‐chip optical phased‐array architectures and representative two‐dimensional beam‐steering schemes. (a) Electronically driven 1D array for two‐dimensional beam steering via combined phase and wavelength tuning; (b) electronically driven 2D array for two‐dimensional beam steering via phase modulation; (c) dispersive wavelength‐steered OPA architecture.

### 2.2 Electronically Driven 2D Arrays With Full Element‐Level Control

In contrast, this architecture adopts a two‐dimensional emitting array in which elements are distributed along both the x‐ and y‐directions, as shown in Figure 1b, and each element is equipped with independent phase and, in some cases, amplitude control. Such fully addressed 2D arrays enable genuinely symmetric two‐dimensional beam steering and provide a high degree of freedom for wavefront synthesis, thereby supporting functions such as wide‐angle scanning, multibeam generation, and advanced holographic beam shaping. However, these advantages come at the cost of substantially increased electronic overhead because individually addressing every emitter leads to a rapid growth in control‐channel count, total power consumption, electrical routing complexity, and thermal‐management difficulty. These factors currently remain the major constraints on the scalability of fully addressed 2D OPAs.

### 2.3 Dispersive Wavelength‐Steered OPAs

Complementary to electronically driven architectures, dispersive wavelength‐steered OPAs realize beam steering through the intrinsic wavelength dependence of integrated photonic structures, rather than through active per‐element phase tuning [62]. As illustrated in Figure 1c, this class of architectures maps wavelength‐dependent propagation and outcoupling dispersion into beam deflection, thereby enabling passive or semi‐passive steering without requiring independent electrical control of each emitter. Early demonstrations on silicon‐on‐insulator (SOI) combined highly dispersive arrayed‐waveguide gratings (AWGs) with surface grating couplers to achieve two‐dimensional wavelength‐controlled beam scanning, where the AWG defined one steering axis and grating‐coupler dispersion defined the other [63]. This concept was subsequently extended to more scalable architectures, such as serpentine OPAs (SOPAs), which embed long dispersive delay lines directly into meandering grating‐waveguide apertures to realize passive 2D scanning with a large number of resolvable spots [30], and pixelated dispersive OPAs (DOPAs), in which a continuous dispersive aperture is partitioned into multiple blocks fed by a balanced splitter tree [64]. On low‐loss silicon nitride (SiN) platforms, dispersive steering has also been implemented in both point‐beam and end‐fire line‐beam configurations using arrayed‐waveguide delay lines or Ω‐shaped routing networks, achieving configurable angular coverage and scan efficiencies on the order of 0.4–0.9°/nm while maintaining relatively low sidelobe levels [30, 65, 66, 67]. From a system perspective, such dispersive architectures are particularly attractive when minimizing electronic overhead and power consumption is prioritized, especially in swept‐source LiDAR systems. At the same time, their steering flexibility is fundamentally tied to laser wavelength and device dispersion, and long dispersive delay lines may introduce a larger footprint, excess insertion loss, and increased fabrication sensitivity. Therefore, dispersive OPAs are especially promising when simplified control and passive scalability outweigh the need for fully arbitrary, independent phase control over a large number of emitters.

## 3 Fundamental Principles and Performance Metrics

In this section, we introduce the fundamental beam‐steering principle of optical phased arrays (OPAs) and define the main performance indicators that will be used throughout this review, including angular resolution, field of view (FoV), main‐lobe efficiency, and sidelobe levels. For clarity, we focus on the canonical case of a one‐dimensional (1D) linear OPA and explicitly separate the roles of the element factor and the array factor. More advanced two‐dimensional architectures and dispersive wavelength‐steered OPAs will be discussed in later sections.

### 3.1 Beam‐Steering Principle of Optical Phased Arrays

Optical phased arrays (OPAs) are the optical counterparts of microwave phased arrays and can be viewed as programmable diffraction gratings whose responses are controlled via thermo‐optic or electro‐optic phase modulation. A simplified 1D OPA, illustrated in Figure 2, consists of N identical emitters with an interelement spacing of d along the x‐axis. All array elements are illuminated coherently by a common laser source so that their emitted fields interfere in the far field.

**FIGURE 2:**
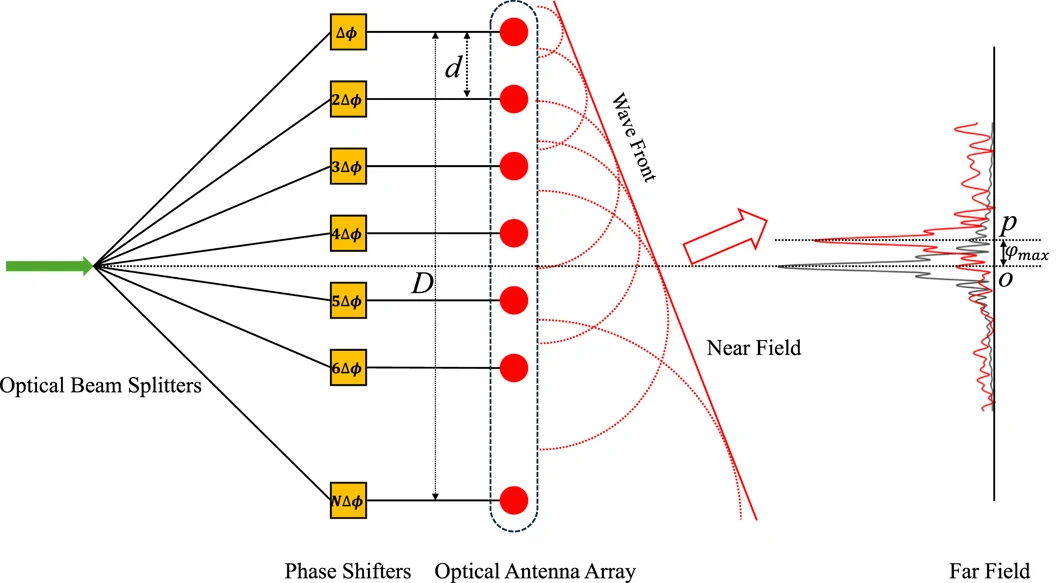
Structure and beam‐steering principle of a one‐dimensional optical phased array.

In the far field, the emission of an optical phased array (OPA) is well described by Fraunhofer diffraction and can be regarded as the complex Fourier transform of the near‐field distribution across the emitters [68]. A uniform (flat) phase profile over a finite aperture produces a single, narrow main lobe, whereas imposing a linear phase ramp across the array shifts the Fourier spectrum and thereby steers the beam to a desired angle. Thus, electronic phase tuning of the emitters provides nonmechanical beam steering. The steering range can be calculated using Equation (1), where Δϕ is the uniform phase difference between the signals emitted by adjacent array elements [69].

(1) $$\sin\;\varphi_{\max}=\lambda_{0}\Delta \phi /2d\pi$$

An alternative beam‐steering approach exploits the dispersive nature of surface‐emitting gratings, which are widely used in many on‐chip OPA implementations [44, 45, 70, 71, 72, 73, 74, 75, 76]. In these architectures, each channel incorporates a diffractive waveguide grating antenna (WGA) with an inherent wavelength–angle dispersion so that the emission direction becomes a function of the optical wavelength. Along the axis of the WGA, the outcoupling angle $\theta_{m}$ for the m‐th diffraction order is governed by the general grating equation:

(2) $$\sin\;\theta_{m}=\frac{\Lambda n_{\text{eff}}-m\lambda_{0}}{\Lambda n_{\text{clad}}}$$

where Λ is the grating period, $n_{\text{eff}}$ is the effective index of the waveguide at $\lambda_{0}$, $n_{\text{clad}}$ is the refractive index of the cladding material, and m is an integer denoting the diffraction order. Accordingly, sweeping the laser wavelength provides an additional, purely optical degree of freedom for beam steering, complementing or even replacing electronic phase control. It should be noted, however, that for a selected diffraction order, Equation (2) gives the corresponding outcoupling angle only for that order. In practical OPA designs, if multiple diffraction orders simultaneously satisfy the radiation condition within the visible angular range, multiple radiated beams may coexist. These additional beams reduce power efficiency, degrade beam quality, and limit the usable steering range. Therefore, ensuring single‐order operation through proper grating‐period design is critical for achieving high‐quality wavelength steering.

### 3.2 Element Factor, Array Factor, and Angular Resolution

In practice, the far‐field pattern of an OPA can be expressed as the product of the single‐element radiation pattern, namely, the element factor, and the array factor [77]:

(3) E(θ)=EF(θ)⋅AF(θ),

where EF(θ) describes the angular radiation envelope of an individual emitting element, and AF(θ) describes the interference pattern produced by the phased array. The resulting far‐field distribution can, therefore, be interpreted as array‐factor interference fringes modulated by the single‐element radiation envelope.

In Figure 3a–f, we use a simplified rectangular‐aperture model to illustrate how the element aperture affects the usable steering range. The fill ratio is defined as follows:

(4) $$F=\frac{a}{d},$$

where a is the effective emitting aperture width of a single array element along the array direction, and d is the center‐to‐center element pitch. In this calculation, d is fixed at 4μm, whereas a is prescribed as 1.6, 2.0, 2.4, 2.8, 3.2, and 3.6μm, corresponding to fill ratios of 40%, 50%, 60%, 70%, 80%, and 90%, respectively. Thus, in Figure 3, a is a swept input parameter in the element‐factor calculation rather than a value extracted from a specific grating‐antenna geometry. For practical grating antennas, a may be interpreted as an effective emitting width associated with the near‐field radiation profile, rather than necessarily the lithographic width of the antenna.

**FIGURE 3:**
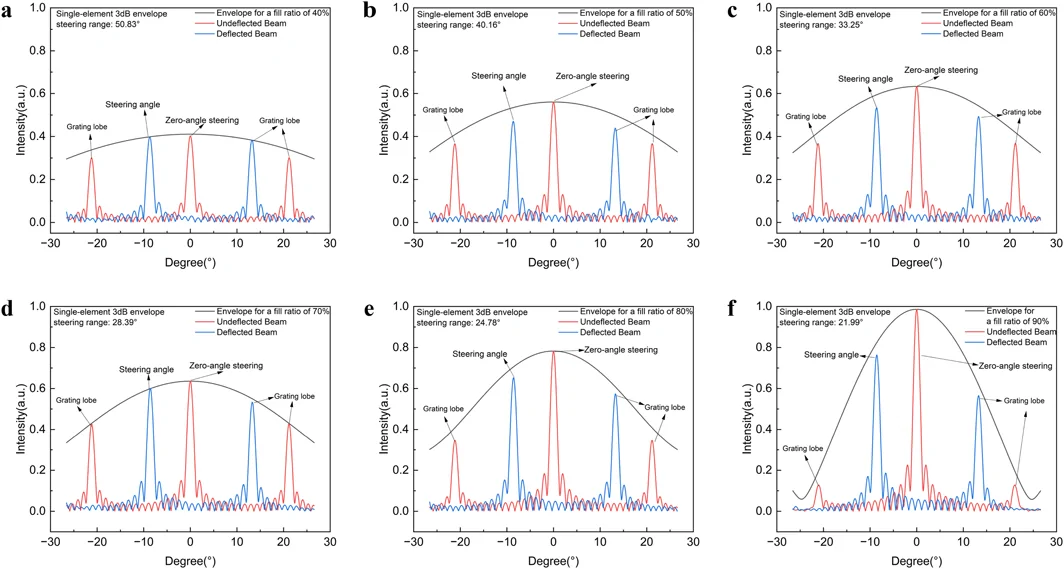
Far‐field patterns modulated by the element factor for different fill ratios. The element pitch is fixed at 4μm, whereas the element width increases from 1.6 to 3.6μm, corresponding to fill ratios of (a) 40%, (b) 50%, (c) 60%, (d) 70%, (e) 80%, and (f) 90%.

The label ”single‐element 3 dB envelope” in Figure 3 denotes the angular region in which the normalized single‐element radiation intensity satisfies the following equation:

(5) $$\frac{|EF\left(\theta \right){|}^{2}}{\max_{\theta }|EF\left(\theta \right){|}^{2}}\geq 0.5.$$

It therefore refers to the 3‐dB angular envelope of the single‐element radiation pattern, not to a specific array element or to the 3‐dB width of the full array factor.

The usable steering range indicated in each panel is quantitatively determined as the overlap between the single‐element 3‐dB angular envelope and the grating‐lobe‐free angular range imposed by the array factor. Specifically, the single‐element 3 dB steering range is defined as follows:

(6) $$\Omega_{\text{EF,}3\text{dB}}=\left\{\theta \;|\;\frac{|EF\left(\theta \right){|}^{2}}{\max_{\theta }|EF\left(\theta \right){|}^{2}}\geq 0.5\right\},$$

and the usable steering range is obtained as follows:

(7) $$\Omega_{\text{steering}}=\Omega_{\text{EF,}3\text{dB}}\cap \Omega_{\text{AF,GL}‐\text{free}},$$

where $\Omega_{\text{AF,GL}‐\text{free}}$ denotes the angular range in which no grating lobe appears in the visible angular region. Because the operating wavelength and element pitch d are fixed in Figure 3, the array‐factor‐limited grating‐lobe‐free range remains unchanged across Figure 3a–f. By contrast, increasing a, or equivalently increasing the fill ratio, narrows the single‐element 3‐dB envelope and concentrates more energy into the main lobe. Therefore, a larger fill ratio improves the single‐element directivity but reduces the usable steering range.

#### 3.2.1 Beam Width

In this section, the *lateral* direction refers to the array‐expansion direction defined by the transverse distribution of emitters, whereas the *longitudinal* direction refers to the grating‐emission direction along the propagation length of an individual emitter. The lateral beamwidth, $\Delta \phi_{\text{FWHM}}$ (°), quantifies the angular width of the main lobe along the lateral direction; a smaller $\Delta \phi_{\text{FWHM}}$ corresponds to a more directive beam [69, 78]. For a uniformly spaced OPA with an effective aperture of Nd, the lateral full width at half maximum (FWHM) can be approximately expressed as follows:

(8) $$\Delta \phi_{\text{FWHM}}=\frac{0.886\lambda_{0}}{Nd\;\cos\;\phi }$$

where $\lambda_{0}$ is the operating wavelength, N is the number of emitters, d is the interelement spacing, and ϕ is the steering angle. Equation (8) shows that the lateral beamwidth scales inversely with the effective lateral aperture Nd. Therefore, increasing the aperture leads to a narrower main lobe and a more directive beam. However, this improvement in angular resolution is accompanied by important trade‐offs with the field of view (FoV).

In the context of OPAs, the FoV refers to the angular range over which the main beam can be steered while maintaining acceptable beam quality. The lateral FoV is defined along the array‐expansion direction and is mainly limited by the grating‐lobe‐free steering range. Along this direction, if the effective lateral aperture is enlarged by increasing the element spacing d while keeping the number of emitters N fixed, the larger pitch reduces the grating‐lobe‐free steering range and therefore constrains the achievable lateral FoV [79]. At the same fixed emitter number N, reducing the element spacing d has the opposite effect: It helps preserve a wider grating‐lobe‐free lateral FoV, but it also reduces the effective aperture Nd and therefore broadens the main lobe. In addition, a smaller interwaveguide spacing can intensify optical crosstalk, increase power dissipation, and degrade beamforming efficiency.

The longitudinal FoV, by contrast, corresponds to the steering range along the direction orthogonal to the array‐expansion direction, and its physical origin depends on the specific OPA architecture. For two‐dimensional phased arrays, the longitudinal FoV is governed by the aperture, emitter spacing, and phase progression along the longitudinal direction, analogous to the lateral case. In this architecture, both the lateral and longitudinal FoVs are determined by the phased‐array parameters in their respective directions, including the phase progression, emitter number, and element spacing. For one‐dimensional OPAs employing wavelength‐assisted beam steering, however, the two directions have different physical origins: The lateral FoV is governed by the phased‐array parameters, whereas the longitudinal FoV is mainly determined by the grating‐emitter design, the available wavelength‐tuning range, and the angular dispersion response of the emitter.

Accordingly, increasing the emitter number N provides a way to relax this aperture–FoV trade‐off, but its role should be interpreted according to the design constraint and steering mechanism. When the design goal is to enlarge the effective aperture, increasing N allows the aperture to be increased by adding more emitters, rather than by increasing the element spacing d alone. This enables d to be kept within a range compatible with grating‐lobe suppression. In two‐dimensional phased arrays, this principle applies to both the lateral and longitudinal directions: Increasing $N_{x}$ or $N_{y}$ enlarges the corresponding effective aperture, $N_{x}d_{x}$ or $N_{y}d_{y}$ while avoiding an excessively large element pitch. In one‐dimensional OPAs with wavelength‐assisted steering, this argument mainly applies to the lateral phased‐array direction, where increasing N enlarges the effective lateral aperture without requiring a large increase in d. As illustrated in Figure 4, this strategy improves angular resolution while preserving the grating‐lobe‐free steering range. If the total aperture is instead fixed, increasing N would reduce the element spacing d, which represents a different design condition. Nevertheless, scaling N to very large values introduces substantial overhead in driver count, power consumption, and real‐time calibration. Consequently, the design of high‐performance OPAs requires a careful balance among aperture size, interelement spacing, field of view, and system‐level electronic complexity, with the relevant trade‐offs depending on the specific OPA architecture.

**FIGURE 4:**
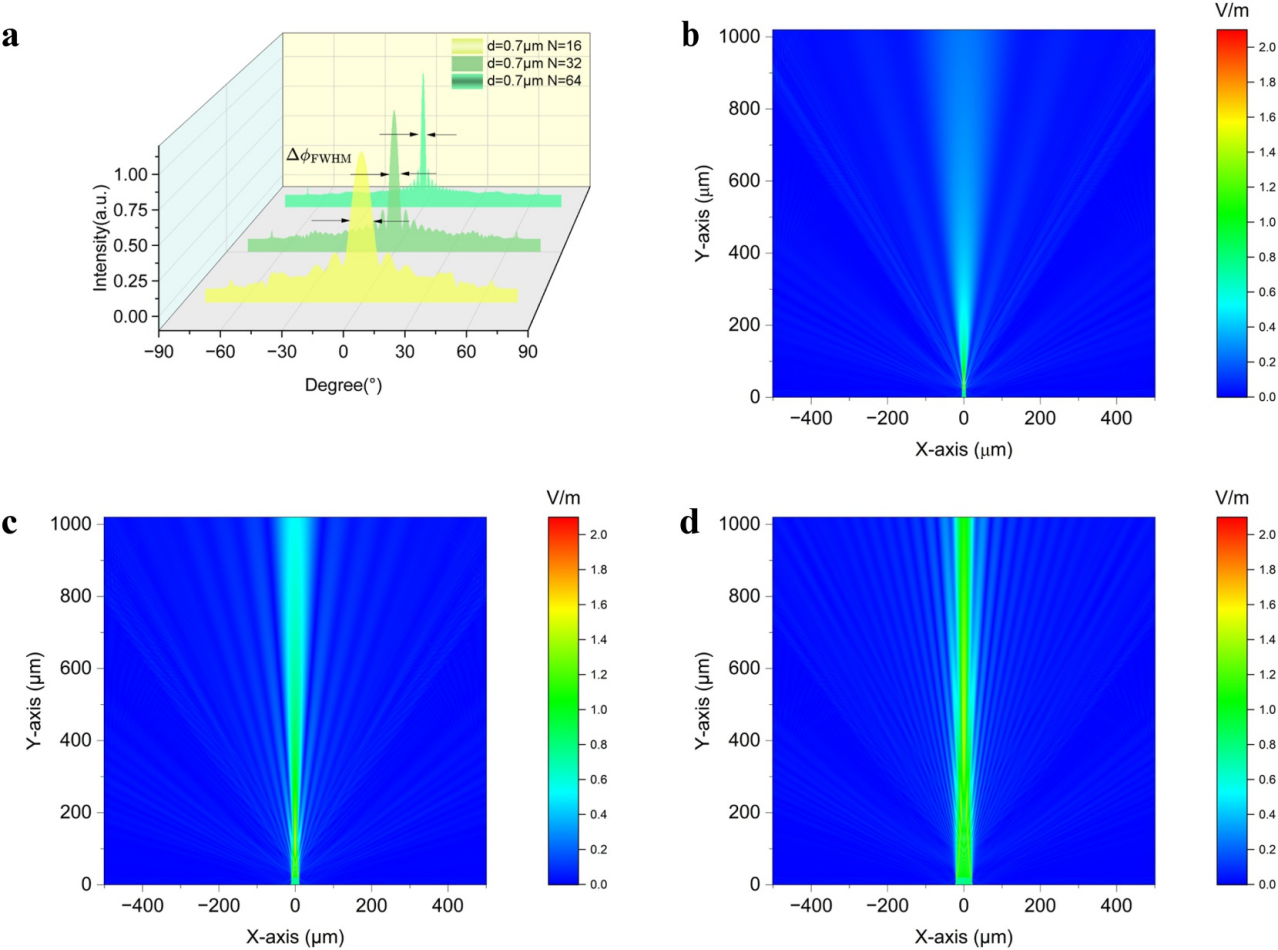
Beam widths for different numbers of elements, N. (a) Far‐field patterns; (b) beam divergence angles simulation results for a 16‐element array; (c) beam divergence angles simulation results for a 32‐element array; (d) beam divergence angles simulation results for a 64‐element array.

The longitudinal beamwidth, $\Delta \theta_{\text{FWHM}}$ (°), quantifies the angular extent of the beam along the longitudinal direction, namely, the grating‐emission direction. When steering in this dimension is generated collectively by multiple emitters distributed along the longitudinal axis, the corresponding beamwidth can be estimated by modifying Equation (8) accordingly. Otherwise, $\Delta \theta_{\text{FWHM}}$ is primarily governed by the properties of the individual grating emitter, including the grating geometry, effective emission length, and operating wavelength.

### 3.3 Aperiodic Optical Phased‐Array Theory

In optical phased arrays (OPAs), grating lobes can reach intensities comparable to the main lobe, giving rise to parasitic beams at unintended angles. These spurious beams introduce angular ambiguity in beam steering, reduce the contrast and purity of the desired main beam, and redistribute optical power away from the intended direction, thereby degrading the fidelity of the emitted beam profile. Complete suppression of grating lobes across the visible angular range requires the element spacing to satisfy d≤λ/2, as illustrated in Figure 5. Although this condition is readily achievable in microwave arrays, it becomes a major fabrication challenge at optical frequencies, where submicron wavelengths demand deep‐submicron routing, exacerbate optical crosstalk, and impose stringent lithographic tolerances.

**FIGURE 5:**
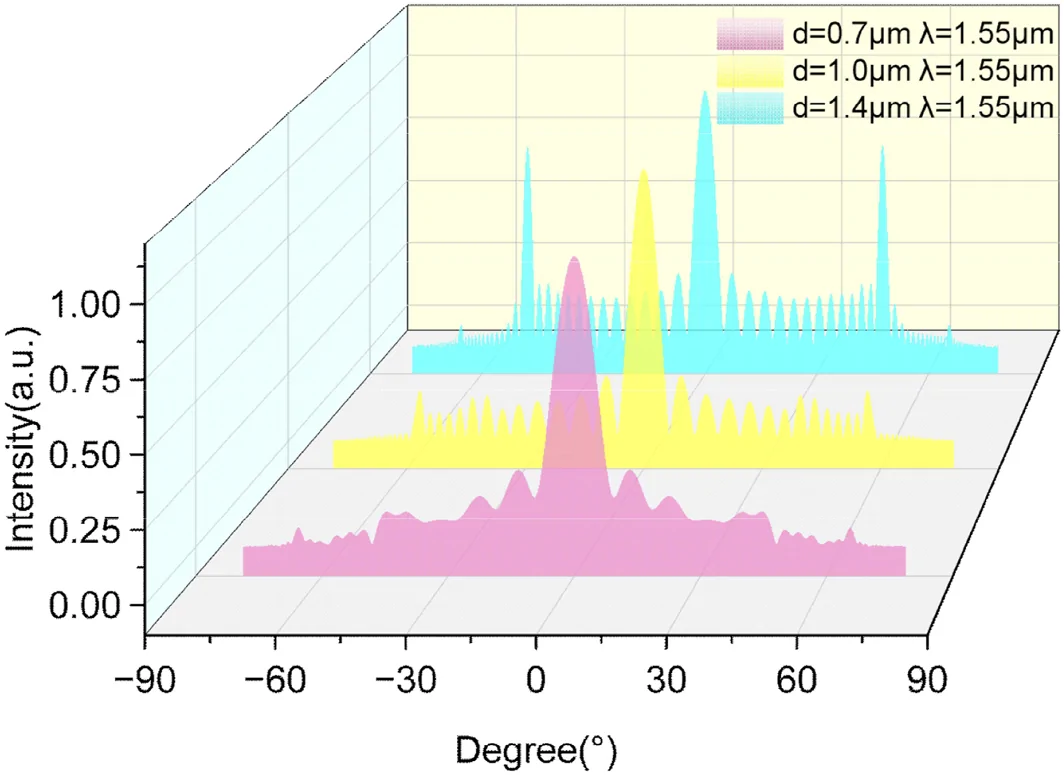
Far‐field distributions with different element spacings.

A commonly adopted mitigation strategy is to relax strict periodicity and introduce nonuniform element spacing. Even small deviations from uniform pitch decorrelate the angular positions of grating lobes generated by different emitter pairs, thereby reducing their coherent reinforcement. However, such perturbative approaches generally weaken grating lobes rather than eliminate them. This limitation motivates the more rigorous framework of aperiodic optical phased arrays, in which emitter locations are deliberately engineered to reshape the underlying spatial‐frequency spectrum.

A central theoretical insight developed in recent studies [80] is that, up to a global translation‐induced phase factor, the far‐field intensity of an OPA is governed not by the absolute emitter coordinates themselves but by the complete set of pairwise displacement vectors:

(9) $$\Delta_{n,m}=r_{n}-r_{m},$$

where $r_{n}$ denotes the position vector of the n‐th emitter. For a discrete array with complex excitation coefficients $C_{n}$, the far‐field intensity I(ξ) can be expressed as the Fourier transform of the aperture autocorrelation function:

(10) $$R_{AA}\left(\Delta \right)=\sum_{n=1}^{N}\sum_{m=1}^{N}C_{n}C_{m}^{*}\;\delta \left(\Delta -\Delta_{n,m}\right),$$

where Δ is a generic pairwise displacement vector, δ(⋅) is the Dirac delta function, and ξ denotes the normalized transverse spatial‐frequency coordinate in the far field. As illustrated in Figure 6, Equation (9) describes the set of all pairwise baselines generated by the emitter positions, whereas Equation (10) maps their statistical distribution into the aperture autocorrelation function. The far‐field pattern is, therefore, determined by how these displacement vectors are distributed, which, in turn, governs the beamwidth, sidelobe structure, and angular resolving capability of the array. For a detailed derivation, the reader is referred to Ref. [80].

**FIGURE 6:**
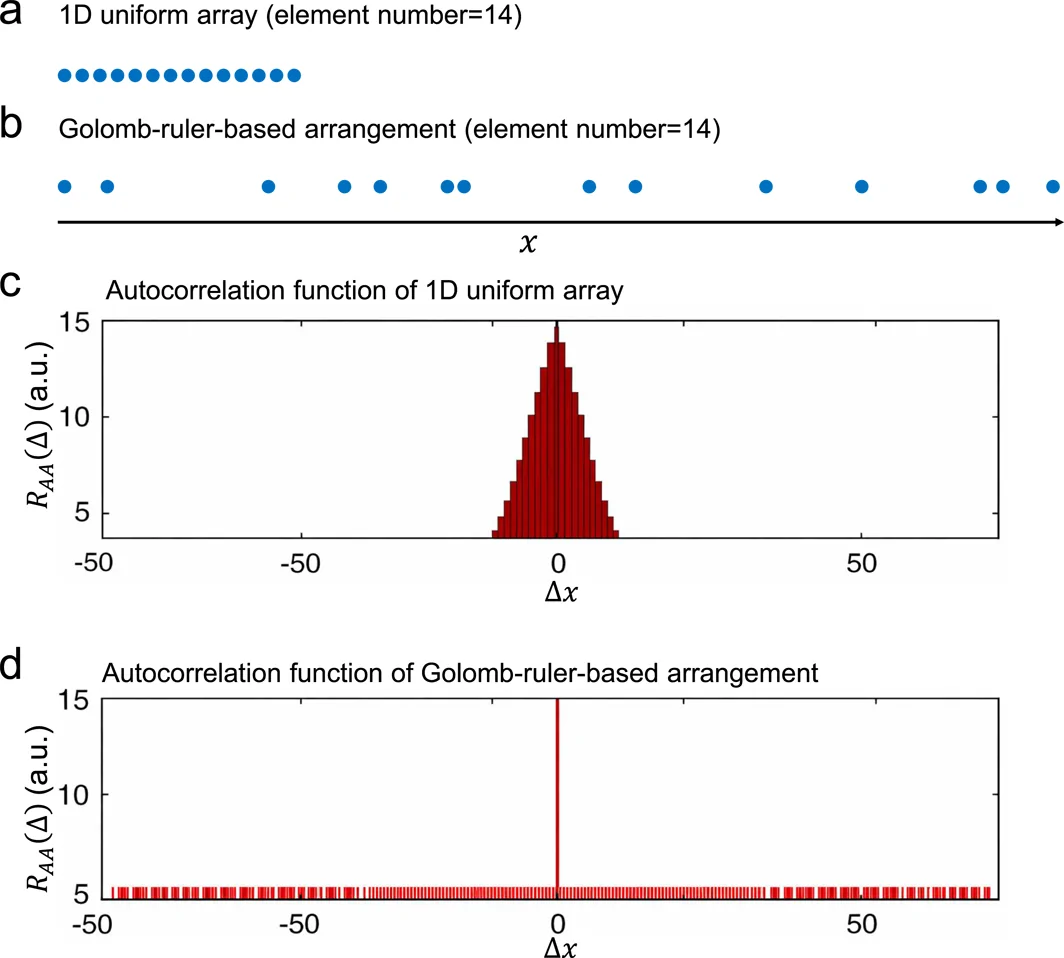
Illustration of the theoretical framework of aperiodic optical phased arrays [80]. (a) Uniformly spaced array, in which many emitter pairs share identical displacement vectors $\Delta_{n,m}=r_{n}-r_{m}$; (b) Golomb‐ruler array, in which emitter positions are arranged to maximize the diversity of pairwise displacement vectors; (c) aperture autocorrelation function $R_{AA}$ of the 1D uniform array; (d) aperture autocorrelation function $R_{AA}$ of the 1D Golomb‐ruler array.

In periodic OPAs, the set of displacement vectors $\Delta_{n,m}$ is highly redundant because many emitter pairs share identical separations. Consequently, the aperture autocorrelation collapses into a sparse and strongly structured distribution, whose Fourier transform produces discrete and regularly spaced peaks in the spatial‐frequency domain, appearing as strong grating lobes in the far field. By contrast, aperiodic OPAs deliberately maximize the diversity of $\Delta_{n,m}$ through nonredundant or weakly redundant emitter placements [80]. In the ideal nonredundant limit, the number of unique baselines approaches $O\left(N^{2}\right)$, causing the aperture autocorrelation to broaden into a much more extended and quasi‐continuous distribution. Its Fourier transform correspondingly fills the spatial‐frequency domain in a dense and nonperiodic manner, suppressing the coherent buildup of grating lobes and replacing discrete grating‐lobe peaks with a much lower and more uniform background floor. This principle underlies classical nonredundant array configurations, such as one‐dimensional Golomb‐ruler and two‐dimensional Costas arrays [80, 81].

Figure 6a,b highlights the fundamental difference in emitter placement between a uniform array and a Golomb‐ruler array. In a uniform array, emitters are arranged with a constant pitch, so the set of pairwise displacement vectors in Equation (9) contains a large number of repeated values, meaning that many different emitter pairs share identical separations. By contrast, the Golomb‐ruler array employs a nonredundant placement strategy in which the spacing between any pair of emitters is made as distinct as possible, thereby maximizing the diversity of the displacement‐vector set. In this sense, Figure 6a corresponds to a highly redundant $\Delta_{n,m}$ distribution, whereas Figure 6b represents a largely nonredundant one.

This structural difference is directly reflected in the aperture autocorrelation function of Equation (10), which can be understood as a weighted distribution of all pairwise displacement vectors in the array. Because $R_{AA}\left(\Delta \right)$ is determined by how frequently different $\Delta_{n,m}$ values occur, the degree of redundancy in the displacement‐vector set directly governs whether the autocorrelation is narrowly concentrated or broadly distributed. Correspondingly, Figure 6c,d compares the autocorrelation functions of the two array types. For the uniform array, many emitter pairs contribute to the same displacement values, so these repeated terms coherently accumulate at a limited number of Δx positions, producing the narrow and highly concentrated triangular distribution shown in Figure 6c. This indicates that the spatial‐frequency content of the uniform array is highly redundant, which, in turn, gives rise to strong and regularly spaced grating lobes in the far field.

In contrast, the Golomb‐ruler array largely eliminates repeated pairwise displacements, so its autocorrelation function no longer collapses into a few heavily populated Δx positions. Instead, as shown in Figure 6d, $R_{AA}\left(\Delta \right)$ is spread nearly uniformly over a much broader range. Apart from the sharp peak at Δx=0, which inevitably arises from the self‐correlation terms (n=m), the remaining displacement components are distributed across a wide interval. Physically, this means that the diffractive replicas generated by different emitter pairs no longer overlap coherently at a few fixed angular positions but are instead dispersed into many weak and spatially mismatched contributions. As a result, the strong grating‐lobe peaks characteristic of periodic arrays are replaced by a much lower and nearly uniform grating‐lobe noise floor.

From a broader perspective, this comparison illustrates that nonredundant arrays reshape the far‐field spatial‐frequency spectrum through displacement‐vector engineering. Compared with uniform arrays, the enhanced diversity of $\Delta_{n,m}$ in Golomb‐ruler arrays broadens the autocorrelation support and enriches the accessible spatial‐frequency content. Because beamwidth is inversely related to spatial‐frequency bandwidth, this effect not only suppresses coherent grating‐lobe buildup but also improves the spatial resolving capability of the array. As a result, the number of resolvable points can scale from the conventional N‐dependent behavior of uniformly spaced OPAs toward nearly $N^{2}$ in nonredundant array configurations.

Furthermore, theoretical and numerical analyses indicate that the grating‐lobe noise floor in aperiodic OPAs depends primarily on the number of channels N, rather than on the average pitch alone [81]. Even when the array becomes extremely sparse, the noise floor remains nearly unchanged, whereas the effective aperture can continue to grow. This property enables ultrasparse aperiodic OPAs to achieve simultaneously large FoV and narrow beam divergence with substantially fewer channels, thereby reducing the complexity of electrical control and wire bonding.

Taken together, these results establish the key theoretical advantage of aperiodic OPAs: By engineering the distribution of displacement vectors to be maximally diverse and nonredundant, the spatial‐frequency spectrum can be broadened, coherent grating‐lobe formation can be suppressed, and the conventional coupling among beamwidth, element spacing, and FoV can be substantially relaxed. This provides a powerful route toward large‐aperture, high‐resolution, and scalable beam‐steering systems.

## 4 Key Building Blocks of On‐Chip OPAs

### 4.1 On‐Chip Laser Source

The performance of on‐chip optical phased‐array (OPA) systems is closely linked to the characteristics of the optical source, particularly its output power, linewidth, wavelength tunability, and integration compatibility. These parameters directly affect the beam‐steering range, angular resolution, coherence performance, and overall system stability. In wavelength‐steered or hybrid phase–wavelength OPA architectures, the tuning precision and accessible spectral range of the laser source largely determine the achievable spectral scanning resolution and field of view. By contrast, in phase‐controlled OPA systems, source stability and spectral purity are especially important for maintaining beam quality and reducing phase noise accumulation across the array.

At present, many OPA demonstrations still rely on off‐chip laser sources, especially when high‐performance tunable lasers are required. External laser injection can be implemented through grating couplers or edge couplers. Grating couplers offer layout flexibility and wafer‐level accessibility but usually suffer from strong polarization sensitivity and pronounced wavelength dependence. Edge couplers, by contrast, generally provide higher coupling efficiency, broader bandwidth, and lower wavelength sensitivity, with reported efficiencies exceeding 80% [82, 83]. However, they require high‐quality facet processing and impose additional constraints on‐chip packaging and alignment [84]. As a result, although off‐chip sources remain indispensable in many experimental systems, their use inevitably compromises compactness, coupling stability, and scalability.

These limitations have made on‐chip laser integration a central direction in the development of OPA systems [85]. In parallel, improving the interfacing efficiency between external high‐power lasers and OPA chips has also emerged as a practical transitional route toward compact and power‐efficient beam‐steering modules. Fowler et al. demonstrated a high‐power source‐interfacing strategy by directly coupling a 24‐channel SiN OPA to a 905‐nm tapered InGaAsP diode laser [86]. By illuminating the full waveguide array through a slab‐input region, this approach bypassed the conventional splitter‐tree network, thereby reducing distribution loss and structural complexity while enabling 2 W continuous‐wave and multiwatt pulsed operation for medium‐range time‐of‐flight LiDAR [86]. Although such schemes do not constitute true on‐chip source integration, they highlight the importance of source–OPA codesign from a system perspective.

Early progress in on‐chip source integration mainly focused on demonstrating the feasibility of cointegrating the optical source and beam‐steering circuitry on the same photonic platform. In 2012, Doylend et al. reported a hybrid III–V/silicon OPA that integrated an on‐chip laser source, an SOA preamplifier, a 1×8 multimode interferometer splitter, phase‐shifting channels, and grating emitters into a complete source‐to‐radiation chain [87]. Within this architecture, the generated coherent light is amplified, distributed, phase tuned, and radiated into free space. The measured laser characteristics showed single‐wavelength operation near 1594.82 nm, with a linewidth below 40 MHz and a side‐mode suppression ratio of about 30 dB, thereby validating the feasibility of on‐chip source integration for OPA beam steering [87]. Guo et al. further extended the InP‐based OPA architecture by monolithically integrating a widely tunable sampled‐grating distributed Bragg reflector (SGDBR) laser on the same photonic integrated circuit [88]. As shown in the upper panel of Figure 7a, the PIC layout incorporates the on‐chip SGDBR laser at the input side of the beam‐steering circuit, enabling the optical source, amplification, phase control, and emission functions to be combined within a unified InP platform [88]. Compared with the earlier externally driven configuration, this architecture eliminates the reliance on an off‐chip tunable laser and therefore represents a more complete realization of an integrated beam‐steering source. The source integration is further evidenced by the lower panel of Figure 7a, which presents the output spectra of the on‐chip SGDBR laser. By tuning the front and back mirror sections, different laser supermodes can be selectively accessed, and the device is expected to provide a total tuning range close to 30 nm.

**FIGURE 7:**
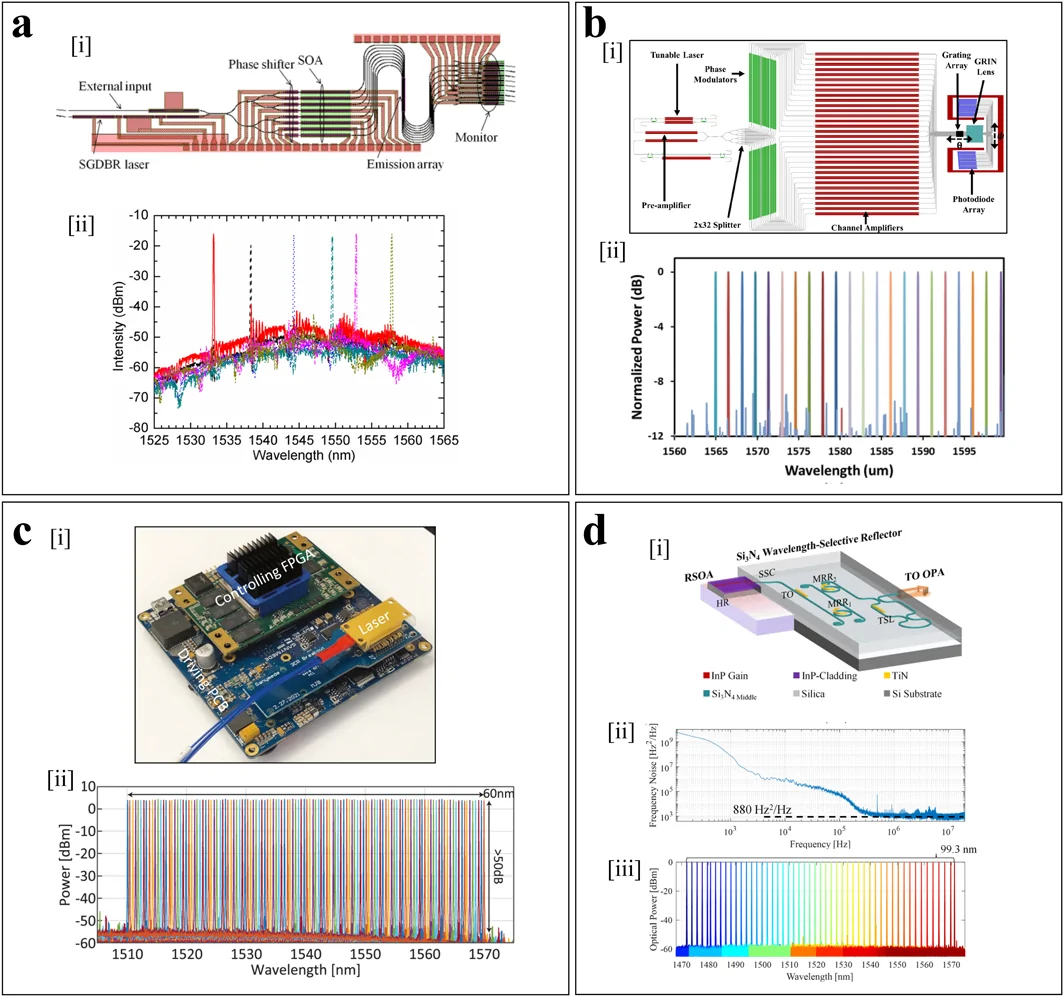
Optical phased array with an on‐chip laser source. (a) Monolithically integrated SGDBR laser source for an InP‐based optical beam‐steering PIC. The figure shows [i] the updated circuit layout with an on‐chip tunable SGDBR laser and [ii] the corresponding output spectra. The integrated laser exhibits tunable supermodes with an expected total tuning range close to 30 nm [88]. (b) Fully integrated hybrid silicon beam scanner with an on‐chip tunable laser. The figure shows [i] the integrated source‐to‐radiation architecture of the device together with [ii] representative optical spectra of the embedded laser [89]. (c) Driving laser designed for OPA‐based coherent LiDAR. The figure shows [i] the laser architecture based on a silicon‐photonic cavity with integrated tuning, chirp control, and feedback functionalities, together with the measured overlaid spectra. [ii] A tuning range of approximately 60 nm and a side‐mode suppression ratio of about 50 dB are achieved, demonstrating the wavelength agility and spectral purity of the source [29]. (d) Hybrid‐integrated on‐chip external cavity laser (ECL) for OPA‐based LiDAR. The figure shows [i] the laser architecture together with representative performance, including an RSOA‐based hybrid external cavity using a silicon nitride Vernier wavelength‐selective reflector, [ii] the frequency noise spectrum of the ECL, an intrinsic linewidth of approximately 2.8 kHz, and [iii] widely tunable single‐mode lasing from 1471.92 to 1571.18 nm with a worst‐case side‐mode suppression ratio of 42.6 dB [76].

Subsequent efforts moved beyond proof‐of‐concept integration toward a wider tuning range and more functional beam‐steering capability. Hulme et al. demonstrated a fully integrated hybrid silicon two‐dimensional beam scanner with directly incorporated tunable on‐chip lasers [89]. As shown in the upper panel of Figure 7b, the device integrates the optical source, preamplification, power splitting, phase control, channel amplification, and grating emission on a single chip. The lower panel of Figure 7b shows that the integrated laser provides a 34.5‐nm tuning range with a tuning efficiency of 0.28 nm/mW, supporting 23°×3.6° beam steering with a beamwidth of 1°×0.6° [89]. The field further advanced with the demonstration of a monolithically integrated erbium‐doped laser‐driven OPA in 2019, which achieved a side‐mode suppression ratio of 30 dB and 40 mW output power while enabling a beamwidth of 0.85°×0.2°, marking the first integration of rare‐earth‐doped lasers with CMOS‐compatible silicon photonics [90]. In 2020, a quantum‐dot reflective semiconductor optical amplifier (RSOA) coupled to a ${\text{Si}}_{3}N_{4}$ cavity achieved an 85‐kHz linewidth and a 47‐nm tuning range, supporting wavelength steering through waveguide gratings [91].

More recently, the development of on‐chip laser sources has become increasingly driven by system‐level requirements, particularly those of coherent and FMCW LiDAR. Poulton et al. demonstrated a laser architecture tailored for OPA‐based coherent LiDAR, highlighting the growing importance of linewidth, tunability, and chirp linearity in practical beam‐steering systems [29]. As shown in the upper panel of Figure 7c, the source is built around a silicon‐photonic cavity with integrated tuning, chirp control, and feedback functions and is packaged together with custom driving electronics as a LiDAR‐oriented laser module. The lower panel of Figure 7c shows that the device simultaneously achieves a tuning range of about 60 nm, a linewidth of approximately 50 kHz, and a linear chirp of 1.3 GHz over 17.5 μs. These characteristics were later leveraged in a system‐level demonstration of an 8192‐element CMOS‐driven OPA with a 1‐μm pitch, enabling a field of view of 100°×17° [29]. This trend indicates that on‐chip laser development for OPAs is no longer limited to simple source integration but is increasingly co‐optimized with beam‐steering architecture, driving electronics, and ranging modality.

Recent work has also explored compact hybrid tunable laser integration for OPA transmitters. For example, Misugi et al. integrated a hybrid wavelength‐tunable laser diode with a 64‐channel passive OPA on a combined chip [92]. Using a Vernier‐based external cavity formed by a semiconductor optical amplifier, a loop mirror, and two ring resonators with different circumferences, the source achieved stable single‐mode lasing from 1496 to 1592.5 nm with a side‐mode suppression ratio above 35 dB [92]. Xu et al. further demonstrated a hybrid external cavity laser on a multilayer silicon‐nitride‐on‐silicon platform [76]. As shown in Figure 7d, the source integrates an RSOA with a silicon nitride wavelength‐selective reflector based on Vernier microrings and a tunable Sagnac loop mirror, forming a compact cavity compatible with direct OPA integration. The measured performance shows an intrinsic linewidth of about 2.8 kHz and widely tunable single‐mode lasing from 1471.92 to 1571.18 nm, with a worst‐case side‐mode suppression ratio of 42.6 dB [76]. Such simultaneous achievement of an ultranarrow linewidth and broad tunability is particularly attractive for wavelength‐steered and coherent OPA systems.

Overall, the evolution of on‐chip laser sources for OPAs has progressed from early demonstrations of source–beam‐steering cointegration to tunable and functional integrated sources and further toward LiDAR‐oriented high‐performance laser modules. Across these developments, output power, linewidth, and tunability have emerged as the three most critical source parameters governing OPA performance. Future progress will depend not only on improving these metrics individually but also on jointly optimizing source efficiency, manufacturability, packaging compatibility, and cointegration with amplifiers and driving electronics. In this sense, scalable and low‐cost on‐chip laser integration remains a key prerequisite for the widespread deployment of OPA systems in automotive LiDAR and other emerging applications.

### 4.2 Phase Shifters

The beam‐steering performance of optical phased arrays (OPAs) is fundamentally governed by the response speed and energy efficiency of their constituent phase shifters. These critical components can be broadly classified into thermo‐optic (TO) and electro‐optic (EO) modulators according to their operating principles, with their distinct characteristics directly determining the overall power consumption, modulation bandwidth, thermal stability, and scalability of OPA systems.

#### 4.2.1 Thermo‐Optic Phase Shifters

TO phase modulation exploits the thermorefractive effect, whereby localized heating of a waveguide induces a refractive‐index change. In silicon photonics, this effect is particularly pronounced, with a thermo‐optic coefficient of $dn/dT=1.87\times 10^{-4}\;K^{-1}$ at 1500 nm and 295 K [93]. Conventional TO phase shifters typically employ metallic microheaters (e.g., Ti/Au, TiN) or doped silicon resistors to generate Joule heating [94, 95, 96, 97, 98]. These devices benefit from fabrication simplicity, broad material compatibility, and low intrinsic optical loss; however, their modulation bandwidth is fundamentally limited by thermal diffusion dynamics [39, 99].

A major drawback of TO phase shifters lies in their relatively high power consumption. Standard designs typically require ∼24.4 mW/π, which poses a critical challenge for large‐scale OPA integration due to cumulative power dissipation, thermal crosstalk, and long‐term reliability concerns [100]. In unoptimized configurations, the required power can increase to 50 mW/π [101], further exacerbating these limitations. Accordingly, extensive efforts have been devoted to improving TO phase‐shifter efficiency through resonant enhancement, heater and material engineering, geometrical heat management, and thermal isolation.

Beyond conventional nonresonant straight‐waveguide heaters, one alternative route is to exploit resonantly enhanced phase control in compact microresonator structures. An example was reported by Chalupnik et al., who demonstrated a two‐dimensional silicon‐photonic phased array based on strongly overcoupled microring resonator phase shifters [102]. Compared with conventional straight‐waveguide TO phase shifters, this resonator‐based approach combines a very small device footprint with low‐power operation while maintaining full 2π phase‐shift capability. In their 8×8 array, each unit cell occupied only $15\times 15\;\mu m^{2}$, and the microring phase shifter had an external radius of approximately 3 μm. The device achieved an average power consumption of about 300 μW per element and a modulation speed of about 330 kHz [102].

For nonresonant TO phase shifters, a major direction has been to strengthen the overlap between thermal perturbation and the guided optical mode through heater and material engineering. One notable approach employs a graphene microheater directly integrated with a silicon‐photonic crystal waveguide operating in the slow‐light regime [103]. In this architecture, as shown in Figure 8a, direct graphene–waveguide contact improves heat transfer, whereas slow light enhances the effective phase response per unit heating power [103]. As a result, the device achieves a tuning efficiency of up to 1.07 nm ${\text{mW}}^{-1}$ and a power consumption of about 3.99 mW per free spectral range, as shown in Figure 8a. However, the optical absorption associated with graphene also introduces additional insertion loss, highlighting a persistent trade‐off between tuning efficiency and optical transparency.

**FIGURE 8:**
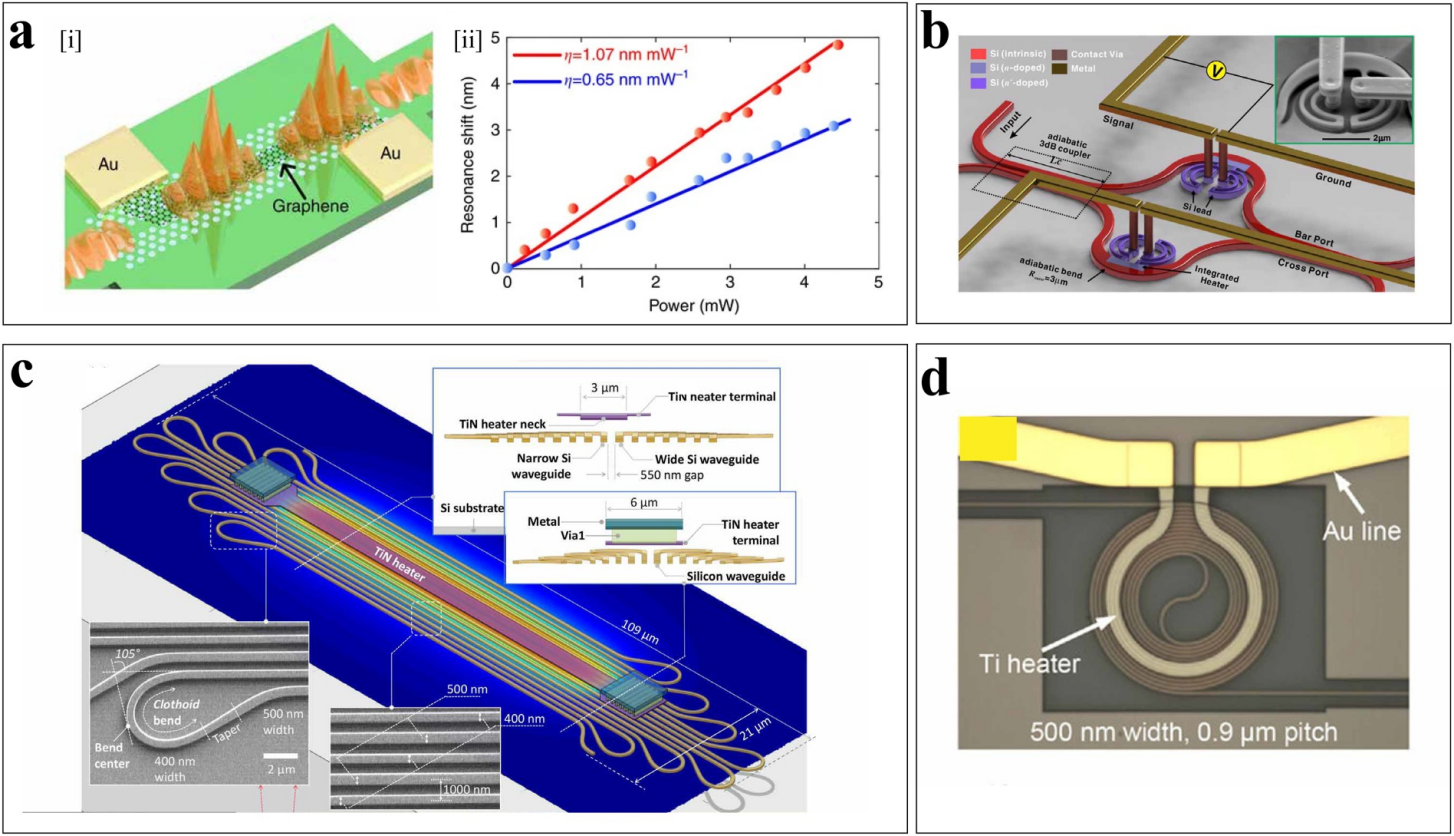
Representative thermo‐optic phase‐shifter designs. (a) [i] Slow‐light‐enhanced thermo‐optic phase shifter based on a graphene microheater integrated with a silicon‐photonic crystal waveguide. [ii] Direct graphene–waveguide contact improves heat transfer, whereas the slow‐light effect enhances the effective thermo‐optic response, enabling a tuning efficiency of up to 1.07 nm ${\text{mW}}^{-1}$ in the slow‐light regime [103]. (b) Thermo‐optic phase shifter based on an adiabatic silicon waveguide with an embedded resistive heater. By integrating the heater into the widened waveguide section through an adiabatic bend, the device reduces thermal mass and improves heating efficiency within a compact footprint [104]. (c) Geometrically optimized low‐power thermo‐optic phase shifter based on a folded silicon waveguide and densely arranged peripheral waveguides. This layout improves the utilization of heat generated by the TiN heater, enabling efficient phase tuning in a compact footprint while preserving compatibility with standard silicon‐photonic processes without additional undercut or trench isolation steps [100]. (d) Compact round‐spiral thermo‐optic phase shifter. A Ti heater is positioned above the central region of the densely packed spiral waveguide to maximize heat utilization, enabling compact, low‐loss, and energy‐efficient thermo‐optic phase tuning [81].

Another important route is to directly heat the waveguide core rather than relying on overcladding heaters. In this context, an adiabatic silicon waveguide structure with an embedded resistive heater was developed, as shown in Figure 8b, where the adiabatic bend provides electrical access while preserving low optical loss. This design achieved 12.7 mW/π power consumption within a footprint of 6μm×6μm [104]. By minimizing the thermal mass of the active region, this architecture substantially improves heating efficiency and supports fast, compact, and low‐power thermo‐optic phase control.

Geometrical optimization of the heated waveguide region has provided another effective route to improving TO efficiency. In folded silicon waveguide MZI switches, a 50:50 adiabatic splitter can be used for balanced splitting and recombination, whereas multiple waveguide folds beneath the heater enhance the thermo‐optic interaction length. Such architectures have demonstrated power consumption as low as 3.8 mW/π, albeit at the expense of a relatively large footprint of 800μm×180μm [105]. Here, the adiabatic splitter serves as the 50:50 power splitter/combiner of the MZI, providing balanced optical distribution for interference‐based switching. To improve efficiency without sacrificing fabrication scalability, geometrical design optimization has also emerged as an effective alternative to suspended or undercut structures. A representative example is shown in Figure 8c, where a folded silicon waveguide layout with densely arranged peripheral waveguides is combined with a TiN heater to recover heat that would otherwise be dissipated away from the active region [100]. By enhancing heat utilization through spatial waveguide design rather than aggressive thermal isolation, this design achieved a balanced performance in terms of power consumption (2.56 mW/π), modulation speed (10.1 kHz), insertion loss (1.23 dB), and footprint (109μm×21μm), albeit with increased structural complexity compared to conventional straight or folded heaters [100].

Additional efficiency enhancements rely on thermal isolation techniques, such as the incorporation of air trenches and selective substrate undercutting around the phase‐shifting region. By exploiting the significantly lower thermal conductivity of ${\text{SiO}}_{2}$ and air compared to silicon, parasitic heat dissipation can be substantially suppressed [105, 106, 107]. Although such approaches effectively reduce the required phase‐shifting power, they introduce additional fabrication complexity.

Recent work has also explored spiral‐based thermo‐optic phase shifters as an effective route to improving the balance among power consumption, modulation speed, insertion loss, and device footprint. Early demonstrations included racetrack‐spiral configurations, whereas subsequent work developed a more fabrication‐friendly round‐spiral design compatible with deep‐ultraviolet (DUV) lithography [81, 108]. In the round‐spiral structure, a compact silicon spiral waveguide is combined with a centrally positioned Ti heater (Figure 8d) so that residual heat generated by the heater can be efficiently reabsorbed by the densely packed waveguide turns. This codesign of heater placement and waveguide geometry improves thermo‐optic efficiency while preserving a compact footprint and low optical loss, making it particularly attractive for large‐scale photonic integrated circuits that require a large number of phase shifters. The optimized round‐spiral device achieved a power consumption of 3.1 mW/π, a modulation bandwidth of 34 kHz, an insertion loss of 0.6 dB, and a compact footprint of $42\times 42\;\mu m^{2}$ [108]. Its applicability was further validated in OPA systems, where it supported two‐dimensional beam steering with low per‐channel power consumption and low overall electrical dissipation during operation [81, 108, 109].

Taken together, these advances show that substantial improvements in TO phase‐shifter efficiency can be achieved through resonant enhancement, heater and material engineering, geometrical heat management, and thermal isolation. However, the reported metrics are not always directly comparable because they are extracted from different device configurations and phase‐shifting mechanisms. More broadly, TO phase shifters still exhibit inherent trade‐offs among power consumption, speed, loss, and footprint, reflecting the fundamental constraints imposed by thermal transport processes [109, 110, 111]. Despite these advances, the inherent power consumption of TO phase shifters, typically in the range of 10–50 mW/π [104, 112], remains a major bottleneck for large‐scale OPA implementations.

#### 4.2.2 Electro‐Optic Phase Shifters in Silicon

In contrast to TO modulation, electro‐optic (EO) phase shifters enable refractive‐index control through electric‐field‐induced mechanisms, offering much higher modulation speeds and substantially lower power consumption. In silicon photonics, EO phase modulation is predominantly realized through the plasma‐dispersion effect, typically using PIN junctions operated in either forward‐ or reverse‐biased configurations.

Forward‐biased PIN phase shifters rely on carrier injection into the intrinsic region, producing relatively large refractive‐index changes but at the cost of significant free‐carrier absorption, pronounced thermal effects, milliwatt‐level static power consumption, and limited modulation bandwidth [89, 113, 114, 115]. By contrast, reverse‐biased PIN phase shifters operate via carrier depletion, enabling much higher modulation speeds with nearly zero static power consumption and significantly reduced thermal crosstalk. Although depletion‐based devices generally exhibit lower phase‐shifting efficiency than carrier‐injection counterparts, recent demonstrations have achieved effective tuning efficiencies down to the sub‐μW/π level together with modulation bandwidths reaching hundreds of megahertz [27, 116]. These characteristics make carrier‐depletion modulators particularly attractive for large‐scale, low‐power, and high‐frame‐rate OPA systems [117].

From a device‐implementation perspective, these performance trade‐offs are closely related to the junction geometry and carrier modulation mechanism adopted in silicon PIN phase shifters. Carrier‐injection EO modulators typically employ PIN‐diode rib‐waveguide geometries, in which p‐ and n‐doped regions are introduced on the two lateral sides of the waveguide (Figure 9a). Under forward bias, injected carriers modify the silicon refractive index and thereby induce an additional optical phase delay relative to the unactuated state. Such a scheme can support nanosecond‐scale phase tuning and fast beam steering and generally offers lower power consumption than TO phase shifters. However, these advantages are accompanied by increased design complexity, doping‐induced propagation loss, variable insertion loss, and non‐negligible drive current requirements, with some reported devices requiring currents of about 10 mA/π [39, 40, 41]. By comparison, depletion‐mode devices are usually optimized toward higher speed, lower static power consumption, and reduced thermal crosstalk, making them more favorable for scalable OPA implementations.

**FIGURE 9:**
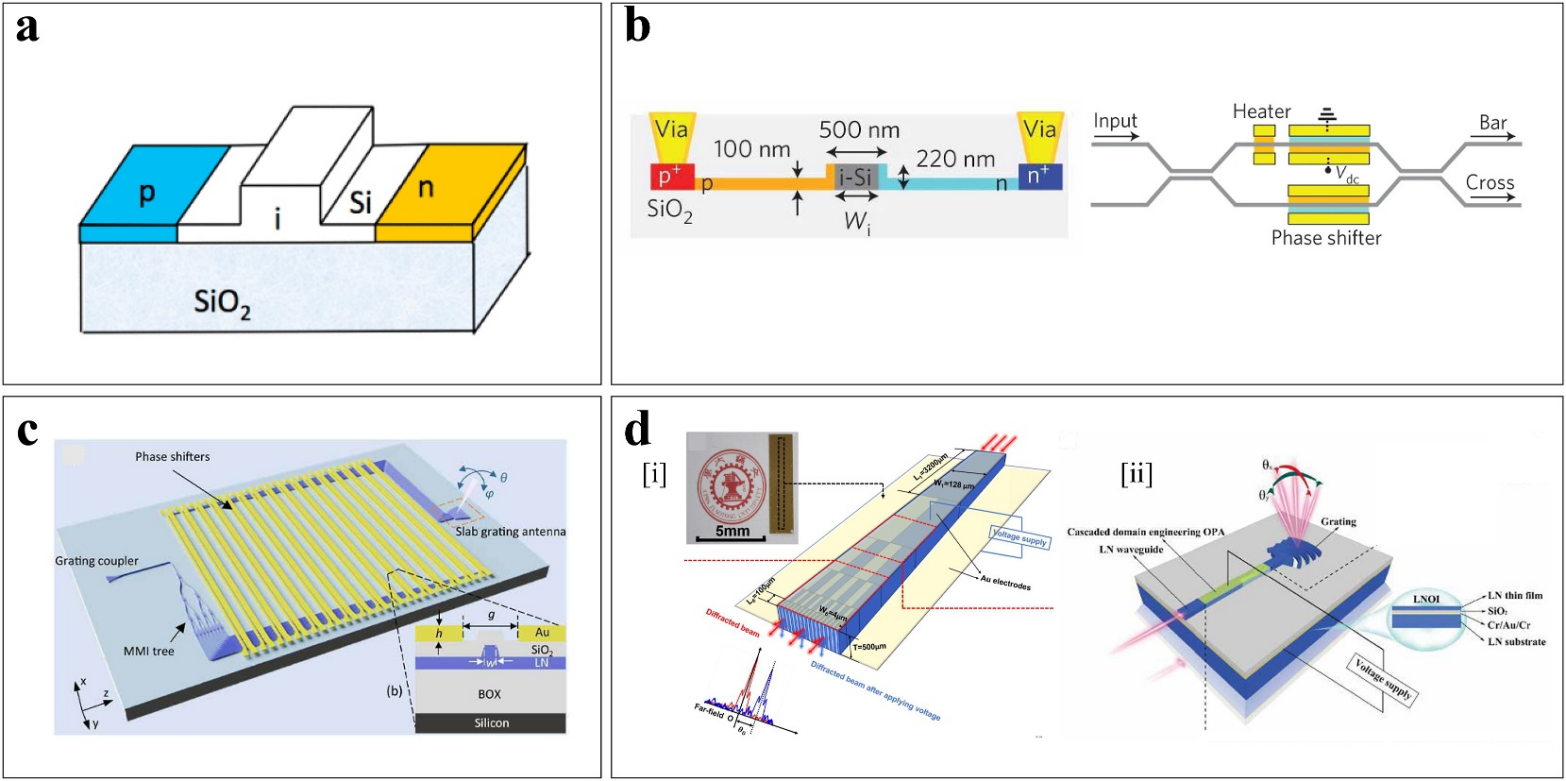
The electro‐optic modulators. (a) EO phase shifter with silicon rib waveguide and doped (p and n) waveguide sides [28]. (b) Field‐induced electro‐optic phase shifting in silicon based on the DC Kerr effect. The figure illustrates the cross‐sectional p‐i‐n ridge‐waveguide geometry, the modal profile, the Mach–Zehnder interferometer configuration, and the measured phase‐shifting characteristics. By applying a reverse bias across the lateral p‐i‐n junction, a strong transverse DC electric field is established in the silicon core, inducing an effective second‐order nonlinear response from the intrinsic third‐order susceptibility of silicon [118]. (c) Schematic of a high‐speed thin‐film lithium niobate optical phased array (OPA). Input light is coupled into the chip through a grating coupler, divided by a cascaded MMI splitter tree, and routed into a traveling‐wave electro‐optic phase‐shifter array implemented on the TFLN platform. After independent phase modulation in each channel, the optical fields are emitted through an aperiodic waveguide array and a slab grating antenna to realize two‐dimensional beam steering [44]. (d) Cascaded‐domain‐engineering optical phased array with a single EO phase shifter [46, 119].

To mitigate the intrinsic limitations of plasma‐dispersion‐based silicon EO phase shifters, recent advances in heterogeneous integration, including silicon–lithium niobate (Si–LN) platforms, have provided alternative solutions that alleviate loss, thermal sensitivity, and drive‐power constraints while maintaining comparable modulation efficiencies [120].

#### 4.2.3 Emerging Field‐Induced Electro‐Optic Mechanisms in Silicon

Beyond conventional carrier‐injection and carrier‐depletion phase shifters based on the plasma‐dispersion effect, recent efforts have explored field‐induced electro‐optic mechanisms in silicon as an alternative route toward phase‐dominant modulation with reduced chirp and potentially lower excess loss. In these approaches, a strong transverse DC electric field is established across the silicon waveguide, typically using a reverse‐biased p‐i‐n junction geometry. Rather than relying primarily on free‐carrier modulation, the applied DC field interacts with the intrinsic third‐order nonlinearity of silicon to induce a field‐dependent refractive‐index perturbation. In an equivalent description, this mechanism can also be viewed as effective‐$\chi^{\left(2\right)}$ engineering in centrosymmetric silicon, thereby linking phase shifting with broader electric‐field‐induced nonlinear optical functionalities.

A representative implementation is shown in Figure 9b, where the cross‐sectional p‐i‐n ridge‐waveguide geometry and Mach–Zehnder interferometer configuration are presented together [118]. In this device architecture, the lateral p‐i‐n junction is primarily used to establish a strong DC electric field across the silicon core, thereby enabling field‐induced phase modulation rather than conventional carrier‐dispersion‐dominated operation. This distinction is important because it provides a route toward predominantly phase modulation with reduced amplitude chirp.

Timurdogan et al. provided a seminal demonstration of this concept by realizing DC‐Kerr‐based phase shifting in silicon Mach–Zehnder interferometers, with a reported $V_{\pi }L$ as low as 2.8V⋅cm [118]. Compared with conventional plasma‐dispersion phase shifters, this field‐induced mechanism offers the prospect of lower chirp and reduced modulation‐induced optical penalty. The same study also demonstrated quasi‐phase‐matched electric‐field‐induced second‐harmonic generation, underscoring that this platform is not only relevant to phase shifting but also to broader effective‐$\chi^{\left(2\right)}$ functionalities in CMOS‐compatible silicon photonics.

An especially interesting extension of this concept is its applicability under cryogenic conditions, where thermo‐optic tuning becomes strongly suppressed and conventional carrier‐based schemes may face additional constraints, such as carrier freeze‐out and excess loss. In this context, Chakraborty et al. demonstrated DC‐Kerr‐effect‐based silicon modulators operating at 5 K with GHz‐class bandwidth [121]. Their results showed that effective refractive‐index shifts on the order of $10^{-4}$ could still be achieved at cryogenic temperatures, highlighting the potential of this mechanism for cryogenic silicon photonics, including quantum photonic circuits and low‐temperature optical interconnects.

Taken together, these studies establish field‐induced electro‐optic modulation based on effective‐$\chi^{\left(2\right)}$ engineering and the DC Kerr effect as a promising emerging direction for silicon phase shifters. Although still at an early stage compared with mature free‐carrier‐based devices, this class of mechanisms opens a new pathway toward low‐chirp and potentially low‐loss phase modulation in a fully CMOS‐compatible silicon platform, with promising prospects for high‐speed operation and cryogenic compatibility.

#### 4.2.4 Electro‐Optic Phase Shifters Based on Lithium Niobate

Lithium niobate (${\text{LiNbO}}_{3}$) has emerged as one of the most promising platforms for integrated electro‐optic (EO) phase shifters because of its strong Pockels effect, excellent linearity, low optical loss, and improving compatibility with large‐scale photonic integration [120]. In the context of on‐chip OPAs, these attributes are particularly attractive because they enable high‐speed, pure phase modulation with much lower energy consumption than thermo‐optic tuning.

Early progress in monolithic LNOI integration demonstrated OPAs with steering ranges of 23°×8° and energy efficiencies of 13.5 pJ/π [45]. Subsequent scalable 48‐channel LNOI OPAs further expanded the field of view to 40°×8.8°, while achieving beam divergences as low as 0.33°×1.8°, phase‐shift energies on the order of 1.11 nJ/π, and modulation bandwidths up to 70 MHz [42]. Heterogeneous LNOI–SiN platforms have extended the performance envelope even further, enabling operation speeds of 1.2 GHz with 3.2 pJ/π efficiency and steering ranges of 50°×8.6° [43]. These advances establish LNOI‐based EO phase shifters as a leading solution for high‐speed and energy‐efficient beam steering on chip.

A representative TFLN‐based OPA architecture is schematically illustrated in Figure 9c [44]. In this design, the input light is first coupled into the chip through a grating coupler and then equally distributed into 16 channels by a four‐stage cascaded MMI splitter tree. Each channel is subsequently routed through a traveling‐wave phase shifter implemented on the TFLN platform, enabling high‐speed and pure phase modulation based on the Pockels effect. After phase tuning, the optical signals are fed into an aperiodic waveguide array combined with a slab grating antenna, where beam steering in one direction is achieved through electro‐optic phase control and scanning in the orthogonal direction is realized through wavelength tuning. This architecture illustrates how TFLN phase shifters can be integrated with broadband driving electrodes and large‐field‐of‐view emission structures to support high‐speed two‐dimensional beam steering.

Traveling‐wave electrode designs have further pushed EO bandwidths to 2.5 GHz while maintaining wide fields of view (50°×8.6°) at 330 pJ/π energy consumption [44]. Taken together, these results show that LNOI‐based EO phase shifters can simultaneously provide subnanosecond response, low phase‐shift energy, and wide‐angle beam steering, which remain difficult to achieve simultaneously on conventional silicon‐photonic platforms.

Beyond serving as high‐speed tuning elements in conventionally addressed OPAs, LN electro‐optic phase shifters can also enable structurally encoded phase control through ferroelectric‐domain engineering. In this scheme, the phase‐generation function is embedded directly into the ferroelectric‐domain architecture, thereby reducing or eliminating the need for per‐element independent electrical addressing. As illustrated in Figure 9d [46, 119], this concept was first demonstrated in bulk ${\text{LiNbO}}_{3}$, where a six‐layer cascaded‐domain‐engineering structure enabled continuously tunable beam steering with 32 effective array elements at speeds up to 3 MHz under single‐electrode control. The same principle was later translated to the LNOI platform and integrated with an on‐chip grating antenna to realize single‐electrode two‐dimensional beam steering, with simulated scanning ranges of 42°×9.2°. This direction suggests that LN‐based phase shifters are not only advantageous as ultrafast tuning elements but also uniquely suited to low‐complexity beam‐steering architectures in which phase control is partially or fully encoded into the material structure itself.

A comprehensive comparison of the key performance metrics of TO phase shifters is presented in Table 1, whereas a corresponding comparison for EO phase shifters is provided in Table 2. Although TO devices retain advantages in fabrication maturity and cost, EO modulators—particularly those based on LNOI—exhibit superior potential in energy efficiency and modulation speed, making them well suited for high‐performance OPA systems requiring fast response and low power consumption. This fundamental trade‐off between manufacturability and performance continues to guide phase‐shifter selection across diverse photonic integration scenarios.

**TABLE 1:** Performance comparison of examples from different thermo‐optic phase shifters.

Ref	*P*_π_	Voltage (2π)	Modulation speed	Loss (dB)	Footprint (μm ×μm)
[122]	22.3 mW	6 V	NA	NA	NA
[123]	22 mW	NA	50 kHz	NA	NA
[81]	3.1 mW	1.1 V	34 kHz	0.6 dB	42×42
[124]	13.54 mW	NA	17 kHz	7.87 dB	NA
[125]	22.3 mW (TE); 35.1 mW (TM)	NA	NA	3×100	NA
[126]	18.7 mW	NA	NA	NA	5×200
[127]	6.5 mW	NA	NA	< 0.1 dB	2×125
[128]	6 mW	NA	NA	NA	5×125
[129]	42.5 mW	NA	NA	2.3 dB	50×10
[26]	13 mW	12 V	NA	NA	NA
[27]	2 μW	NA	33.3 kHz	2.4 dB	NA
[130]	300 μW	NA	330 kHz	0.5 dB	NA
[101]	50 mW	NA	100 kHz	3.1 dB	1200×14
[110]	6.5 mW	NA	25 kHz	3.7 dB	130×130
[104]	12.7 mW	NA	146 kHz	1.7 dB	6×6
[131]	1.7 mW	NA	53 kHz	4.6 dB	850×25
[132]	13.6 mW	NA	186 kHz	0.78 dB	100×2

*Note:* NA indicates data not available in the referenced literature.

Abbreviations: TE, transverse electric; TM, transverse magnetic.

**TABLE 2:** Performance comparison of examples from different electro‐optic phase shifters.

Ref	Principle	*P*_π_	Voltage (2π)	Modulation speed	Loss (dB)	Footprint (μm ×μm)
[42]	Pockels EO effect	1.11 nJ	7.5 V	70 MHz	NA	4.5×8000
[43]	Pockels EO effect	3.2 pJ	3.64 V	1.2 GHz	NA	15×15600
[45]	Pockels EO effect	13.5 pJ	6 V	4.35 GHz	−0.5 dB/cm	20×10000
[44]	Pockels EO effect	0.33 nJ	6.42 V	2.5 GHz	0.0001 dB/cm	5×10000
[133]	Pockels EO effect	NA	4.85 V	66.7 MHz	NA	5×5000
[113]	p‐i‐n junction EO effect	1.7 mW	1.1 V	20 MHz	3.2 dB	2.5×1000
[27]	p‐i‐n junction EO effect	2 μW/2π	NA	NA	3.2 dB	NA
[70]	p‐n junction EO effect	1.8 μW/2π	NA	30 μs	7.45 dB	7000‐long
[134]	p‐i‐n junction EO effect	48 mW/2π	NA	NA	2.1 dB/cm	4×800

*Note:* NA indicates data not available in the referenced literature.

### 4.3 Grating‐Coupler Design

Optical antennas are fundamental components of integrated optical phased arrays (OPAs), serving as the interfaces that convert guided optical modes into free‐space radiation and, in some architectures, also support optical reception [28, 144]. In current OPA implementations, two major emission schemes are commonly adopted: edge‐emitting configurations and grating‐based emitters. Although edge‐emitting OPAs mainly support one‐dimensional beam steering through phase modulation between adjacent elements, grating‐based emitters enable full two‐dimensional beam formation and scanning, which is essential for high‐resolution three‐dimensional sensing and imaging applications. This subsection, therefore, focuses on grating‐emitter design and its impact on OPA performance.

The performance of OPAs strongly depends on the architecture of the grating emitter, which influences not only radiation efficiency but also emission directionality, aperture utilization, beam quality, and ultimately the achievable steering range. In general, high‐performance grating emitters for OPAs require three key characteristics: high upward radiation efficiency, sufficiently long effective emission length, and a well‐controlled radiation envelope. From an architectural perspective, grating emitters for OPAs can be broadly classified into three main categories: sidewall‐modulated gratings [135, 145], shallow/deep‐etched gratings, and subwavelength gratings (SWGs) [76, 142, 146].

Sidewall‐modulated gratings employ periodic waveguide‐width perturbations to radiate the guided ${\text{TE}}_{0}$ mode while maintaining relatively weak structural modulation. In these structures, width‐induced optical path differences can support quasi‐bound states in the continuum (qBIC), which suppress lateral leakage through destructive intrawaveguide interference and thereby enable very weak and controllable radiation [47]. This mechanism is particularly attractive for OPA emitters because it helps extend the effective radiation length and reduce far‐field beam divergence. Representative SOI implementations have demonstrated 128‐element arrays with unit lengths of 1.3 mm and theoretical far‐field divergence angles as low as 0.05° [47], whereas related triangular, fully etched nanogroove designs further illustrate the flexibility of this concept, as shown in Figure 10a [123]. In this architecture, the grating antenna is formed by a periodically corrugated waveguide, where the radiation strength can be tailored through structural parameters such as the grating‐waveguide width. Unlike conventional weakly etched gratings, which often require extremely small corrugations and may still suffer from undesired lateral leakage, the quasi‐BIC mechanism suppresses sidewall emission by introducing destructive intrawaveguide interference, thereby enabling a pronounced reduction in diffraction strength. Numerical results further show that the diffraction strength α varies strongly with the waveguide width $W_{\text{grt}}$ at 1550 nm and reaches a minimum near the quasi‐BIC condition, indicating a regime of strongly suppressed radiation [47].

**FIGURE 10:**
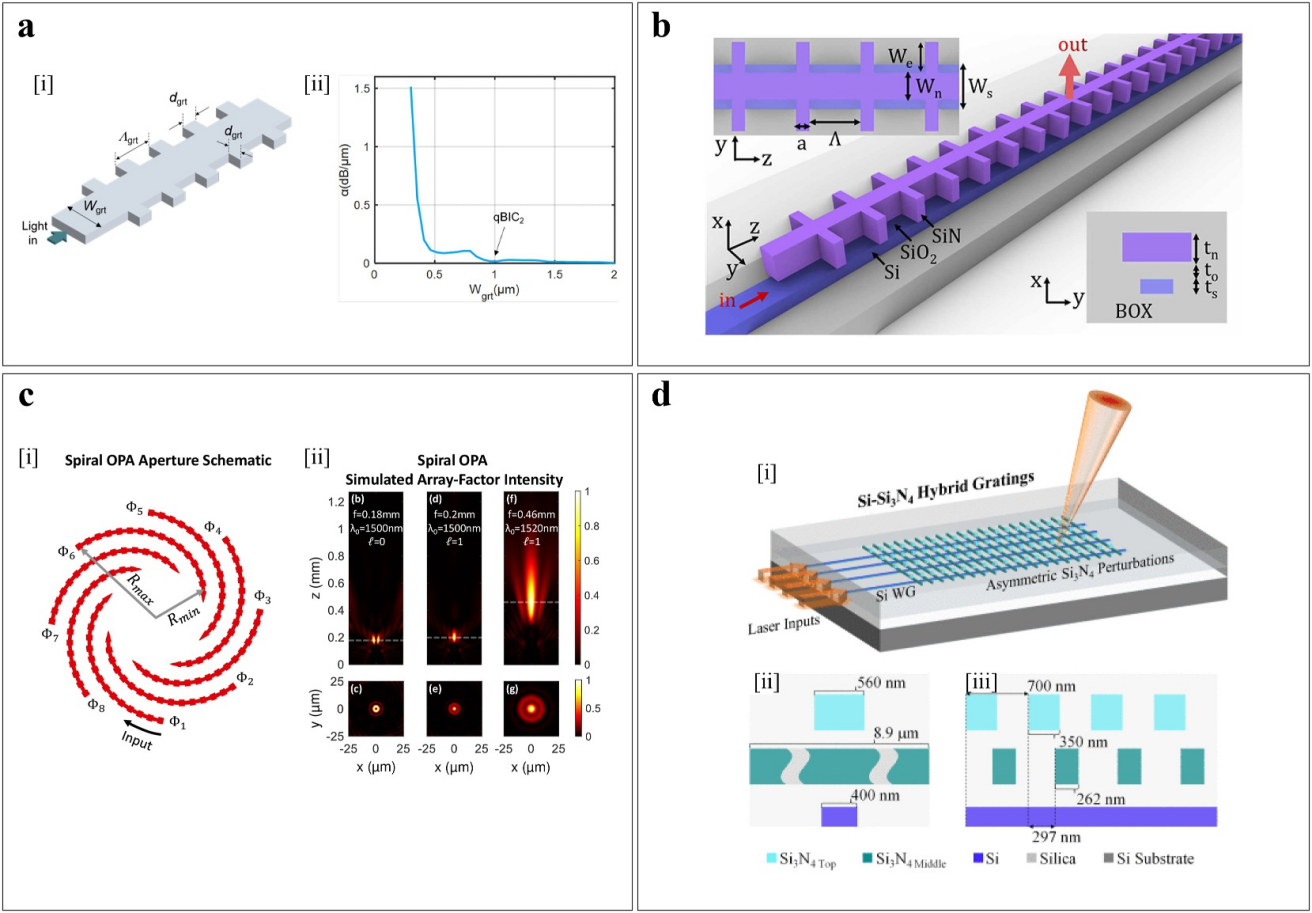
Representative grating‐emitter architectures and functionalities. (a) Quasi‐BIC‐based grating antenna for diffraction‐strength engineering. [i] Schematic of the quasi‐BIC grating antenna, showing the periodically corrugated waveguide structure and key geometric parameters. [ii] Numerically calculated diffraction strength α versus waveguide width $W_{\text{grt}}$ at 1550 nm shows a pronounced minimum near the quasi‐BIC condition [47]. (b) Vertical directional‐coupling waveguide grating (VDCWG) for weak‐emission engineering in optical phased arrays. The structure consists of a lower silicon strip waveguide, an intermediate ${\text{SiO}}_{2}$ spacer, and an upper silicon nitride fishbone grating waveguide. Optical power is gradually transferred from the silicon waveguide to the upper SiN grating waveguide through vertical directional coupling, enabling controlled free‐space radiation; the inset shows the top and side views of the multilayer geometry [140]. (c) Spiral grating‐emitter aperture for near‐field‐focusing optical phased arrays. [i] Top‐view schematic of a spiral integrated OPA aperture formed by azimuthally arranged spiral grating‐based antennas, together with [ii] simulated field distributions in the x‐z plane and x‐y focal plane, illustrating near‐field focusing with wavelength‐tunable focal height and phase‐dependent focal‐plane profiles [147]. (d) Hybrid Si/${Si}_{3}N_{4}$ grating‐emitter aperture for a large‐scale optical phased array. Schematic of the emitting aperture, together with the corresponding [i] three‐dimensional, [ii] cross‐sectional, and [iii] side views of the grating‐emitter structure. Light is guided in the silicon waveguide and radiated through periodic asymmetric ${\text{Si}}_{3}N_{4}$ perturbations, forming a long and weakly radiating aperture for narrow‐divergence beam emission [76].

Conventional silicon grating emitters are often limited by the high Si/${SiO}_{2}$ index contrast, which tends to produce short effective radiation lengths and exponentially decaying near‐field envelopes, thereby constraining far‐field beamwidth reduction [138]. To address this issue, apodization techniques have been introduced to tailor the local diffraction strength and flatten the emission profile. Advanced implementations, including SiN‐overlaid gratings and bilayer SiN gratings, have achieved substantially improved beamwidth performance, although sometimes at the expense of MPW compatibility [143, 148, 149]. In a related design direction, a dual‐layer ${\text{Si}}_{3}N_{4}$ grating antenna formed by vertically stacking two identically patterned emitters was shown to reshape the effective refractive‐index distribution and enhance upward emission directionality to above 92% [74].

Shallow/deep‐etched grating antennas realize optical radiation through alternating etch depths, periods, and duty cycles, providing an effective compromise between diffraction efficiency and fabrication practicality. Deep transverse etches in straight‐waveguide configurations can simplify processing and yield near‐vertical sidewalls, with reported upward emission efficiencies of about 60% in footprints of 3.76 μm × 1.5 μm [48]. Monolithic flat gratings matched to the waveguide etch depth eliminate secondary etching steps and reduce interwaveguide crosstalk, enabling dense array pitches down to 1.5 μm [42, 44, 45]. Additional performance enhancement can be achieved by combining transverse stitched gratings with Bragg mirrors and bottom reflectors, leading to upward diffraction efficiencies as high as 88% at 1550 nm [136]. In general, these designs improve performance through three main routes: increasing tooth height to enhance diffraction, introducing shallow‐etched auxiliary regions to boost upward radiation, and incorporating strip structures to suppress back‐reflection losses [141, 150, 151].

Subwavelength and metamaterial‐assisted grating architectures provide an important alternative route to weak‐emission engineering by enabling precise control of effective refractive‐index perturbation, modal delocalization, and radiation strength through engineered subwavelength structures and hybrid waveguide configurations [76, 90, 142, 146, 152, 153]. Compared with conventional waveguides, SWGs can exhibit reduced propagation loss and enhanced evanescent fields, whereas their effective refractive index can be engineered through the period, duty cycle, and material configuration. Beyond conventional sidewall‐modulated emitters, metamaterial engineering has opened an important alternative route toward long and weakly radiating surface emitters in silicon. Ginel‐Moreno et al. introduced a surface‐emitting antenna based on an SWG metamaterial waveguide core evanescently coupled to laterally separated radiative segments, replacing ultraweak sidewall corrugations with a more controllable overlap‐engineering strategy for radiation‐strength tuning. This approach enabled a 3.65‐mm‐long antenna with an ultranarrow beam divergence of 0.025°, whereas L‐shaped radiative segments further increased the radiation efficiency to 72% [154]. The concept was subsequently translated into a more fabrication‐compatible implementation on a standard 220‐nm SOI platform, where a 2‐mm‐long single‐etched antenna with an 80‐nm minimum feature size achieved a measured divergence of 0.1° and a wavelength‐tuning sensitivity of 0.13°/nm, demonstrating that metamaterial surface‐emitting antennas can be realized without relying on sub‐10‐nm sidewall perturbations [155]. More recently, this antenna platform was further extended to a system‐level OPA architecture, in which 112‐mm‐long metamaterial antennas were illuminated by a distributed Bragg deflector instead of a conventional beam‐splitting tree, enabling compact feeding, intrinsic Gaussian apodization, and wavelength‐controlled two‐dimensional beam steering [156]. Together, these studies establish a clear development path from weak‐radiation antenna physics to manufacturable SOI implementations and ultimately to compact OPA system integration.

To overcome the limited effective emission length of conventional silicon waveguide grating antennas, recent work has introduced multilayer grating‐emitter architectures that engineer radiation through vertical mode coupling rather than relying solely on weakening the grating perturbation itself. As shown in Figure 10b, the vertical directional‐coupling waveguide grating (VDCWG) employs a three‐layer Si–${\text{SiO}}_{2}$–SiN structure, in which the input light first propagates in a silicon strip waveguide and is then gradually transferred to an upper silicon nitride fishbone grating waveguide through vertical directional coupling [140]. In this scheme, the SiN grating layer functions not merely as a passive perturbation layer but as a radiating waveguide that both supports guided propagation and emits light into free space [140]. The emission strength is jointly determined by the grating radiation loss, the propagation‐constant mismatch, and the interlayer coupling coefficient, which significantly expands the design freedom for weak‐emission engineering and enables substantially longer antenna lengths than in conventional single‐layer gratings.

A related silicon/silicon nitride hybrid grating‐emitter architecture, shown in Figure 10d, was developed on a multilayer ${\text{Si}}_{3}N_{4}$‐on‐Si platform, where each array element consists of a silicon waveguide vertically coupled to asymmetric ${\text{Si}}_{3}N_{4}$ perturbation strips [76]. In this design, the guided light in the silicon waveguide experiences periodic refractive‐index perturbations introduced by the vertically separated ${\text{Si}}_{3}N_{4}$ structures, enabling gradual out‐of‐plane radiation with a very small coupling strength per period. This weak‐emission mechanism allows the grating antenna to extend over a centimeter‐scale effective length, thereby significantly reducing the beam divergence in the wavelength‐scanning direction. The asymmetric arrangement of the ${\text{Si}}_{3}N_{4}$ perturbations further breaks the vertical symmetry and promotes upward emission [76]. Such a hybrid grating strategy illustrates an important direction in grating‐emitter design for OPAs: Rather than relying only on shallow etching or local perturbation minimization, the radiation strength and beam quality can be coengineered through multilayer index perturbations and vertically hybridized waveguide‐grating structures.

Beyond the basic architectural classification, recent work has shown that grating‐emitter design can also be exploited to realize advanced functionalities beyond simple far‐field outcoupling. These advanced functionalities mainly fall into five directions: radiation‐envelope engineering, steering‐range extension, near‐field focusing, active radiator‐based beam control, and residual‐light reuse for on‐chip monitoring and diagnostics.

Radiation‐envelope engineering has been explored as an effective means to improve aperture utilization and beam quality while remaining compatible with practical fabrication constraints. In a representative ${\text{Si}}_{3}N_{4}$ visible‐wavelength grating antenna operating at 532 nm, a side waveguide inserted between the bus waveguide and the grating teeth enabled weak and gradually tailored coupling, thereby producing a more uniform emission profile and a substantially expanded excited aperture [157]. Effective aperture expansion has also been demonstrated through large‐scale multichannel integration and chirped grating antennas, yielding beam divergences as low as 0.01° in 8192‐element arrays [29] and 0.05°×0.04° in coherent ranging systems with 100 m detection capability [72].

More complex grating‐emitter geometries have also been introduced to directly shape the emitted wavefront. As illustrated in Figure 10c, a spiral integrated OPA can be formed by azimuthally arranging multiple spiral grating antennas into a circular aperture, thereby enabling near‐field focusing emission with wavelength‐tunable focal height [147]. In this architecture, each spiral antenna maps the longitudinal phase accumulation of a grating waveguide onto a curved Archimedean‐spiral path so that the emitted field acquires an effective radially varying phase curvature. As a result, the grating‐emitter geometry itself contributes directly to focusing‐wavefront formation, rather than serving only as a conventional outcoupling element [147].

Grating‐emitter engineering has further been used to enlarge steering range. Guo et al. proposed a bidirectional wide‐angle waveguide grating antenna that intentionally exploits both upward and downward radiation, rather than suppressing the latter as in conventional designs [158]. Through multilayer optimization with ${\text{SiO}}_{2}$/${Si}_{3}N_{4}$ hybrid antireflection layers, the antenna achieved nearly balanced bidirectional radiation and a flat‐top far‐field response, enabling a field of view exceeding 90° in each direction and a total polar field of view greater than 180° [158]. This work shows that tailoring the radiation characteristics of a single antenna element can substantially enlarge the overall steering range without duplicating the full beamforming architecture.

Beyond passive radiation shaping, tunable radiator designs have extended the role of grating emitters from static outcouplers to active beam‐control elements. Yoon et al. demonstrated a 1×64 silicon OPA that combined electro‐optic p‐i‐n phase shifters for transverse steering with thermo‐optic n‐i‐n tunable radiators for longitudinal steering, thereby achieving two‐dimensional beam steering without relying on wavelength‐tunable laser sources [159]. This result indicates that the radiator itself can be engineered as an active emission unit for multidimensional beam manipulation.

At the system level, grating emitters have also been explored as multifunctional interfaces for residual‐light reuse and beam‐state monitoring. Kwon et al. proposed an on‐chip metalens‐based beamformer in which the residual optical field remaining after grating emission was routed into a planar diffraction region and focused by an on‐chip metalens formed by a one‐dimensional array of ${\text{SiO}}_{2}$ nanoslots [160, 161]. By mapping the residual beam onto multiple monitoring ports associated with different interchannel phase differences, the device enabled compact on‐chip beam‐state monitoring and wavefront mapping [160, 161]. This work highlights the potential of grating emitters to support residual‐light reuse, phase diagnostics, and system‐level functional integration within OPA platforms.

Taken together, the development of grating emitters in on‐chip OPAs has evolved from a narrow emphasis on upward radiation efficiency toward a broader design framework that also includes weak‐emission engineering, aperture‐envelope control, steering‐range extension, active radiator functionality, and system‐level monitoring integration. From an architectural standpoint, shallow/deep‐etched gratings offer high efficiency and fabrication practicality, sidewall‐modulated gratings are attractive for long‐aperture weak‐emission designs, and SWG/hybrid multilayer emitters provide expanded freedom for radiation‐strength and beam‐quality coengineering. Ultimately, optimal grating‐emitter design for OPAs must simultaneously balance upward radiation efficiency, effective antenna length, and radiation‐envelope uniformity while increasingly accommodating advanced beam‐control and system‐integration functionalities. A comprehensive comparison of the key performance metrics of different grating‐emitter architectures is presented in Table 3.

**TABLE 3:** Comparison of different types of grating couplers.

Grating type	Core materials	Diffraction efficiency	Beam width	Steering efficiency	Footprint (μm)
LC‐AWG [47]	Si	−6.85 dB/mm	0.037°	0.132°/nm	15,000 (long)
LC‐AWG [135]	${\text{Si}}_{3}N_{4}$	N/A	0.007°	N/A	192×10000
VC‐GA [48]	${\text{Si}}_{3}N_{4}$	60%	N/A	Phase tuning	N/A
LC‐AWG [136]	Si	88%	38°–136°	N/A	2.8×1.76
LC‐AWG [137]	${\text{Si}}_{3}N_{4}$	45% (TE) 40% (TM)	0.52° 0.49°	0.26°/nm	120×100
VC‐SGA [42]	${\text{LiNbO}}_{3}$	N/A	1.2°	0.098°/nm	48×100
VC‐AWG [43]	${\text{LiNbO}}_{3}$	N/A	0.2°	0.071°/nm	500×60
LC‐AWG [138]	Si/${Si}_{3}N_{4}$	26%	0.12°	N/A	887 (long)
LC dual‐layer AWG [139]	Si/${Si}_{3}N_{4}$	90.4%	N/A	0.1°/nm	N/A
LC‐AWG [123]	Si	9.3 dB/mm	0.076°	0.133°/nm	15,000 (long)
LC dual‐layer AWG [140]	Si/${Si}_{3}N_{4}$	N/A	0.061°	N/A	15,000 (long)
VC‐GA [141]	Si	90.4%*	1°	N/A	5.2×3.3
VC‐GA [142]	Si	47.8%	44°–52°	N/A	3.4×1.78
VC dual‐layer AWG [76]	${\text{Si}}_{3}N_{4}$	N/A	0.016°	0.162°/nm	103,000 (long)
VC dual‐layer AWG [75]	Si/${Si}_{3}N_{4}$	70.7%	2.72°	0.154°/nm	100 (long)
LC dual‐layer AWG [74]	Si	90.9%*	0.066°–0.068°	0.074°/nm	18000×25000
VC‐AWG [45]	${\text{LiNbO}}_{3}$	36%	0.6°	0.08°/nm	N/A
VC‐SGA [44]	${\text{LiNbO}}_{3}$	N/A	2.8°	0.086°/nm	N/A
VC‐AWG [73]	Si	N/A	0.05°	0.157°/nm	20,000 (long)
LC‐AWG [72]	${\text{Si}}_{3}N_{4}$	22.9%	0.05°	0.063°/nm	31600×20500
LC‐AWG [71]	${\text{Si}}_{3}N_{4}$	N/A	0.03°–0.05°	0.131°/nm	23000×23000
LC dual‐layer AWG [70]	${\text{Si}}_{3}N_{4}$	N/A	0.029°	0.069°/nm	4000×3000
LC dual‐layer AWG [143]	${\text{Si}}_{3}N_{4}$	93%*	N/A	N/A	3000 (long)

*Note:* N/A indicates data not available; * indicates simulation results.

Abbreviations: AWG, array waveguide grating; GA, grating antenna; LC, lateral corrugation; SGA, slab grating antenna; TE, transverse electric; TM, transverse magnetic; VC, vertical corrugation.

## 5 Material Platform for OPAs

### 5.1 Si

Silicon‐photonic platforms offer compelling advantages for on‐chip optical phased arrays (OPAs), owing to both their mature fabrication infrastructure and favorable optical properties. Leveraging compatibility with standard CMOS manufacturing, silicon photonics enables high‐precision waveguide fabrication using conventional lithography while also supporting monolithic integration with functional optoelectronic components such as Ge photodetectors and modulators [163]. This compatibility provides a strong foundation for cost‐effective mass production, dense photonic–electronic integration, and scalable system‐on‐chip OPA implementations.

Silicon also benefits from a high refractive‐index contrast (Δn≈2.5 at 1550 nm) with ${\text{SiO}}_{2}$, which enables strong optical confinement in submicron waveguides, compact device layouts, and efficient on‐chip optical routing. In OPA systems, each emitter must ideally support a full 2π phase‐tuning range to enable arbitrary far‐field beam shaping. Silicon‐photonic integrated circuits (PICs) are particularly attractive in this regard because they support multiple phase‐control mechanisms. The thermo‐optic effect provides robust and fabrication‐friendly phase tuning, whereas the free‐carrier‐dispersion effect enables high‐speed modulation. The coexistence of these complementary tuning schemes allows silicon PICs to address the stringent demands of OPA systems in terms of phase control, integration density, and modulation bandwidth [164].

A major development direction of silicon OPAs has been the realization of dense emitter arrays with low crosstalk and a wide steering range. To mitigate crosstalk and sidelobe issues under subwavelength emitter spacing, researchers have introduced high‐density, low‐crosstalk waveguide‐superlattice architectures, enabling waveguide spacing below λ/2 and improving main‐lobe energy concentration while maintaining both longitudinal and transverse coherence control [152]. In 1D edge‐emitting OPAs, a pitch of 775 nm (λ/2), combined with integrated thermo‐optic heaters, enabled wide‐angle beam steering over 64° at 1.55 μm, with suppressed sidelobes and enhanced main‐lobe sharpness [50]. The serpentine 2D OPA layout, which eliminates the need for directional couplers, substantially increased antenna density and power utilization [165], whereas a λ/2‐spaced trapezoidal grating‐based array enabled a 180° field‐of‐view (FoV) scan with sidelobe suppression better than −19 dB [83]. Further designs incorporating sinusoidal waveguides and intentional phase mismatch yielded low‐crosstalk, low‐loss arrays with emitter spacing in the 615–695‐nm range, enabling 180° aliasing‐free scanning for solid‐state LiDAR systems [166]. Along the same direction of large‐field‐of‐view silicon OPA design, Liu et al. [167] demonstrated a 1000‐channel SOI‐based OPA that achieved grating‐lobe‐free beam steering over a full 180° field of view through a half‐wavelength‐pitch waveguide array and a trapezoidal slab grating. Combined with optimized waveguide routing and passive PWM addressing, this design reduced crosstalk and control complexity, underscoring the scalability of silicon OPAs for large‐aperture, high‐resolution beam steering.

Beyond array density and steering range, substantial effort has also been devoted to reducing power consumption and control complexity in large silicon OPAs. To improve system stability and phase‐control intelligence, silicon‐based sub‐bandgap detectors have been employed for real‐time far‐field feedback and dynamic phase correction [168]. As shown in Figure 11a, a 512‐channel silicon optical phased array was realized by combining a binary splitter tree, a dense parallel array of thermo‐optic phase shifters, and a one‐dimensional long‐grating‐emitter aperture. More importantly, this work addressed one of the central limitations of the Si platform, namely, the prohibitive power consumption of large numbers of thermo‐optic phase shifters, by introducing a multipass phase‐shifting architecture based on mode multiplexing. By allowing light to accumulate phase over multiple passes through the same thermo‐optic section, the phase‐shifter power was significantly reduced while preserving broadband operation and microsecond‐level response. This enabled a large active array with 512 independently controlled elements and 2D beam steering over a 70°×6° field of view at a total phase‐shifter power consumption of only 1.9 W [131]. Additional approaches have aimed at reducing wiring overhead and routing complexity. A hierarchical subarray structure reduced phase‐controller wiring complexity from O($N^{2}$) to O(N), enabling single‐chip integration of 1024 grating antennas with significantly improved fabrication tolerance [129]. To further reduce power consumption and routing complexity, a recent 2D OPA design physically separated the phase shifters from the antenna elements, enabling full 2D beam scanning with only 2N modulators; optimization using particle‐swarm and genetic algorithms led to an 8×8 array achieving 10.5°×10.5° scanning and 18 dB sidelobe suppression [169]. In addition, as shown in Figure 11b, a silicon OPA with a dual‐Gaussian power distribution was proposed to suppress sidelobes simultaneously in both the azimuthal (ϕ) and polar (θ) directions. In this architecture, Gaussian power shaping in the ϕ direction is realized by a Y‐splitter‐assisted cascaded coupling scheme, whereas the Gaussian envelope in the θ direction is enabled by an apodized grating emitter. By departing from uniformly illuminated apertures and introducing tailored amplitude distributions, the design substantially improves the sidelobe suppression ratio in both directions [73].

**FIGURE 11:**
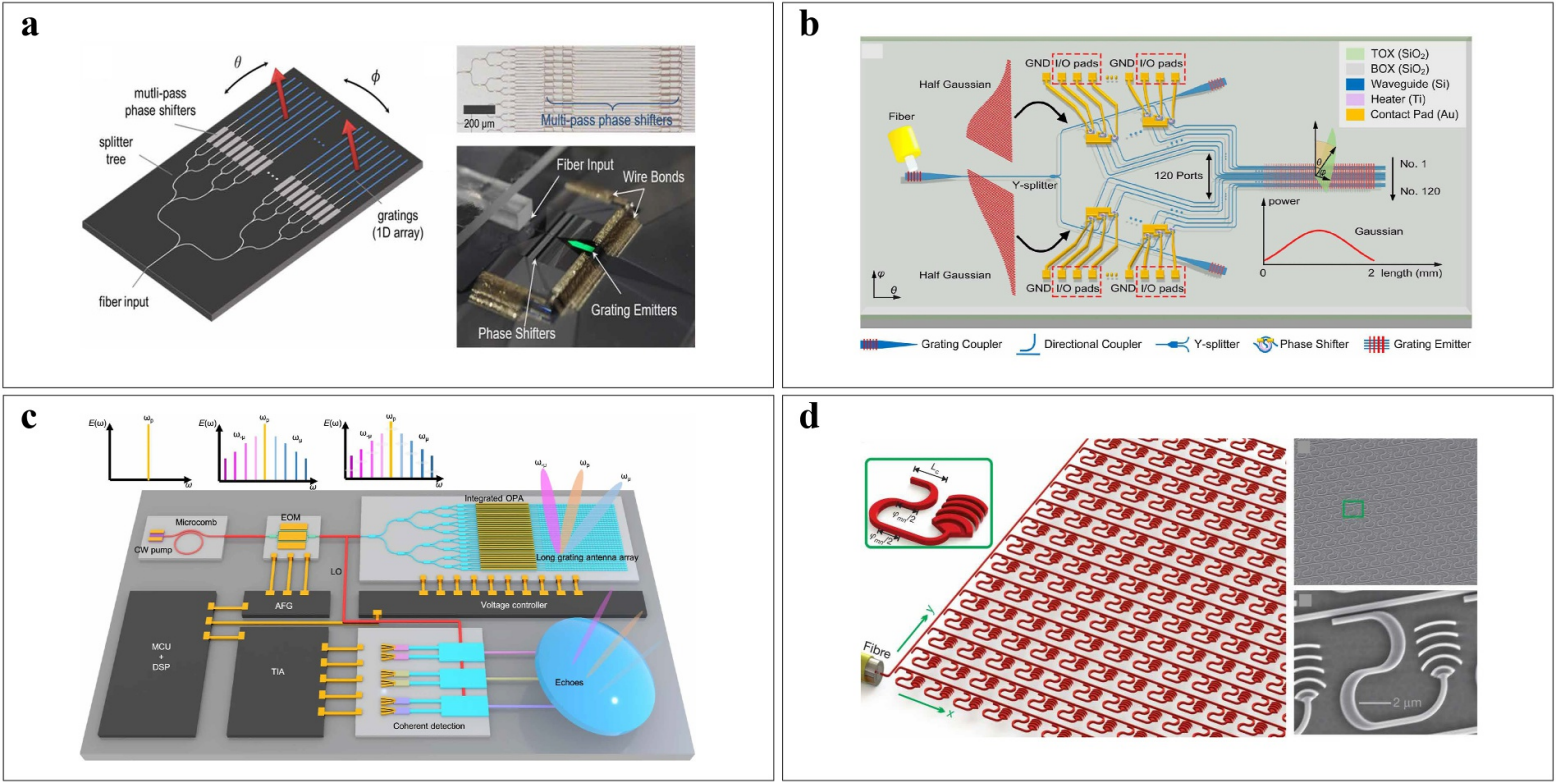
Optical phased array based on the silicon‐photonic platform. (a) Large‐scale silicon optical phased array enabled by a low‐power multipass photonic platform. Schematic of the optical phased array, consisting of a single input waveguide, a binary splitter tree, an array of independently controlled multipass thermo‐optic phase shifters, and a one‐dimensional grating‐emitter aperture for two‐dimensional beam steering. Optical microscope image of the fabricated silicon chip, showing several stages of the splitter tree and part of the multipass phase‐shifter array. Packaged device with the phased‐array chip wire‐bonded to a silicon interposer and coupled to an optical fiber input; the grating‐emission region is highlighted [131]. (b) Dual‐Gaussian‐power distribution silicon optical phased array for bidirectional sidelobe suppression. The designed 12 dB Gaussian power distribution across the 120‐channel OPA reshapes the near‐field amplitude envelope to suppress sidelobes in the far field. By combining this engineered power distribution with complementary grating‐emitter apodization, the silicon OPA achieves simultaneous beam‐quality improvement in both the ϕ and θ directions [73]. (c) The parallel FMCW LiDAR system using an integrated microcomb and optical phased array (OPA). A CW laser pumps the microcomb, whose multiple wavelengths are modulated by an EOM and steered via the OPA. Coherent detection of wavelength‐multiplexed echoes simultaneously retrieves target distance and velocity, supported by AFG, LO, TIA, MCU, and DSP modules [47]. (d) The 64×64 nanophotonic phased‐array system. Laser input from an optical fiber is delivered equally to each of the 4096 nanoantennas through silicon waveguides [162].

Silicon OPAs have also benefited from continued advances in active phase control and beamforming functionality. An 8×8 2D OPA chip incorporating silicon electro‐optic modulators, interferometric calibration, and nanosecond‐level switching demonstrated narrow beams and high‐speed full‐field 2D control [170]. A 2D OPA array based on strongly overcoupled microring phase shifters achieved ultralow power consumption of about 300 μW, high modulation rates of about 330 kHz, and full 2π phase control, establishing a strong foundation for future programmable optical processors and on‐chip spatial light modulators [130]. Hybrid designs such as bent‐waveguide‐plus‐${\text{SiO}}_{2}$‐microcavity architectures effectively suppressed crosstalk and intensity decay associated with λ/2 spacing, enabling large‐angle beam scanning [96]. A 1.3‐μm‐pitch design further enabled broadband, sidelobe‐free scanning, paving the way for future ultralarge‐scale OPA expansion [171]. Beyond array scaling and control optimization, recent SOI‐based OPAs have also explored steering‐range enhancement through platform‐level design. Kim et al. [172] demonstrated a dual‐polarization‐multiplexed silicon OPA on a standard 220‐nm SOI platform, where co‐optimization of the polarization‐handling components and a pixelized grating antenna enabled continuous beam steering across both TE and TM polarizations. By mitigating the effective‐index mismatch that limits conventional SOI grating antennas, the device achieved a longitudinal steering range of 34.9° over a 100‐nm wavelength‐tuning range while supporting two‐dimensional beam steering. This result underscores the role of platform‐level innovation in expanding the usable steering range of silicon OPAs.

At the system level, silicon OPAs have shown strong compatibility with advanced beamforming, imaging, communication, and LiDAR architectures. OPA‐based dynamic imaging systems have been integrated with ghost‐imaging techniques for high‐resolution tracking of fast‐moving targets [173, 174]. For multibeam operation, a 4×4 Butler‐matrix beamforming network enabled aliasing‐free wide‐FoV scanning with reduced system complexity [175], whereas fractal ”H‐tree” array designs facilitated broadband incoherent high‐resolution imaging and demonstrated potential for electron‐beam steering and multibeam imaging [176]. In optical wireless communication, high‐speed 2D scanning OPAs have demonstrated data transmission rates of up to 32 Gbps [177]. Chip‐scale FMCW LiDAR incorporating both transmit and receive OPAs has further demonstrated beam collimation and high‐precision detection, with grouped cascaded phase modulation lowering control complexity and underscoring the potential of fully integrated on‐chip LiDAR systems [26]. Silicon OPAs have also been integrated with optical microcomb sources to enable parallel FMCW LiDAR architectures, as illustrated in Figure 11c. In such a system, the silicon‐on‐insulator (SOI) platform serves as the core beam‐steering engine, where the OPA chip integrates a power splitter tree, a voltage‐controlled phase‐shifter array, and a sparse long‐grating antenna array, while operating in conjunction with the microcomb source and coherent detection modules. Beam steering in the horizontal direction is achieved through electrical phase tuning across the silicon OPA channels, whereas steering in the vertical direction is enabled by wavelength multiplexing of the comb lines. This architecture highlights an important advantage of the Si platform for OPAs: Beyond supporting dense active arrays and large‐field‐of‐view beam steering, silicon photonics can also provide a scalable interface to more complex system functionalities, including wavelength‐division multiplexing, coherent detection, and parallel ranging. Such compatibility makes silicon particularly attractive for high‐frame‐rate, highly integrated LiDAR transmitters.

A fundamental strength of the silicon platform is its exceptional scalability in large‐array integration. A landmark demonstration showcased a large‐scale 64×64 silicon nanophotonic phased array integrating 4096 optical nanoantennas within a 576×576 μm footprint [162]. In this architecture, optical power was uniformly distributed from a main silicon bus waveguide into row waveguides and then further divided into individual antenna units, whereas the phase of each pixel was controlled through integrated optical delay lines. This work highlighted a fundamental advantage of the Si platform: The maturity of CMOS‐compatible nanofabrication allows thousands of compact optical emitters and routing structures to be integrated on a single chip with sufficiently high phase uniformity and power balance, as shown in Figure 11d [162]. Subsequent developments have reported a 512‐channel OPA exhibiting submilliwatt power consumption per phase shifter, wide FoV (56°×15°), fast response (<30μs), and excellent beam performance (FWHM 0.04°, 12 dB sidelobe suppression), supporting 3D imaging and free‐space communication over distances from 10 to 185 m [27]. Taken together, these advances show that silicon remains one of the most mature and scalable OPA platforms, particularly when very large array sizes, dense active integration, and complex system‐level functionality are required. A comprehensive comparison of the key performance metrics of representative OPAs based on silicon‐photonic platforms is presented in Table 4.

**TABLE 4:** Performance comparison among some OPAs based on silicon‐photonic platforms.

Year (ref)	Principle	Power consumption	Speed	FoV	SLSR (dB)	FWHM	Channels
2025 [47]	TO	18.5 mW/π	NA	80°×53°	8	0.1°×0.037°	128
2024 [178]	TO	NA	NA	28.6°×6.1°	7.81	0.77°×4.23°	64
2024 [179]	TO	NA	NA	84°×13°	19.1	2.8°×1.7°	32
2024 [180]	TO	20 mW/π	NA	110°×28°	8.2	3.6°×0.4°	32
2024 [181]	TO	20 mW/π	NA	180°	24	21°	64
2024 [123]	TO	22 mW/π	50 kHz/20 μs	40°×8°	6.9	0.41°×0.076°	32
2024 [142]	TO	NA	NA	19.3°×39.7°	NA	45°×52°	8
2024 [81]	TO	3 mW/π	34 kHz	162°	12.8	0.13°	120
2023 [124]	TO	13.54 mW/2π	17 kHz	8°×8°	9.6	0.44°×0.44°	256
2023 [125]	TO	22.3 mW/2π (TE) 35.1 mW/2π (TM)	NA	60°×24.8°	12.1	3.7°×2° 3.7°×1.4°	16
2023 [73]	TO	2.7 mW/π	NA	25°×13.2°	15.1	0.31°×0.07°	120
2023 [130]	TO	300 μW/π	330 kHz	5.8°×5.8°	NA	0.6°×0.6°	8×8
2022 [29]	EO	NA	NA	100°×17°	10	0.01°×0.039°	8192
2022 [83]	TO	7 mW/π	NA	180°×13.5°	19	2.1°×0.08°	64
2021 [173]	TO	47 mW/2π	61 kHz	11°×22°	NA	2.5°×4.9°	4×4
2020 [177]	TO/EO	4.8 mW/π	NA	46°×10.2°	12	0.7°×0.9°	64
2020 [131]	TO	1.7 mW/π	NA	70°×6°	7.5	0.15°×0.08°	512
2020 [170]	EO	17 mW/π	20 MHz	8.9°×2.2°	8.8	0.92°×0.32°	8×8
2019 [27]	EO	2 μW	33.3 kHz	56°×15°	12	0.04°	512
2018 [129]	TO	54 mW/π	NA	45°	9	0.03°	1024
2017 [26]	TO	6.5 mW/π	NA	46°×36°	8	0.58°×0.18°	50

*Note:* NA indicates data not available in the referenced literature.

Abbreviations: EO, electro‐optic; FoV, field of view; FWHM, full width at half maximum; OPA, optical phased array; SLSR, sidelobe suppression ratio; TE, transverse electric; TM, transverse magnetic; TO, thermo‐optic.

### 5.2 ${Si}_{3}N_{4}$

${Si}_{3}N_{4}$ has emerged as a highly promising platform for integrated optical phased arrays (OPAs), owing to its low propagation loss, wide transparency window, high optical power‐handling capability, and CMOS‐compatible fabrication. Unlike silicon, whose transparency is limited to the 1.1–4‐μm range and which suffers from two‐photon absorption below 2.2 μm, ${\text{Si}}_{3}N_{4}$ offers a broad transparency window extending from the visible (approximately 400 nm) to the mid‐infrared (approximately 4 μm), while exhibiting negligible two‐photon absorption. These features make ${\text{Si}}_{3}N_{4}$ particularly attractive for high‐power and broadband photonic systems, including OPAs operating across the visible and near‐infrared bands. In addition, its relatively low thermo‐optic coefficient ($2.45\times 10^{-5}\;K^{-1}$ at 1.55 μm) helps suppress thermal drift, which is beneficial for beam‐steering systems sensitive to temperature fluctuations. ${\text{Si}}_{3}N_{4}$ waveguides can be fabricated using either low‐pressure chemical vapor deposition (LPCVD) or plasma‐enhanced chemical vapor deposition (PECVD), supporting multilayer integration and enabling high‐performance multifunctional PICs tailored for advanced OPA architectures.

A major advantage of the ${\text{Si}}_{3}N_{4}$ platform for OPAs lies in its suitability for passive or low‐power beam‐steering architectures based on low‐loss delay networks and wavelength‐dependent emission. To simplify control systems, reduce power consumption, and enhance operational stability, various passive two‐dimensional beam‐steering strategies have been developed on this platform. For example, wavelength‐tunable OPAs based on delay lines with engineered spacing demonstrated high‐efficiency 2D scanning of 0.44°/nm × 0.07°/nm with sidelobe suppression ratios (SLSR) of up to 12 dB, as shown in Figure 12a [182]. In this architecture, a passive OPA based on ${\text{Si}}_{3}N_{4}$ delay lines was implemented by integrating an edge coupler, a 1×2 MMI splitter tree, a set of U‐shaped delay lines, and a sidewall grating‐antenna array on the same chip. The fixed optical path difference introduced between adjacent channels converts wavelength tuning directly into a controlled phase gradient across the array, thereby enabling two‐dimensional beam scanning without the need for active phase shifters [182]. This design highlights a key advantage of the ${\text{Si}}_{3}N_{4}$ platform for OPAs: Because ${\text{Si}}_{3}N_{4}$ waveguides exhibit much lower effective‐index sensitivity to fabrication variations than silicon waveguides, the accumulated phase error in long delay‐line structures can be significantly suppressed. Similarly, shallow‐etched ${\text{Si}}_{3}N_{4}$ grating antennas have enabled stable and directional beam emission through cavity‐thickness optimization, achieving a total beam scan of 7.4° with divergence angles as low as 0.2°×0.6° [186]. A star‐coupler‐based 2D OPA architecture has also been demonstrated, offering high integration density and agile beam tuning with a compact beam spot of 0.24°×0.167° across a wide field of view of 15°×11.2°, thus supporting variable‐resolution scanning and random‐access beam steering compatible with FMCW LiDAR systems [187].

**FIGURE 12:**
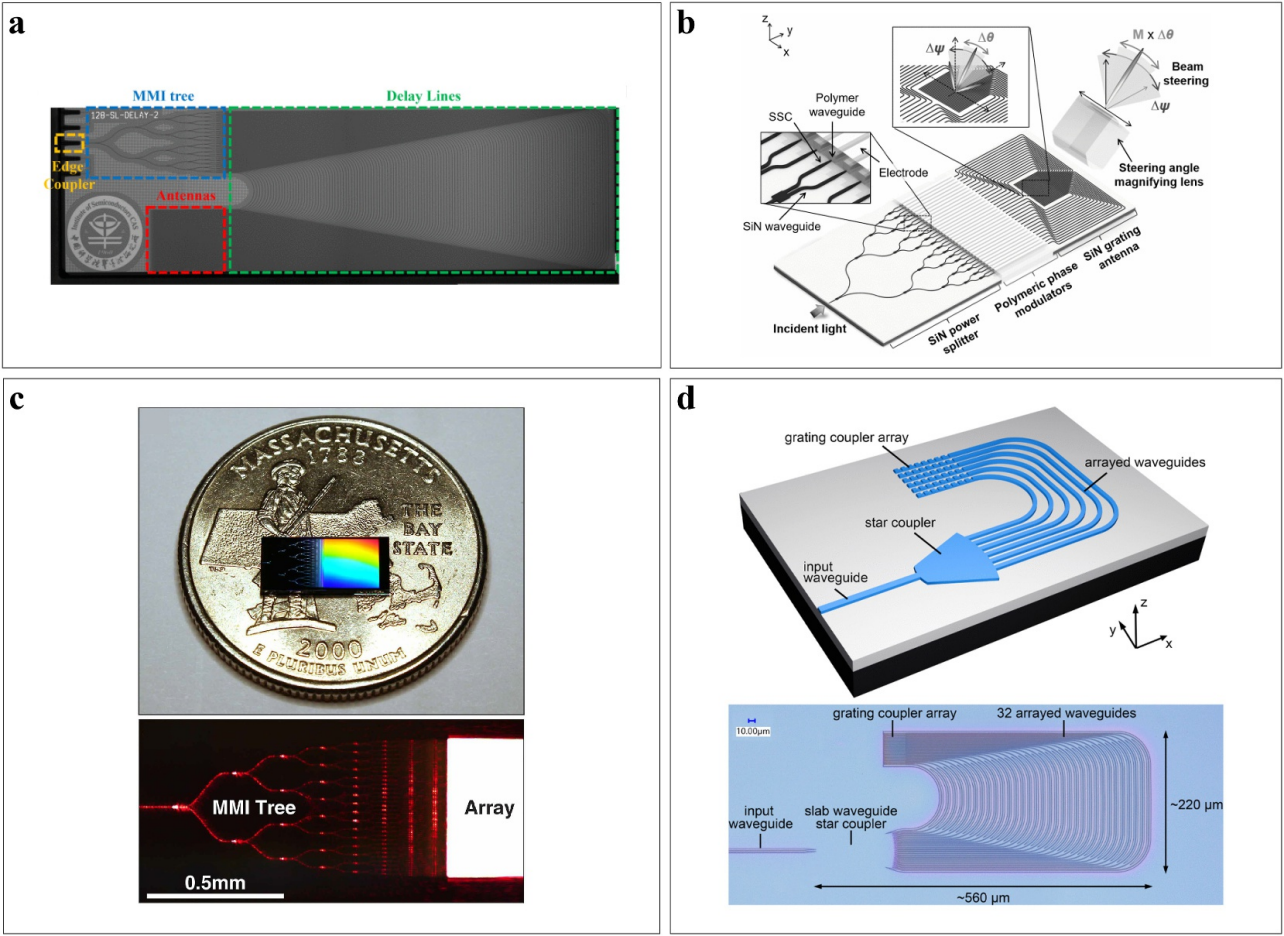
Optical phased array based on a silicon nitride photonic platform. (a) Passive optical phased array based on ${\text{Si}}_{3}N_{4}$ delay lines, which consists of an edge coupler, an MMI splitter tree, a delay‐line region, and a grating‐antenna array. By introducing a fixed path‐length increment between adjacent ${\text{Si}}_{3}N_{4}$ waveguides, the device enables wavelength‐controlled phase progression across the aperture and thus passive two‐dimensional beam scanning [182]. (b) Hybrid integrated ${\text{Si}}_{3}N_{4}$–polymer optical phased array for efficient beam steering, which consists of a ${\text{Si}}_{3}N_{4}$ power splitter, an array of polymeric phase modulators, and a ${\text{Si}}_{3}N_{4}$ grating antenna. The ${\text{Si}}_{3}N_{4}$ and polymer waveguides are butt‐coupled through a spot‐size converter, whereas an external steering‐angle magnifying lens is used to enlarge the wavelength‐tuned steering range in the longitudinal direction [183]. (c) Photograph of the 4 mm × 4 mm IR silicon nitride nanophotonic phased array after fabrication [184]. (d) ${\text{Si}}_{3}N_{4}$ optical phased array for compact spectral analysis. It combines the schematic of the integrated OPA spectrometer and the microscope image of the fabricated device. Light from the input waveguide is distributed by a star coupler into an array of waveguides with increasing lengths and then emitted out of plane by a grating‐coupler array so that different wavelengths are mapped to different far‐field directions. The fabricated ${\text{Si}}_{3}N_{4}$ OPA consists of 32 arrayed waveguides in a compact footprint [185].

Despite these advantages, the intrinsically low thermo‐optic coefficient of ${\text{Si}}_{3}N_{4}$ limits its efficiency for active phase tuning. To compensate for this limitation, heterogeneous integration with high‐thermo‐optic‐coefficient materials has been explored. In particular, polymer‐assisted hybrid integration has enabled efficient thermo‐optic modulation while preserving the low‐loss routing characteristics of ${\text{Si}}_{3}N_{4}$. This approach achieved a $P_{\pi }$ of about 2.5 mW and enabled beam steering over 12°×30°, together with time‐of‐flight ranging over distances beyond 10 m [183, 188]. As shown in Figure 12b, a ${\text{Si}}_{3}N_{4}$ power splitter and grating antenna are combined with polymeric phase modulators to form a two‐dimensional beam‐steering architecture for LiDAR. In this scheme, the ${\text{Si}}_{3}N_{4}$ section provides low‐loss optical routing and wavelength‐dependent grating emission, whereas the polymer section supplies efficient thermo‐optic phase modulation with much lower electrical power consumption than a purely ${\text{Si}}_{3}N_{4}$ implementation [183]. An adiabatic spot‐size converter is further introduced to reduce the coupling loss between the ${\text{Si}}_{3}N_{4}$ and polymer waveguides, and a steering‐angle magnifying lens is incorporated to enhance the wavelength‐tuned steering efficiency in the longitudinal direction [183]. This design clearly illustrates a key development direction for ${\text{Si}}_{3}N_{4}$‐based OPAs: Rather than relying solely on native ${\text{Si}}_{3}N_{4}$ functionality, hybrid material integration can be exploited to combine the low‐loss and high‐power‐handling advantages of ${\text{Si}}_{3}N_{4}$ with more efficient active tuning mechanisms, thereby extending the practical applicability of the platform in long‐range beam‐steering and LiDAR systems. In parallel, the low‐loss and high‐power‐handling capability of the ${\text{Si}}_{3}N_{4}$ platform has also supported multibeam beamforming architectures. A 32‐channel four‐beam two‐dimensional OPA based on ${\text{Si}}_{3}N_{4}$ achieved a field of view of 25.6°×8.1° with a narrow beam divergence of 0.655°×0.085°, underscoring the suitability of this platform for broadband, high‐power, and multiaccess free‐space optical links [189].

Another defining advantage of ${\text{Si}}_{3}N_{4}$ is its broad wavelength coverage, which enables OPA operation from the visible to the near‐infrared. Demonstrations include grating‐based arrays spanning 650–1064 nm with a 53.7° scanning range and an ultranarrow beam divergence of 0.03°, offering high beam resolution and continuous projection capability [71]. Recent studies have further expanded visible‐wavelength ${\text{Si}}_{3}N_{4}$ OPAs toward broader functionality and improved system performance. Wang et al. [190] demonstrated an 8‐channel ${\text{Si}}_{3}N_{4}$ OPA operating from 488 to 610 nm and achieved beam steering through phase, polarization, and wavelength tuning, highlighting the potential of this platform for broadband visible‐light beam steering. Extending visible‐light operation further, Zhang et al. [191] reported a 64‐element four‐directional circular ${\text{Si}}_{3}N_{4}$ OPA at 632.5 nm, representing the first experimental demonstration of continuous two‐dimensional beam steering with pure phase control in the visible range. In parallel, Yu et al. [192] showed that practical visible‐light OPA deployment also depends strongly on high‐speed driving and calibration electronics; using a 532‐nm SiN OPA integrated with an FPGA–DAC phase‐control system, they achieved beam calibration within 30–50 ms and voltage configuration in 7.4 μs, enabling rapid beam steering over ±24°.

${Si}_{3}N_{4}$ is also particularly attractive for large‐aperture OPAs because its low‐loss distribution networks can sustain high optical power with reduced phase‐error accumulation. As shown in Figure 12c, a large‐scale ${\text{Si}}_{3}N_{4}$ nanophotonic phased array with a 4mm×4mm aperture produced a near‐diffraction‐limited far‐field beam at 1550 nm [184]. The measured main‐lobe spot size reached 0.021°×0.021°, very close to the theoretical diffraction limit, indicating that little phase error accumulated in the ${\text{Si}}_{3}N_{4}$ distribution network and that the antenna elements remained highly uniform across the large aperture [184]. Such performance is difficult to achieve on conventional silicon distribution networks at high optical power, where nonlinear effects and fabrication‐induced phase errors more readily degrade beam quality.

Beyond beam steering itself, ${\text{Si}}_{3}N_{4}$‐based OPAs have also been extended toward multifunctional photonic systems across sensing, spectroscopy, biophotonics, and telecommunications. In conjunction with FMCW LiDAR, these OPAs have enabled adaptive 3D imaging, enhancing point‐cloud density by a factor of four in regions of interest without increasing the total number of scan points [193]. The ${\text{Si}}_{3}N_{4}$ platform has also enabled optical phased‐array architectures to be extended toward integrated spectral analysis [185]. As shown in Figure 12d, the proposed ${\text{Si}}_{3}N_{4}$ OPA spectrometer consists of an input waveguide, a star coupler, an array of waveguides with progressively increasing lengths, and a grating‐coupler array for off‐chip emission. In this configuration, the phased‐array waveguides introduce a wavelength‐dependent phase gradient, whereas the grating couplers provide an additional wavelength‐dependent diffraction response, such that each input wavelength is mapped to a unique far‐field direction [185]. By combining this on‐chip OPA with a free‐space Fourier imaging system, the device converts spectral information into spatially resolved far‐field spots that can be directly recorded by an image sensor. In optogenetics, implantable ${\text{Si}}_{3}N_{4}$‐based neural probes have demonstrated spatially addressable beam delivery within brain tissue, opening new possibilities for neural imaging and stimulation [194]. At telecommunication wavelengths, the integration capability of the ${\text{Si}}_{3}N_{4}$ platform has also been demonstrated in fully integrated phased‐array systems. Arsanjani et al. [195] reported a fully integrated 1×32 SiN OPA operating from 1500 to 1630 nm, achieving two‐dimensional beam steering with a field of view of 24.28°×9.46°. This result further confirms the versatility of ${\text{Si}}_{3}N_{4}$ for compact and scalable beam‐steering systems across different wavelength bands.

To further improve beamforming quality, machine learning‐assisted and optimization‐based design methods have also been introduced into ${\text{Si}}_{3}N_{4}$ OPA development. For example, particle swarm optimization (PSO) has been applied to engineer aperiodic emitter distributions, achieving a scanning range of 20.75° with excellent sidelobe suppression [196]. This approach also improves robustness against modal shifts and fabrication errors, providing a promising route toward highly dense and scalable OPA systems. A comprehensive comparison of the key performance metrics of representative OPAs based on ${\text{Si}}_{3}N_{4}$ platforms is presented in Table 5.

**TABLE 5:** Performance comparison among some OPAs based on silicon nitride platforms.

Year (ref)	Principle	Power consumption	Speed	FoV	SLSR (dB)	FWHM	Channels
2025 [135]	NA	NA	NA	25°×25°	7	0.55°×0.07°	65
2024 [197]	TO	130 mW/2π	NA	24.28°×9.4°	8.62	0.66°×0.082°	32
2023 [182]	NA	NA	NA	22.2°×9.3°	12	0.16°×0.13°	128
2023 [188]	TO	25.7 mW/π	14.5 kHz	30°	7.4	0.8°×6.8°	32
2022 [187]	NA	60 mW/π	NA	15°×11.2°	NA	0.24°×0.16°	23
2022 [183]	TO	25 mW/π	NA	32°×12°	NA	NA	32
2022 [71]	NA	NA	NA	53.7°	NA	0.03°×0.05°	32×32
2020 [186]	NA	NA	NA	7.4°	NA	0.2°×0.6°	32

*Note:* NA indicates data not available in the referenced literature.

Abbreviations: FoV, field of view; FWHM, full width at half maximum; OPA, optical phased array; SLSR, sidelobe suppression ratio; TO, thermo‐optic.

### 5.3 Si–${\text{Si}}_{3}N_{4}$

The Si–${\text{Si}}_{3}N_{4}$ heterogeneous integration platform has emerged as a highly promising solution for optical phased arrays (OPAs), owing to its complementary combination of efficient active control in silicon and low‐loss, high‐power optical routing in ${\text{Si}}_{3}N_{4}$. Although silicon waveguides provide compact and efficient phase tuning, as well as mature active‐device integration, ${\text{Si}}_{3}N_{4}$ waveguides offer ultralow propagation loss, high optical power‐handling capability, and reduced nonlinear penalties. This functional complementarity makes the hybrid platform particularly attractive for high‐performance OPAs that require wide field of view, high optical power, narrow beam divergence, and dense photonic integration.

Compared with single‐material OPA platforms, Si–${\text{Si}}_{3}N_{4}$ integration provides a more versatile route toward simultaneously achieving large aperture, high power tolerance, and compact active control. As shown in Figure 13a, a SiN‐on‐SOI Vernier OPA transceiver was developed by monolithically integrating a 128‐channel Tx‐OPA and a 128‐channel Rx‐OPA on the same chip [146]. In this architecture, cascaded MMIs distribute light into the array, thermo‐optic phase shifters provide independent phase control, and closely spaced Tx/Rx‐antenna arrays form a quasi‐coaxial transceiver geometry. By combining ${\text{Si}}_{3}N_{4}$ grating antennas with silicon‐based active‐control elements, the platform exploits the high‐power‐handling and low‐loss characteristics of ${\text{Si}}_{3}N_{4}$ while retaining the compact modulation and routing advantages of silicon [146]. The use of Vernier antenna arrays with different pitches further suppresses grating lobes over an ultrawide steering range, whereas the chained grating design broadens the single‐antenna far‐field envelope and helps maintain a nearly uniform beam‐power response across the field of view [146]. This work illustrates a key advantage of the Si–${\text{Si}}_{3}N_{4}$ platform: Passive radiation and high‐power optical handling can be delegated to the ${\text{Si}}_{3}N_{4}$ layer, whereas efficient phase tuning and active photonic circuitry remain in the silicon layer.

**FIGURE 13:**
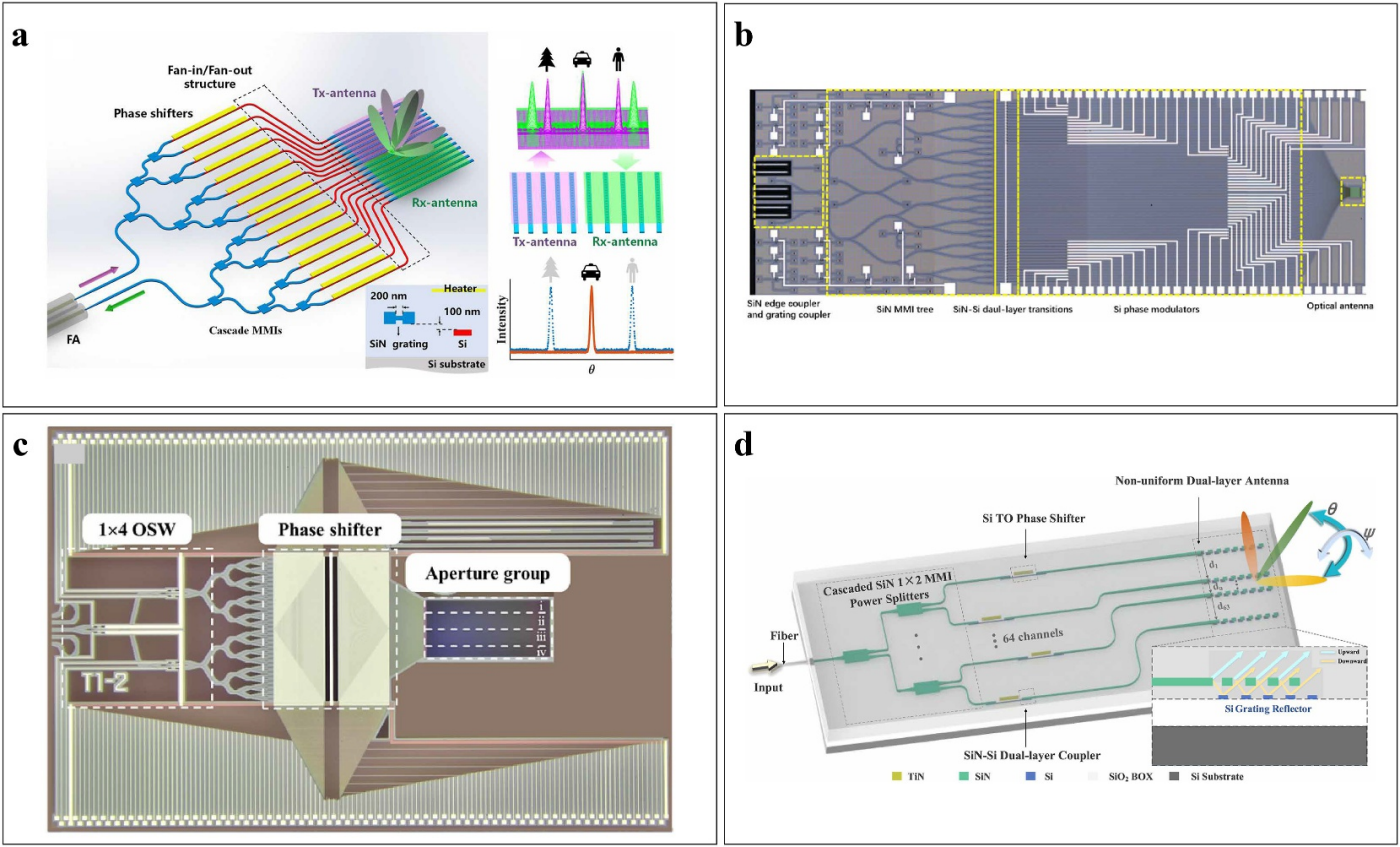
Optical phased array based on a silicon/silicon nitride photonic platform. (a) SiN‐on‐SOI Vernier optical phased‐array transceiver for ultrawide‐field‐of‐view LiDAR. It shows the monolithically integrated OPA architecture, including cascaded MMIs, phase shifters, fan‐in/fan‐out structures, and closely arranged Tx‐ and Rx‐antenna arrays that form a quasi‐coaxial transceiver. The inset illustrates the SiN‐on‐SOI waveguide cross‐section, where ${\text{Si}}_{3}N_{4}$ gratings are combined with silicon‐based photonic layers. This architecture exemplifies how the Si–${\text{Si}}_{3}N_{4}$ hybrid platform can merge high‐power low‐loss antenna operation with compact active beam control, enabling ultrawide‐FoV and LiDAR‐oriented OPA systems [146]. (b) Architecture of a ${\text{Si}}_{3}N_{4}$–Si dual‐layer optical phased‐array chip. It shows the fabricated OPA layout, including a ${\text{Si}}_{3}N_{4}$ input coupler, a ${\text{Si}}_{3}N_{4}$ MMI splitter tree, ${\text{Si}}_{3}N_{4}$–Si dual‐layer transitions, silicon phase modulators, and a silicon optical antenna. By routing and splitting light in the low‐loss ${\text{Si}}_{3}N_{4}$ layer and performing phase modulation and beam emission in the silicon layer, this dual‐layer architecture combines the advantages of both materials for improved OPA performance [198]. (c) Reconfigurable‐aperture optical phased array on a Si–${\text{Si}}_{3}N_{4}$ hybrid platform. It shows the optical microscope image of the fabricated chip, including a mode‐size converter, a 1×4 optical switch, cascaded splitters, phase shifters, and an aperture group. By selectively routing light into different subapertures, the device can switch its effective aperture size from 64 to 256 channels [199]. (d) Schematic of a ${\text{Si}}_{3}N_{4}$–Si dual‐layer optical phased array. It shows the overall architecture of the proposed OPA, including a ${\text{Si}}_{3}N_{4}$ fiber‐chip coupler, cascaded ${\text{Si}}_{3}N_{4}\;1\times 2$ MMI power splitters, ${\text{Si}}_{3}N_{4}$–Si dual‐layer couplers, silicon thermo‐optic phase shifters, and a SiN–Si nonuniform dual‐layer antenna [75].

One of the most important motivations for Si–${\text{Si}}_{3}N_{4}$ integration is the need to handle high optical power without incurring the nonlinear penalties of fully silicon distribution networks. Addressing the stringent power demands of LiDAR, several high‐power OPA implementations have been reported [200]. A representative 64‐channel SiN–Si hybrid OPA operating at 1550 nm achieved a main‐lobe output of 720 mW across a ±55° scanning range with sidelobe suppression of −10.33 dB. In this design, ${\text{Si}}_{3}N_{4}$ waveguides serve as high‐power conduits to avoid nonlinear penalties in silicon, particularly two‐photon absorption (TPA) and the associated free‐carrier absorption (FCA), whereas silicon waveguides provide efficient phase control. The system power ceiling is, therefore, no longer dictated by a fully silicon routing network, suggesting an effective scaling route through a larger channel count.

Multilayer vertically stacked ${\text{Si}}_{3}N_{4}$–Si OPA architectures have further strengthened this design paradigm. As shown in Figure 13b, the proposed dual‐layer OPA chip consists of a ${\text{Si}}_{3}N_{4}$ input coupler, a cascaded ${\text{Si}}_{3}N_{4}$ MMI splitter tree, ${\text{Si}}_{3}N_{4}$–Si interlayer transitions, silicon phase modulators, and a silicon optical antenna [198]. In this architecture, high‐power input light is first routed and distributed in the low‐loss ${\text{Si}}_{3}N_{4}$ layer, thereby reducing propagation loss and mitigating nonlinear penalties that would otherwise accumulate in a fully silicon distribution network. After power splitting, the optical signals are transferred into the silicon layer through dual‐layer transitions, where efficient thermo‐optic phase tuning and beam emission are performed. Such a division of functionality clearly exploits the complementary strengths of the two materials: ${\text{Si}}_{3}N_{4}$ provides low‐loss and high‐power optical routing, whereas silicon offers compact and efficient phase modulation together with mature active‐device integration [198].

Balancing a wide field of view and high angular resolution remains another central challenge for hybrid OPA platforms. To this end, two 128‐channel architectures, namely, fishbone and chained antenna arrays, were demonstrated on multilayer SiN‐on‐SOI platforms [70]. By employing nonuniform antenna spacing, these systems achieved a 4‐mm aperture with only 128 waveguides. The fishbone OPA provided a 100°×19.4° FoV with a record‐low beam divergence of 0.021°×0.029°, whereas the chained variant achieved a record‐wide 140° horizontal FoV. Both architectures employed reverse‐biased p‐n junction phase shifters and exhibited ultralow static power consumption of 1.8 μW. In FMCW LiDAR demonstrations, a 100‐m ranging capability with a 3.38‐cm error and a 5.09‐cm resolution was reported, highlighting the potential of the Si–${\text{Si}}_{3}N_{4}$ platform for high‐resolution long‐range imaging. In terms of phase control, cointegration of silicon phase modulators with ${\text{Si}}_{3}N_{4}$ input networks enabled π‐phase‐tuning efficiencies better than 17.5 mW while substantially improving power tolerance [201]. The use of nonuniform element spacing further compressed beam divergence and improved spatial resolution for multiobject separation [202].

In parallel with these high‐performance beamforming architectures, the Si–${\text{Si}}_{3}N_{4}$ platform has also enabled new approaches to reducing control complexity and improving aperture scalability. To improve scalability in large‐scale OPAs, a novel SOPA (serpentine OPA) architecture was proposed [30]. It features cascaded serpentine paths built from low‐loss waveguides and achieves passive two‐dimensional beam steering through wavelength tuning, thereby significantly simplifying control logic and reducing power consumption. With a nearly 100% array fill factor, this architecture supports subarray tiling to scale apertures to centimeter size. A single chip demonstrated 16,500 addressable points and multi‐subarray far‐field interference, laying the groundwork for on‐chip synthetic‐aperture imaging.

The high integration density of the Si–${\text{Si}}_{3}N_{4}$ platform has also enabled system‐level cointegration of key OPA subsystems, including lasers, modulators, switches, splitters, and antennas. A multilayer SiN–Si‐based LiDAR transmitter chip combined an ultranarrow‐linewidth laser with a 2D nonuniform OPA, achieving an exceptionally narrow beam waist of 0.051°×0.016° over a 140°×16° FoV [76]. Adaptive and reconfigurable OPA designs have also been proposed to dynamically scale the array size according to target distance or frame rate, thereby optimizing scanning time and energy consumption for intelligent beam management [199], as shown in Figure 13c. The reconfigurable‐aperture OPA was realized on a silicon/silicon nitride multilayer platform integrating a mode‐size converter, a 1×4 optical switch, cascaded Y splitters, thermo‐optic phase shifters, and an aperture group composed of multiple subapertures [199]. In this architecture, ${\text{Si}}_{3}N_{4}$ waveguides are used for low‐loss optical routing, whereas silicon waveguides and heaters provide efficient active switching and phase tuning. By controlling the optical‐switch network, the effective transmitting aperture can be reconfigured from 64 to 256 channels, thereby enabling dynamic adjustment of the beam divergence angle and far‐field formation distance [199]. Such a capability is particularly important for LiDAR‐oriented OPAs because it allows the same chip to support both coarse wide‐area scanning and fine high‐resolution target interrogation.

As array size and system complexity continue to increase, on‐chip calibration and system optimization become increasingly important. Fast, low‐complexity calibration schemes based on compact phase indicators and noniterative physical models have enabled real‐time tuning without far‐field feedback, reducing beam divergence to below 0.56° [203, 204]. Validated on a 64‐channel OPA, this method supports wavelength‐agnostic two‐dimensional beam steering. Through further refinements such as nonuniform antenna layouts and Si‐assisted grating reflectors, SiN–Si stacked OPAs have reached 120°×13.9° scanning while maintaining total system losses below 12.66 dB [75], as shown in Figure 13d. The proposed SiN–Si dual‐layer OPA consists of a ${\text{Si}}_{3}N_{4}$ fiber‐chip coupler, cascaded ${\text{Si}}_{3}N_{4}$ power splitters, ${\text{Si}}_{3}N_{4}$–Si dual‐layer couplers, silicon thermo‐optic phase shifters, and a SiN–Si nonuniform dual‐layer antenna [75]. In this architecture, optical power is first coupled and distributed in the ${\text{Si}}_{3}N_{4}$ layer, thereby reducing propagation loss and mitigating nonlinear penalties associated with routing high optical power in silicon. The optical signals are then transferred into the silicon layer for efficient phase modulation, whereas the dual‐layer nonuniform antenna is used to simultaneously suppress grating lobes and reduce emission loss [75].

Beyond LiDAR, Si–${\text{Si}}_{3}N_{4}$‐based OPAs also show strong potential for free‐space optical communication and 3D sensing. A 128‐channel beam‐steering chip enabled error‐free transmission of 32 Gb/s NRZ signals over 54 m with sub‐27‐μs beam switching, providing a robust solution for V2X and smart‐device communication [202]. In three‐dimensional sensing, wavelength‐tuned phase‐scanning architectures expanded the FoV to approximately 32°×36°, thereby improving spatial resolution and scene adaptability [205]. For ranging applications, SiN–Si hybrid OPAs support both ToF and FMCW modalities, achieving detection ranges of 20 and 109 m, respectively [206]. Taken together, these results confirm that the Si–${\text{Si}}_{3}N_{4}$ platform is particularly well suited for OPA systems that simultaneously require high optical power, low‐loss routing, efficient active control, and tight system‐level integration. A comprehensive comparison of the key performance metrics of representative OPAs based on Si–${\text{Si}}_{3}N_{4}$ heterogeneous integration platforms is presented in Table 6.

**TABLE 6:** Performance comparison among some OPAs based on Si‐${\text{Si}}_{3}N_{4}$ heterogeneous integration platforms.

Year (ref)	Principle	Power consumption	Speed	FoV	SLSR (dB)	FWHM	Channels
2025 [48]	TO	NA	NA	10.5°×10.5°	34.5	1.3°×1.3°	8×8
2024 [199]	TO	15.3 mW/2π	NA	22.8°	10	0.08°	256
2023 [193]	TO	NA	NA	27.2°×8.52°	NA	0.4°×0.85°	128
2023 [146]	TO	6 mW/π	NA	160°×3°	NA	0.16°×0.04°	256
2023 [202]	EO	NA	37 kHz	100°×2.1°	10.63	0.021°×0.029°	128
2023 [76]	TO	NA	NA	140°×16.7°	7.8	0.051°–0.016°	256
2023 [75]	TO	18.79 mW/π	NA	120°×13.9°	11.4	0.052°–2.72°	64
2022 [206]	TO	NA	NA	96°×14.4°	9.7	1.41°×1.49°	64
2022 [204]	TO	30 mW/π	NA	38.4°	11	0.97°×0.58°	32
2021 [201]	TO	17.5 mW/π	NA	35.5°×22.7°	NA	0.69°×0.075°	64
2021 [70]	EO	1.8 μW	NA	140°×19.23°	10	0.021°×0.1°	128

*Note:* NA indicates data not available in the referenced literature.

Abbreviations: EO, electro‐optic; FoV, field of view; FWHM, full width at half maximum; OPA, optical phased array; SLSR, sidelobe suppression ratio; TO, thermo‐optic.

### 5.4 Lithium Niobate on Insulator

Lithium niobate on insulator (LNOI) has rapidly emerged as a key material platform for high‐performance optical phased arrays (OPAs), owing to its unique material properties and compatibility with advanced nanofabrication. Compared with conventional silicon or silicon nitride platforms, LNOI offers a broader optical transparency window (350–5000 nm), a larger Pockels electro‐optic coefficient ($\gamma_{33}=30.9$ pm/V), and ultralow optical propagation loss (as low as 0.027 dB/cm) [207]. These characteristics enable efficient phase modulation from the visible to the mid‐infrared while supporting low‐loss optical routing and compact phase‐shifter layouts. Its high‐index‐contrast waveguide geometry allows tight optical confinement and narrow electrode–waveguide spacing, thereby enabling sub‐volt driving and compact device footprints. Recent advances in nanofabrication have further enabled etched LNOI waveguides to support electro‐optic modulators with bandwidths exceeding 100 GHz and increasing integration density [208, 209], providing a strong foundation for low‐power, wide‐field‐of‐view, and high‐speed OPA systems. In contrast to silicon‐based platforms, which suffer from two‐photon absorption and poor visible transparency, LNOI also offers superior power handling, broader spectral operability, and strong potential for scalable integrated beam steering.

The mainstream development of LNOI‐based OPAs has centered on channelwise electro‐optic phase control with high speed and low energy consumption. Demonstrations of LNOI OPAs have already shown impressive electro‐optic performance, with reported modulation energies as low as 1.11 nJ/π and response times down to 14.4 ns. Devices with 32 and 48 channels have achieved far‐field scanning ranges of 62.2°×8.8° and 40°×8.8°, respectively, validating the platform’s potential for large‐scale integration and beamforming performance [42, 45]. As shown in Figure 14a, the demonstrated integrated LN OPA consists of input waveguides, cascaded multimode interferometers for power splitting, electro‐optic phase modulators for channelwise phase control, and a closely spaced grating‐waveguide array for out‐of‐plane radiation [45]. In this architecture, light is first coupled into the LN waveguide and then equally distributed into multiple channels, after which the phase of each channel is tuned through the Pockels effect using metal electrodes placed beside the waveguides [45]. The phase‐modulated signals are finally routed into a dense grating‐waveguide array to generate a steerable far‐field beam, whereas wavelength tuning provides beam steering in the orthogonal direction. Compared with thermally tuned silicon OPAs, this scheme highlights a central advantage of the LNOI platform: Beam steering can be achieved with much lower power consumption and much higher modulation speed without introducing the additional free‐carrier loss associated with carrier‐based silicon phase shifters [45].

**FIGURE 14:**
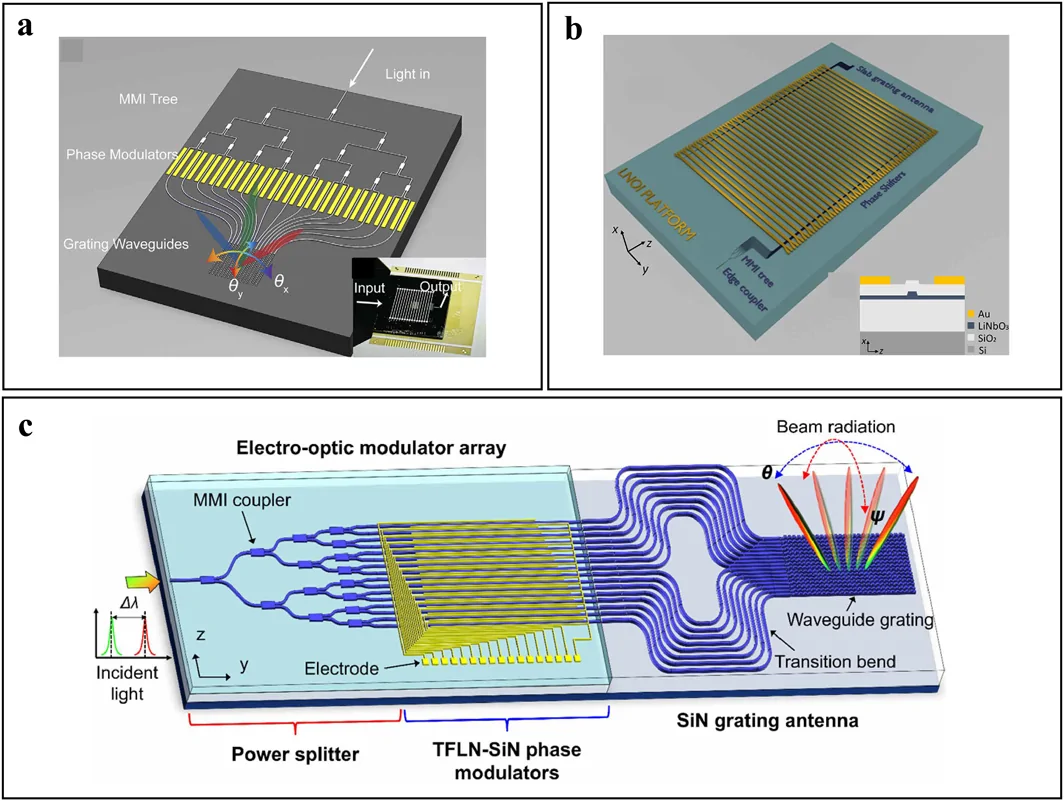
Optical phased array based on TFLN. (a) Integrated LN OPA and optical image of the OPA chip wire‐bonded to a PCB [45]; (b) visible‐light optical phased array on a thin‐film ${\text{LiNbO}}_{3}$ platform and cross‐sectional view of the chip [133]; (c) integrated electro‐optical phased array on a thin‐film ${\text{LiNbO}}_{3}$ (TFLN) and silicon nitride (SiN) hybrid platform [43].

Beyond basic phase‐shifter integration, recent work has further improved LNOI OPA beam quality and scanning performance through structural codesign. Beam quality has been further improved using X‐cut LNOI devices incorporating MMI splitters and etched grating structures, resulting in a 50° one‐dimensional scanning range with sidelobe suppression better than −10 dB [197]. A recent advance further showed that TFLN OPAs can simultaneously improve beam quality and steering performance through structural codesign. Li et al. [210] demonstrated a 16‐channel X‐cut TFLN OPA combining a superlattice ridge‐waveguide array, PSO‐optimized nonuniform spacing, and a trapezoidal grating emitter, achieving a narrow beam of 0.99°×0.63°, a sidelobe suppression ratio of 20 dB, and a two‐dimensional steering range of 47°×9.36°. This work highlights the promise of LNOI for compact OPAs with improved beam quality and wide‐angle steering. A traveling‐wave electrode design has also enabled LNOI OPAs with a 2.5‐GHz modulation bandwidth and a 50°×8.6° field of view while utilizing aperiodic waveguide arrays to suppress grating lobes [44]. Ma et al. [211] further demonstrated a 16‐channel TFLN OPA with electro‐optic phase shifters and optimized curved coupled trapezoidal grating antennas, achieving a field of view of 62°×11°, a far‐field FWHM of 3.8°×1.72°, and a minimum sidelobe suppression ratio of −13.6 dB. With a measured half‐wave voltage of 4.7 V, an energy efficiency of 8.73 pJ/π, and an average transition time of only 158 ps, this work further underscores the advantages of the LNOI platform for ultrafast and low‐power electro‐optic beam steering [211].

In parallel with these conventionally addressed EO OPA architectures, LNOI has also enabled a distinct route toward reduced control complexity through ferroelectric‐domain engineering. In terms of device architecture, OPA design is evolving toward more integrated and efficient phase‐generation schemes. Li et al. proposed a cascaded‐domain‐engineered OPA structure that leverages the opposite Pockels coefficients in alternating ferroelectric domains to achieve phase modulation using a single control signal, enabling uniform modulation across a 32‐element array with beam scanning at up to 3 MHz while significantly reducing control complexity [46]. Building on this, Li et al. demonstrated a multilayer cascaded‐domain structure for two‐dimensional beam steering, achieving ±20°×±16° scanning with only two control inputs and without relying on laser wavelength tuning, thereby addressing key bottlenecks in power consumption and control density in conventional 2D OPAs [212]. Li et al. further combined cascaded‐domain‐engineered LN waveguides with wavelength tuning in a single‐electrode‐controlled design, realizing a 42°×9.2° two‐dimensional scanning range and offering an elegant trade‐off between compactness and functionality [119]. These studies indicate that LNOI is not only suitable for high‐speed conventional EO phase shifters but also uniquely compatible with structurally encoded phase‐generation architectures that can substantially reduce the number of independent electrical control channels.

Beyond high‐speed beam steering at telecommunication wavelengths, another important extension of the LNOI platform is its expansion into the visible regime. A 32‐channel OPA on an X‐cut TFLN platform demonstrated a response time under 15 ns and a beam divergence of 0.41°×1.14°, while maintaining a 40° field of view, marking a significant step toward high‐speed and high‐resolution visible‐light beam control for emerging applications such as holography, biosensing, and display technologies [133]. As shown in Figure 14b, the demonstrated visible‐light OPA on a TFLN platform integrates an edge coupler, cascaded multimode interference splitters, electro‐optic phase shifters, and a nonuniform waveguide array combined with a slab grating antenna [133]. In this architecture, the input light is distributed into 32 channels and independently phase‐modulated through GS‐configured electro‐optic phase shifters, whereas the slab grating enables out‐of‐plane emission and the nonperiodic array arrangement helps suppress grating lobes [133]. Such a design highlights an important advantage of the LNOI platform for OPAs: Unlike thermo‐optically tuned Si or ${\text{Si}}_{3}N_{4}$ devices, TFLN can provide nanosecond‐scale beam control with low insertion loss and without sacrificing operation in the visible band.

To balance modulation efficiency and low‐loss radiation performance, hybrid TFLN–SiN OPA architectures have also been explored, as shown in Figure 14c. By integrating TFLN on SiN strip‐loaded waveguides, this approach avoids contamination and sidewall roughness associated with direct TFLN etching. Combined with cascaded MMI couplers and SiN grating antennas, the resulting 16‐channel device achieved a half‐wave voltage of 3.64 V, an energy per π phase shift of just 3.2 pJ, and beam steering of 22° (wavelength tuning) ×5° (phase tuning), with beamwidths of 1.2°×0.2° [43]. Such a design highlights an important development direction for the LNOI platform: Rather than relying exclusively on directly etched lithium niobate structures for all functions, the strong electro‐optic response of TFLN can be combined with the low‐loss and better‐confined SiN antenna platform to balance modulation efficiency, radiation performance, and fabrication practicality.

In parallel, efforts to improve the front‐end optical interface of LNOI‐based OPAs have leveraged integrated metasurfaces. A chip‐scale metasurface doublet lens with a quadratic phase profile was shown to expand the field of view by 1.54× with approximately 41% transmission efficiency while avoiding bulky free‐space alignment [213]. Meanwhile, a polarization‐multiplexed port‐selective OPA (POPA) based on aperiodic waveguide arrays and metasurfaces achieved 41.04°×7.06° scanning with sidelobe suppression above 10 dB and enabled dual‐beam output for enhanced frame rate and parallelism [214].

Taken together, these developments show that the LNOI platform is evolving along three closely related directions: high‐speed and low‐power conventionally addressed EO OPAs, reduced‐complexity phase‐generation architectures enabled by ferroelectric‐domain engineering, and broader platform extensions including visible‐light operation, hybrid TFLN–SiN integration, and metasurface‐enhanced front‐end optics. This progression is positioning LNOI as one of the most versatile and high‐performance OPA platforms for next‐generation solid‐state beam steering, with strong potential for LiDAR, free‐space optical communication, dynamic imaging, and human–machine interaction. A comprehensive comparison of the key performance metrics of representative OPAs based on LNOI is presented in Table 7.

**TABLE 7:** Performance comparison among some OPAs based on LNOI.

Year (ref)	Principle	Power consumption	Speed	FoV	SLSR (dB)	FWHM	Channels
2025 [133]	Pockels EO	NA	12 ns	40°	7.5	0.41°×1.14°	32
2025 [119]	Pockels EO	NA	NA	42°×9.2°	NA	31°–11°	1
2024 [45]	Pockels EO	13.5 pJ/π	0.23 ns	24°×8°	10	2°×0.6°	16
2024 [44]	Pockels EO	0.33 nJ/π	0.4 ns	50°×8.6°	5.1	0.73°×2.8°	16
2024 [42]	Pockels EO	1.11 nJ/π	14.4 ns	62.2°–8.8° 40°–8.8°	7.4 5.0	2.4°–1.2° 0.33°–1.8°	32 48
2024 [197]	Pockels EO	NA	NA	50°	10	NA	8
2024 [43]	Pockels EO	3.2 pJ/π	0.83 ns	22°×5°	NA	1.2°×0.2°	16
2024 [49]	Wavelength	NA	NA	15°	NA	1.6°	16
2023 [46]	Pockels EO	NA	333.3 ns	10°	NA	0.579°	1

*Note:* NA indicates data not available in the referenced literature.

Abbreviations: EO, electro‐optic; FoV, field of view; FWHM, full width at half maximum; LNOI, lithium niobate on insulator; OPA, optical phased array; SLSR, sidelobe suppression ratio.

### 5.5 Other Platforms

As performance demands for integrated optical phased‐array (OPA) systems continue to increase across applications such as LiDAR, free‐space optical communication, infrared imaging, and high‐speed beam control, the limitations of mainstream silicon‐based platforms have become increasingly apparent in areas such as optical power handling, intrinsic electro‐optic modulation efficiency, and mid‐infrared transparency. To address these constraints, increasing attention has turned to alternative material platforms that can provide monolithic gain integration, stronger electro‐optic response, and broader spectral coverage. Representative examples include group III–V semiconductors such as InP, InGaAs/InP, and GaAs, as well as germanium–silicon (GeSi) and hybrid material systems.

Among these alternatives, the InP platform has demonstrated particularly strong potential for highly integrated and high‐coherence OPAs. An 8‐channel OPA fabricated on a generic InP photonic integration platform exhibited stable operation over a 70‐nm wavelength span, using a star coupler for uniform power distribution and an mREV‐based calibration algorithm to improve phase accuracy, thereby establishing a scalable route toward wavelength‐discriminated two‐dimensional beam steering [215]. Further progress has been enabled by the monolithic integration of optical amplifiers, where the first full characterization of differential phase noise (DPN) between channels showed less than 10 mrad RMS phase error without phase‐locked loops, enabling high‐coherence beam formation in simplified LiDAR systems [216]. A 100‐element InP‐based OPA array achieved a narrow beamwidth of 0.11° and more than 80 resolvable points within a 7‐mm × 5 mm footprint, together with a sub‐16‐ns response time, highlighting the platform’s capability for high‐resolution and high‐speed beam control [134]. In addition, a 32‐channel OPA employing reverse‐biased p–i–n phase shifters achieved beam steering over 35° with a divergence angle of 0.46° and total power consumption below 7.5 mW, offering an attractive balance of modulation efficiency and precision [217].

For mid‐infrared OPA operation, the InGaAs/InP platform provides excellent spectral compatibility and thermal stability, particularly when combined with monolithically integrated quantum cascade lasers (QCLs). A 32‐channel OPA operating at 4.6 μm demonstrated ±11.5° lobe‐free steering while avoiding the thermal‐mismatch issues commonly associated with heterogeneous Si/Ge bonding [218]. Complementary efforts incorporating passive InGaAs/InP waveguides and Costas nonredundant array configurations extended two‐dimensional steering fields to 28°×28°, whereas a total‐internal‐reflection‐based emitter design further improved beam quality and compactness, supporting advanced mid‐infrared applications such as directed energy and secure communication [219]. Beyond beam steering, a 30‐channel InP active OPA was also used to construct a reconfigurable focusing lens capable of near‐diffraction‐limited beam focusing with a 2.7‐μm FWHM and dynamic confocal depth sensing, illustrating the broader potential of III–V OPAs for integrated imaging and microscopy‐related functions [220].

The GaAs platform exhibits competitive advantages in the near‐infrared regime, especially around the 1‐μm band. A 16‐channel OPA operating at 1064 nm achieved a beamwidth of 0.92°, a steering range of 15.3°, and 12 dB sidelobe suppression [221]. Fabricated using a relatively low‐complexity process, the device employed 3 mm reverse‐biased p–i–n modulators with static power consumption as low as 5 μW and bandwidths exceeding 770 MHz. Across the 980–1360‐nm range, it maintained favorable modulation efficiency (0.5–1.22 V⋅cm) together with very low residual amplitude modulation (below 0.5 dB), highlighting the suitability of GaAs for compact, thermally efficient, and high‐speed OPA systems in the near‐infrared [221].

For mid‐wavelength infrared (MWIR) beam steering, GeSi platforms provide an attractive combination of CMOS‐compatible processing and broad mid‐infrared transparency. A recent GeSi‐based two‐dimensional OPA demonstrated a steering field of 12.7°×12° and a beam divergence of 3.08°×0.18° within the 2–14‐μm window [95]. The platform also supports integration with QCLs, making it a promising route for mid‐infrared LiDAR, free‐space communication, and spectroscopy systems that require compatibility with semiconductor manufacturing and extended spectral coverage [95].

Beyond these single‐platform implementations, hybrid material systems are also emerging as an alternative route for on‐chip OPAs when phase modulation and beam emission cannot be optimally realized within one material platform. Miyamoto et al. [222] demonstrated a dual‐layer OPA based on an electro‐optic polymer–SiN hybrid architecture, in which EO polymer waveguides provided high‐speed, low‐power phase modulation and SiN waveguides enabled dense output emission. By introducing a tapered SiN interlayer transition, the 16‐channel device achieved a beam divergence of 1.917° and a maximum steering angle of 36.64°, highlighting the promise of hybrid platforms for combining fast electro‐optic tuning with compact output apertures and relatively large‐angle beam steering.

Taken together, these alternative material platforms complement mainstream Si, ${\text{Si}}_{3}N_{4}$, and LNOI approaches by extending the accessible design space toward monolithic gain integration, high‐speed electro‐optic control, stronger optical power handling, and broader infrared operation. In particular, III–V materials are especially attractive when active functionality and high coherence are required, GeSi offers a promising CMOS‐compatible route to the mid‐infrared, and hybrid material systems provide additional flexibility when phase tuning and beam emission are best distributed across different materials.

## 6 Main Challenges

### 6.1 Reduction of Electronics

Recent advances in chip‐scale optical phased arrays (OPAs) with large numbers of antenna elements have pushed system requirements toward higher modulation speed, finer beam‐steering resolution, and wider field‐of‐view coverage, all of which place increasing pressure on the underlying control architecture [162]. Although scaling the number of optical channels is not intrinsically prohibitive from a fabrication perspective [127], conventional OPA designs typically rely on independent phase control of each optical channel. As a result, the electronic complexity grows rapidly with array size, owing both to the need for hundreds or thousands of electrical interconnects and to the burden of increasingly complex driver circuitry for phase control [224]. Reducing the electronic overhead of large‐scale OPAs has, therefore, become a key architectural objective.

One major route toward this goal is cascaded or hierarchical phase generation, in which the required interelement phase progression is produced collectively rather than by fully independent channel‐by‐channel control. An early example was introduced by Tomas and Fainman, who demonstrated a fully addressable two‐stage PLZT‐based OPA capable of resolving 32 output positions with only 12 control voltages [225]. This seminal work laid the foundation for a series of cascading strategies aimed at substantially reducing control complexity in large‐scale OPAs. As shown in Figure 15a, a cascaded phase‐shifting architecture was later proposed, in which all phase shifters are driven by the same input voltage, whereas a nearly constant phase difference is generated between adjacent antennas through the cascaded optical layout [223]. In contrast to conventional OPA schemes that require a lookup table and independent settling of multiple phase shifters for each steering angle, this architecture establishes an approximately direct mapping between a single electrical control signal and the beam angle [223]. As a result, the steering speed is no longer limited by repeated multichannel phase stabilization, and the total control complexity is significantly reduced. More importantly, unlike architectures in which the required steering power increases rapidly with array size, the cascaded phase‐shifting scheme offers a more scalable route to continuous beam steering with lower electronic overhead. This work, therefore, represents an early and influential example of reducing OPA electronics through structural phase‐generation design rather than purely through driver‐circuit optimization.

**FIGURE 15:**
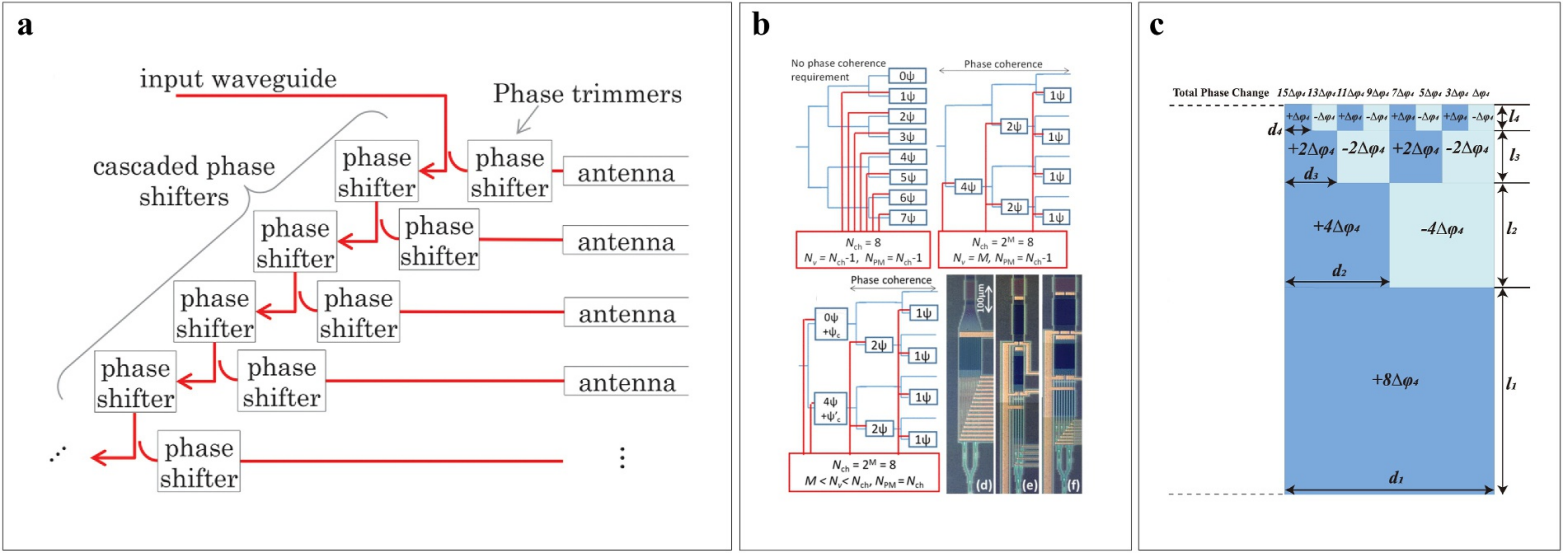
Representative schemes for reducing electronic control complexity in optical phased arrays. (a) Cascaded thermo‐optic phase shifters using a directional‐coupler‐based light distribution network with reduced control units [223]. (b) Architectures for reducing the number of control voltages in a binary‐tree optical phased array: a conventional OPA with individual phase control for each channel, a fully cascaded phase‐control architecture, and a hybrid cascaded architecture that combines individual and shared control. Optical microscope images of fabricated 16‐channel and 32‐channel devices are also shown [127]. (c) Phase‐generation principle of a single‐electrode cascaded‐domain‐engineering optical phased array. It shows the four‐layer cascaded ferroelectric‐domain structure, including the dimensions of each layer, the internal phase changes induced in different domain regions under a single applied voltage, and the cumulative phase profile after light propagates through the entire structure. By exploiting the opposite electro‐optic phase shifts in positive and negative lithium niobate domains, the device produces an equal phase difference between adjacent elements in the final layer, enabling beam steering with only one control electrode [46, 119].

An integrated OPA with a cascaded phase‐shifting architecture was subsequently reported by Shiyang et al., achieving continuous beam steering with only $\log_{2}N$—or even a single—control input [226]. This approach minimizes the number of control units by equalizing the optical path lengths across channels. Nevertheless, unavoidable fabrication imperfections introduce geometrical variations in waveguide dimensions, leading to cumulative phase errors, particularly in the longer waveguides required for high channel counts. Although combining local tuning with cascaded phase control can compensate for these errors, such hybrid approaches often reintroduce some of the system‐level complexity that the cascade design originally sought to avoid [127]. As shown in Figure 15b, a useful design route is to embed phase control directly into the binary splitter tree and distribute it hierarchically. In a conventional architecture, each optical channel is assigned an independent phase modulator, so the number of control voltages scales almost linearly with the channel count [127]. By contrast, a fully cascaded architecture allows phase shifters belonging to the same splitter stage to share a common control voltage, thereby significantly reducing the required number of electrical inputs. However, this simplification comes at the cost of a stronger dependence on optical path matching and fabrication‐induced phase errors. To balance these competing requirements, a hybrid‐cascaded architecture was proposed, in which the early stages retain individual control for phase‐error correction, whereas the later stages adopt shared cascaded control to reduce the total number of control voltages [127].

Beyond cascaded splitter‐tree architectures, a second route to reducing electronics is to share or multiplex active tuning elements at the architecture level. Badal et al. [227] proposed a multimode OPA architecture, in which different guided modes generated multiple steered beams from a single silicon OPA, thereby eliminating the need to replicate independent OPAs for parallel beam formation. In the most compact configuration, shared multimode phase shifters reduced the device footprint by more than 30% and the total phase‐shifter power consumption by nearly 50% compared with a conventional multi‐OPA design, highlighting modal multiplexing as an effective route for reducing both the phase‐shifter count and the associated control hardware.

A third route is to compress the electrical addressing network itself. Hu et al. [228] proposed a row‐gated optical phased array (RG‐OPA) with on‐chip diodes to realize a row–column gating topology, reducing the required number of control electrodes from 32×8 to 32+8. This result shows that circuit‐topology innovation can substantially alleviate the scaling burden of large two‐dimensional OPAs without fully sacrificing beam‐steering functionality.

In parallel with these circuit‐ and architecture‐level strategies, recent advances in ${\text{LiNbO}}_{3}$‐based integrated optical phased arrays (OPAs) have shown that material‐embedded phase generation can provide another fundamentally different routes to reducing electronic control complexity. Unlike conventional OPAs, which typically rely on one phase shifter and one electrical control channel for each array element, cascaded domain engineering exploits the opposite electro‐optic responses of positive and negative ferroelectric domains to generate the required interelement phase progression under a single applied voltage. Specifically, the domain widths and interaction lengths are hierarchically arranged across multiple cascaded layers so that after light propagates through the entire structure, adjacent elements in the final layer acquire an equal phase difference suitable for beam steering. In this way, the phased‐array response can be governed by a single electrical control unit, whereas the number of effective emitters increases with the number of cascaded layers. In representative demonstrations, this material‐embedded phase‐generation strategy enabled continuous beam steering at modulation rates up to 3 MHz and beam divergence below 0.2°, while substantially reducing interconnect density, driver count, and associated power overhead [46, 119, 229], as shown in Figure 15c. These results highlight a fundamentally different route toward compact, low‐power, and scalable OPAs, in which part of the phase‐control burden is shifted from external electronics to the electro‐optic material structure itself.

Taken together, current efforts to reduce the electronic burden of large‐scale OPAs can be broadly grouped into three complementary directions: cascaded or hierarchical phase‐generation architectures, shared or multiplexed active‐tuning schemes, and electrical‐addressing‐network compression [223, 225, 226, 227, 228]. Cascaded control strategies, in particular, have emerged as a promising route by replacing element‐by‐element phase tuning with hierarchical or collective modulation schemes, thus substantially reducing the number of required interconnects and simplifying the driver architecture [223, 225, 226]. From early multistage addressing schemes to cascaded thermo‐optic phase shifters, directional‐coupler‐based distribution networks, and equal‐path‐length waveguide configurations, these approaches have progressively enabled beam steering with logarithmic or even single‐input control [223, 225, 226]. Although fabrication‐induced phase errors remain unavoidable, hybrid global‐local tuning schemes provide a possible route to restore phase fidelity [127]. More recently, material‐embedded phase‐generation concepts have further broadened this landscape by moving part of the phase‐control function from external electronics into the optical material structure itself [46, 212, 229]. Overall, these developments share a common objective: replacing element‐by‐element electronic control with more collective and distributed control paradigms, thereby enabling more scalable, compact, and energy‐efficient beam‐steering systems.

### 6.2 Crosstalk

In highly integrated on‐chip optical phased arrays (OPAs), achieving aliasing‐free wide‐field‐of‐view (FoV) beam steering generally requires subwavelength or half‐wavelength spacing between adjacent emitters. However, such compact geometries inevitably enhance evanescent coupling between neighboring waveguides, giving rise to pronounced optical crosstalk. This crosstalk manifests as phase errors, main‐lobe degradation, elevated sidelobe levels, and compromised far‐field beam quality and has, therefore, become a fundamental obstacle to high‐performance dense OPA integration. To address this issue, substantial efforts have been devoted to crosstalk mitigation through structural design, lattice and modal engineering, emitter‐level isolation, and algorithmic compensation.

A first class of strategies focuses on layout‐ and architecture‐level decoupling. From a structural design perspective, notable reductions in interchannel crosstalk have been achieved through nonuniform phased‐array layouts, angled free‐propagation regions (FPRs), deeply etched waveguides, and low‐loss mode converters. Experimental results across a range of channel spacings have demonstrated worst‐case crosstalk below −20 dB with insertion losses under 3 dB, highlighting the manufacturability and robustness of such approaches [232]. A more fundamental architectural route is to decouple the effective emitter pitch from the actual physical spacing between neighboring waveguides. In this context, a two‐layer OPA architecture with vertically staggered waveguides was proposed to preserve sufficiently large lateral and diagonal separations while still enabling a reduced effective pitch for beam steering. By introducing vertical layering and lateral offset simultaneously, this design suppresses interwaveguide coupling without sacrificing the small equivalent pitch required for wide‐angle operation. Both coupled‐mode analysis and full‐wave simulations confirmed that crosstalk can remain negligible even when the effective pitch is pushed into the subwavelength regime, highlighting array‐architecture redesign as a distinctive alternative to conventional waveguide‐shape optimization or nonuniform spacing.

A second class of strategies relies on lattice‐ and mode‐engineering concepts that suppress coupling by intentionally breaking local phase matching or strengthening modal isolation. To mitigate crosstalk in high‐density OPAs without compromising beam quality, waveguide superlattice engineering provides an effective route by introducing controlled mode mismatch between adjacent waveguides. By tailoring the propagation‐constant differentials within each supercell, this strategy breaks local phase‐matching conditions, suppresses evanescent coupling, and, at the same, time balances longitudinal coherence for low crosstalk with transverse coherence for proper beam formation. As shown in Figure 16a, the optimized SC5b superlattice exhibits the most favorable trade‐off, enabling a half‐wavelength‐pitch OPA with all crosstalk channels below −20 dB while maintaining low superlattice‐lobe levels and acceptable far‐field beam quality. These results suggest that crosstalk in integrated OPAs can be addressed not only by increasing element spacing but also by engineering the waveguide lattice itself to support dense integration and wide‐angle beam steering [152]. Beyond lattice redesign, enhanced mode confinement has also emerged as a promising direction. Novel configurations such as metamaterial‐cladded (e‐skid) waveguides [233, 234] and leaky subwavelength grating structures [231, 235] improve transverse optical confinement, achieving crosstalk levels below 30 dB at half‐wavelength pitch [236, 237, 238, 239, 240, 241].

**FIGURE 16:**
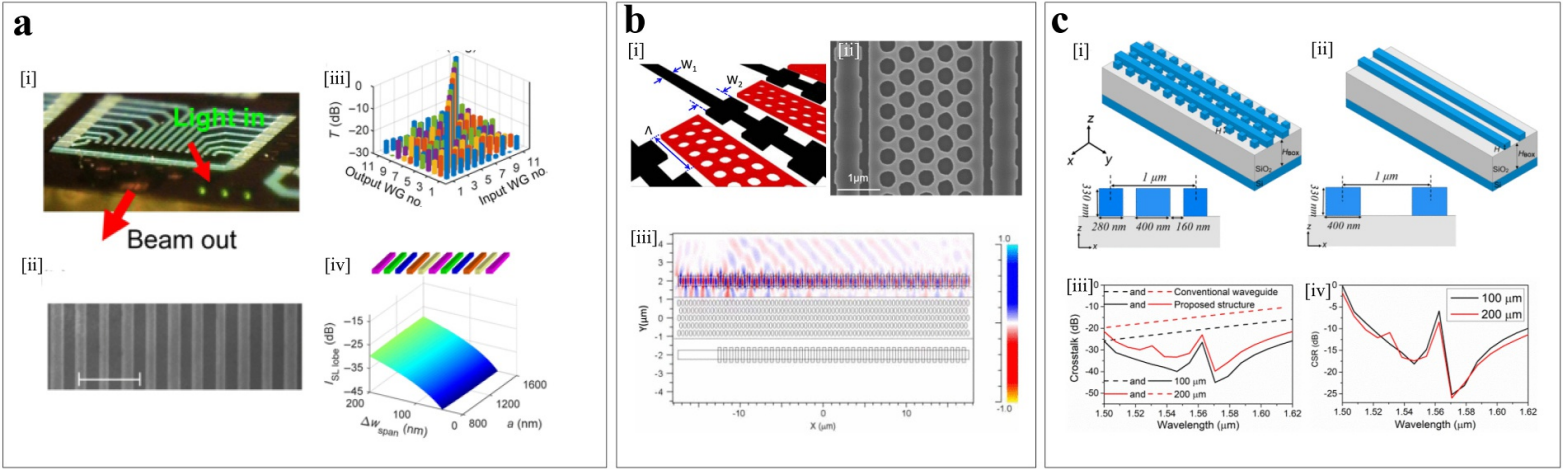
Representative strategies for suppressing crosstalk in optical phased arrays. (a) Waveguide‐superlattice‐based strategy for suppressing crosstalk in high‐density optical phased arrays. The four subpanels show [i] the fabricated OPA chip, [ii] the SEM image of the SC5b waveguide superlattice, [iii] the experimentally measured crosstalk spectra, and [iv] the calculated superlattice‐lobe characteristics, respectively. Compared with simpler superlattice configurations, the SC5b design provides a more favorable balance between low crosstalk and limited superlattice‐induced beam disturbance. The measured results confirm that all crosstalk channels remain below −20 dB, indicating that a half‐wavelength‐pitch OPA can be realized with suppressed interelement coupling while preserving beamforming capability [152]. (b) Crosstalk suppression in a corrugated‐waveguide optical phased array using a two‐dimensional photonic crystal. It combines [i] a close‐up of the corrugated waveguides with 2D PC isolation, [ii] the SEM image of the corrugated‐waveguide OPA with the 2D PC in between array elements, and [iii] a simulation of the same structure but with 5 periods of 2D PC between the two corrugated waveguides showing effective crosstalk suppression [230]. (c) Low‐crosstalk subwavelength‐pitch silicon waveguide design based on periodically loaded nanoblocks. It combines the three‐dimensional schematic views of [i] the proposed two‐waveguide structure with symmetrically arranged one‐dimensional silicon nanoblocks and [ii] the reference conventional coupled‐waveguide structure, together with [iii] the simulated crosstalk spectra and [iv] the corresponding coupling suppression ratio (CSR) for waveguide lengths of 100 μm and 200 μm. The periodic silicon nanoblocks act as resonant high‐reflection boundaries that truncate the evanescent fields responsible for interwaveguide coupling, thereby reducing crosstalk while still enabling weak‐radiating grating formation for a long effective emission length [231].

A third category targets crosstalk mitigation through codesign of the emitter structure itself. At the emitter level, crosstalk in high‐density OPAs can arise not only from conventional evanescent coupling between adjacent waveguides but also from grating‐assisted coupling induced by the radiating structure itself. Corrugated waveguides are attractive emitter designs because they enable weak free‐space radiation and long effective apertures with only a single patterning step. However, the lateral corrugations also open additional coupling channels between neighboring emitters, which become increasingly problematic as the element spacing is reduced for large‐angle beam steering. To suppress this parasitic coupling without sacrificing array density, a two‐dimensional photonic crystal (2D PC) slab can be inserted between adjacent corrugated waveguides. As shown in Figure 16b, the photonic bandgap of the 2D PC effectively blocks laterally coupled light in the operating wavelength range, thereby mitigating grating‐assisted coupling between emitters. Far‐field measurements further confirmed the effectiveness of this strategy: Without the 2D PC, the radiation pattern fragmented into multiple emission bands owing to supermode formation, whereas with the 2D PC, the far field was consolidated into a single dominant beam spot [230]. This result highlights an important crosstalk‐mitigation route for integrated OPAs, namely, that optical isolation structures can be codesigned with the emitter geometry itself to preserve dense element spacing while maintaining stable beam formation and steering capability. Periodic silicon nanoblock engineering provides another effective route to suppress crosstalk in subwavelength‐pitch OPAs while preserving long radiation apertures. By symmetrically introducing resonant nanoblocks alongside adjacent strip waveguides, the evanescent fields responsible for interwaveguide coupling can be truncated, thereby reducing crosstalk over a broad wavelength range. Meanwhile, the same periodic perturbation forms a weak‐radiating grating, enabling millimeter‐scale radiation lengths and ultranarrow beam widths [231]. As a result, this approach supports subwavelength‐pitch waveguide arrays with more than 10 dB crosstalk suppression, radiation lengths up to 1.47 mm, and a theoretical beam width of 0.052°, offering a promising platform for high‐performance two‐dimensional OPAs, as shown in Figure 16c. Because the structure remains symmetric, it also preserves compatibility with wavelength scanning, although the required nanoscale critical dimensions still present fabrication challenges for scalable implementation.

Beyond direct physical suppression of coupling, algorithmic compensation has emerged as a fourth route. In parallel, algorithmic methods such as predistortion of input optical fields and deep reinforcement learning‐based phase control have gained increasing attention. These techniques optimize the complex input amplitude at each emitter to compensate for fabrication‐induced imperfections and interelement coupling. An important extension of this direction is to compensate for crosstalk‐induced near‐field distortion rather than suppress coupling directly. Chen et al. [181] demonstrated a half‐wavelength‐pitch silicon OPA that combined independent amplitude and phase control with programmable beamforming, enabling aliasing‐free beam steering over 180°, a peak SLSR of about 24 dB, and an SLSR above 20 dB within ±60°. This result shows that crosstalk‐aware precompensation can transform dense OPAs from simple steering devices into more general programmable optical beamforming platforms. For example, a combined genetic and local‐search optimization applied to a 32‐channel, 1‐mm‐long array achieved an SLSR of 14.5 dB, with more than 50% of the total power concentrated in the main lobe [180, 242].

Taken together, crosstalk mitigation in integrated OPAs has evolved from simple geometric spacing control to a multilevel framework that includes architecture‐level decoupling, lattice and modal engineering, emitter‐level optical isolation, and algorithmic near‐field compensation. These complementary strategies do not merely suppress unwanted interchannel coupling; they increasingly enable dense integration, preserved beam quality, wide‐angle steering, and programmable beamforming under subwavelength‐pitch conditions. Such developments are expected to play a central role in next‐generation OPA chips that are ultradense, low‐power, wide‐angle, and dynamically reconfigurable.

### 6.3 Grating‐Lobe Suppression

Grating‐lobe suppression is a central requirement for optical phased arrays (OPAs) targeting wide‐field‐of‐view (FoV) beam steering and high angular resolution. In conventional uniformly spaced arrays, once the antenna pitch exceeds the half‐wavelength limit, multiple discrete grating lobes inevitably emerge, restricting the usable steering range and diverting optical power away from the main beam. To address this challenge, recent efforts have developed a broad range of suppression strategies, including array synthesis with nonuniform or sparse apertures, dense low‐crosstalk half‐wavelength arrays, aperture shaping and optical‐field filtering, and transceiver‐architecture‐based beam‐alignment schemes [247, 248, 249]. Together, these approaches have expanded grating‐lobe suppression from a simple pitch‐reduction problem into a broader framework of array and system codesign.

A first major class of approaches relies on array synthesis through nonuniform or sparse‐aperture engineering. Aperiodic array design has emerged as a powerful strategy by intentionally breaking the periodicity of the emitter arrangement, thereby disrupting the constructive interference condition at non‐main‐lobe directions and redistributing grating‐lobe energy into a broader sidelobe background. Although this process introduces an inherent beamforming‐efficiency penalty, it can substantially weaken aliasing and is particularly attractive for wide‐FoV and high‐resolution beam steering. Various design methodologies have been proposed for this purpose, including unequally spaced arrays, sparse‐sampling strategies, deterministic mathematical sequences, and optimization algorithms such as genetic and particle‐swarm methods. Recent studies further indicate that grating‐lobe suppression can be treated more generally as an array‐synthesis and topology‐engineering problem rather than solely a matter of pitch reduction. Gao et al. [250] combined a weakly perturbed evanescent‐field‐modulated grating antenna with a nonuniform sparse array to suppress grating lobes while enabling 120° lateral steering in a silicon OPA. Zang et al. and Yuan et al. [243, 251] further showed that optimization of nonuniform arrays can substantially improve beam quality at large steering angles by jointly considering multiangle sidelobe performance. Along a different direction, Lei et al. [252] demonstrated that circular array topologies can also mitigate grating lobes in sparse, large‐aperture OPAs by exploiting more favorable projection characteristics during beam steering. Despite the associated efficiency trade‐offs, these aperiodic and sparse architectures offer a viable path toward low‐sidelobe, grating‐lobe‐suppressed OPA systems with broad angular coverage.

For arrays whose antenna spacing cannot be reduced below the half‐wavelength limit, nonuniform array design provides an effective alternative to conventional uniform layouts. In a uniform OPA, once the element spacing exceeds λ/2, multiple discrete grating lobes inevitably appear. By contrast, a nonuniform OPA breaks the strict periodicity of the array and redistributes grating‐lobe energy into a broader and lower‐intensity sidelobe background, thereby significantly weakening the strong aliasing peaks characteristic of uniform arrays. However, for large‐angle beam steering, conventional optimization methods for nonuniform arrays often remain insufficient because the peak sidelobe level (PSLL) typically deteriorates as the steering angle increases. To address this issue, a dual‐adaptive genetic algorithm (DAGA) incorporating chaotic tent and logistic mappings was introduced to enhance the diversity of the initial population and jointly optimize array performance at both nonsteering and large steering angles. As shown in Figure 17a, this approach achieved significant PSLL reduction in 64‐, 128‐, and 256‐channel OPAs, indicating that nonuniform array engineering, when combined with globally effective optimization strategies, can provide a practical route to grating‐lobe suppression without requiring extreme pitch reduction [243].

**FIGURE 17:**
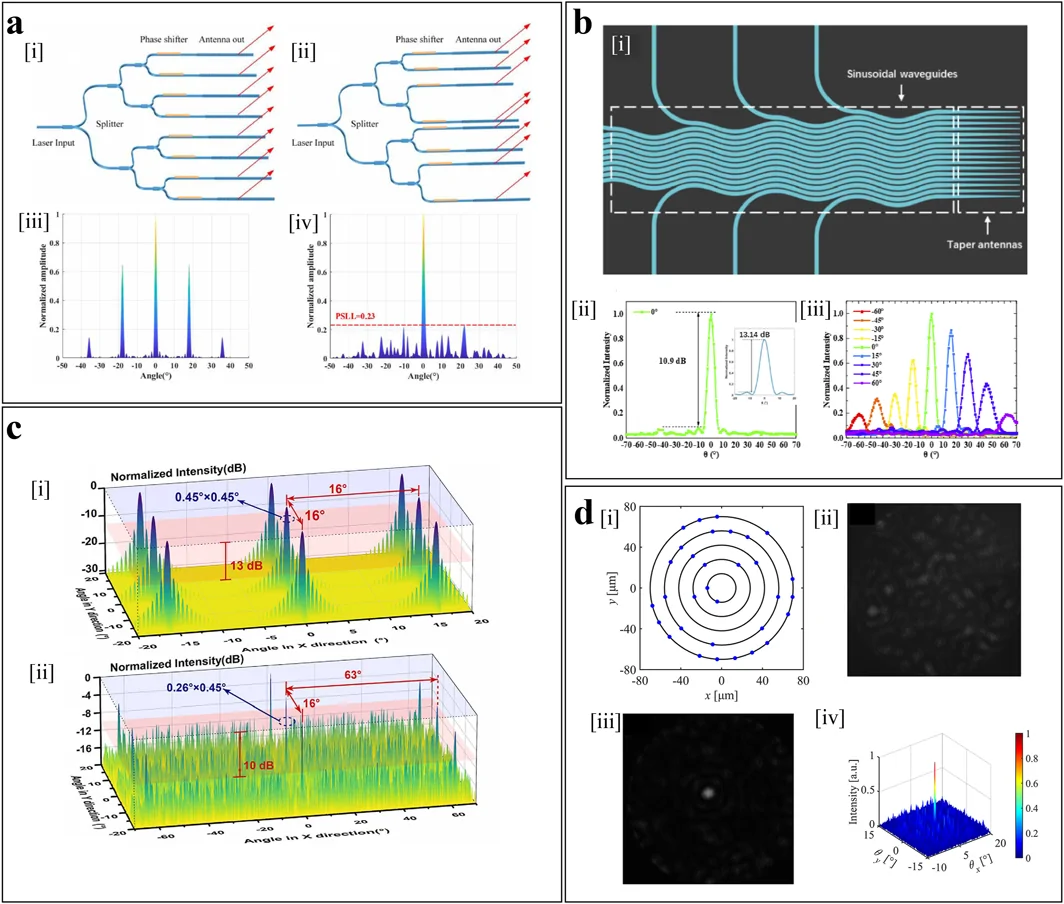
Representative schemes for suppressing grating lobes in optical phased arrays. (a) Grating‐lobe suppression by nonuniform optical phased‐array design. It combines the schematic comparison between [i] a uniform OPA and [ii] a nonuniform OPA, together with the corresponding far‐field two‐dimensional intensity distributions of [iii] the uniform array and [iv] the nonuniform array. In the uniform case, the periodic element spacing produces pronounced discrete grating lobes in the far field. By contrast, the nonuniform arrangement breaks the strict periodicity of the array and redistributes the grating‐lobe energy into a lower‐intensity sidelobe background, thereby suppressing strong aliasing peaks and improving the usable beam‐steering performance [243]. (b) Grating‐lobe‐free silicon optical phased array enabled by periodically bent dense antennas. It combines [i] the enlarged view of the output antenna array, where sinusoidally bent dense waveguides are integrated with tapered emitting antennas, [ii] the measured (green) and simulated (blue) normalized far‐field intensity profiles at 0°, and [iii] the measured far‐field beam distributions at different steering angles. The periodically bent waveguides suppress crosstalk in the dense array, allowing the antenna pitch to approach the grating‐lobe‐free regime, whereas the tapered antennas improve emission efficiency by reducing end‐face reflections. As a result, the fabricated device achieves beam steering over a 120° field of view without observable grating lobes [244]. (c) Grating‐lobe suppression in a two‐dimensional optical phased array using an aperiodic sparse grid. It combines [i] the simulated three‐dimensional far‐field pattern of a uniform full 2D OPA and [ii] the simulated three‐dimensional far‐field pattern of the optimized aperiodic sparse 2D OPA. Compared with the uniform array, the aperiodic sparse design breaks the strict periodicity of the lattice and redistributes grating‐lobe energy, thereby enlarging the grating‐lobe‐free steering range while maintaining a relatively narrow main beam [245]. (d) Grating‐lobe suppression in a two‐dimensional optical phased array using a circular sparse‐array aperture. It combines [i] the optimized 32‐element sparse distribution on a circular aperture, [ii] uncalibrated measurement results for a single steering angle of (5°, 0°), and [iii] calibrated far‐field observed images, and [iv] measured 2D far‐field intensity pattern in a 30° × 30° FoV. By sparsifying the circular aperture and breaking the regular spatial symmetry of the initial array, the design suppresses the grating‐lobe background in the two‐dimensional field of view while preserving a clear main beam and a relatively narrow far‐field spot. This architecture illustrates an effective aperture‐level strategy for grating‐lobe suppression in compact 2D silicon OPAs [122].

For two‐dimensional OPAs, grating‐lobe suppression through sparse and nonperiodic aperture engineering becomes even more important because the array pitch is constrained simultaneously by optical crosstalk, waveguide routing, and antenna footprint. As shown in Figure 17c, one effective alternative to uniformly populated arrays is to combine aperiodization with sparsification. In this strategy, a full two‐dimensional array is first mapped onto an aperiodic grid with optimized interelement spacing, and then only a subset of grid points is occupied by grating antennas to form a sparse array. Such a design breaks the strict translational symmetry of the conventional periodic lattice and redistributes grating‐lobe energy, thereby substantially enlarging the grating‐lobe‐free steering range [245]. The simulated far‐field patterns show that, compared with a uniform full 2D OPA, the optimized aperiodic sparse array dramatically broadens the grating‐lobe‐free steering range in the X direction while preserving a relatively narrow beam divergence [245]. Although this approach leads to some degradation in the sidelobe suppression ratio, it provides a practical route toward enlarging the usable steering range of 2D OPAs without requiring a fully populated dense array. Grating‐lobe suppression in two‐dimensional OPAs can also be realized through circular sparse‐aperture engineering rather than relying solely on reducing the antenna pitch [248, 253]. As shown in Figure 17d, a circular sparse‐array aperture was introduced to break the regular spatial periodicity of the antenna layout and thereby suppress grating lobes over the two‐dimensional field of view [122]. Starting from a multiring circular arrangement, only a subset of antenna elements was retained after optimization so that the resulting sparse distribution reduced the grating‐lobe background while preserving a compact aperture and a relatively narrow main beam. The measured far‐field results after phase calibration further confirmed that this strategy can support stable beam formation at different steering angles, with a clear main lobe and acceptable sidelobe suppression in the scanned region [122]. Compared with conventional uniformly distributed apertures, this approach is especially attractive for compact 2D OPAs because it reduces both the strength of parasitic lobes and the number of required active channels.

A second class of strategies suppresses grating lobes by maintaining the antenna pitch near the half‐wavelength regime while reducing interelement crosstalk sufficiently to preserve dense integration. A direct route to grating‐lobe suppression is, therefore, to reduce the antenna pitch to the half‐wavelength regime, provided that crosstalk in the dense array can be kept sufficiently low. Along this direction, a grating‐lobe‐free silicon OPA based on sinusoidally curved dense waveguides and tapered output antennas demonstrated a 120° field‐of‐view beam steering without observable grating lobes [244], as shown in Figure 17b. In this architecture, the periodic bending of the waveguides suppresses crosstalk between adjacent dense channels, whereas the tapered antennas reduce end‐face reflections and improve emission efficiency. The measured far‐field profile agrees well with the simulation, confirming that dense low‐crosstalk waveguide engineering can provide a practical route to grating‐lobe suppression without resorting to aperiodic emitter spacing or sidelobe redistribution. In the context of multibeam OPAs, grating‐lobe interference has also been mitigated by integrating particle‐swarm optimization (PSO) into the array‐division multiplexing (ADM) of unequally spaced antennas, reducing sidelobe energy from 38% to 5% during simultaneous multitarget scanning [254]. Furthermore, large‐scale sparse aperiodic arrays have shown that comparable beamforming performance can be achieved with significantly fewer antenna elements. A 192‐channel implementation achieved grating‐lobe‐free ±90° steering while reducing the antenna count by more than sixfold, thereby lowering system complexity and power consumption [255].

A third route focuses on aperture shaping and optical‐field filtering. Several studies have explored hybrid strategies that combine physical design with intelligent optimization, such as genetic algorithms, PSO, and convex optimization (CVX), achieving sidelobe suppression better than −24 dB while balancing complexity, steering range, and main‐lobe power efficiency [256]. Another important route is amplitude apodization, which improves far‐field quality by tailoring the aperture power distribution. Liu et al. [257] implemented a Taylor amplitude distribution in a TFLN–SiN OPA and achieved significantly improved sidelobe suppression across the field of view, confirming the effectiveness of aperture shaping for low‐sidelobe beam steering. A more system‐level example was provided by Yang et al. [258], who developed a Vernier OPA transceiver on a SiN‐on‐SOI platform for LiDAR applications. By combining mismatched TX/RX channel spacing with quasi‐Gaussian amplitude apodization, the design spatially separated grating lobes while reinforcing the main lobe, showing that aperture engineering remains effective even when grating lobes cannot be eliminated purely through geometric scaling. At the antenna‐element level, near‐wavelength high‐contrast gratings (HCGs) have also been used as angularly selective matching layers, providing up to 10 dB grating‐lobe suppression by reflecting high‐angle non‐main‐lobe radiation while preserving main‐lobe integrity [259]. Beyond array synthesis and amplitude shaping, grating‐lobe suppression can also be addressed through optical‐field filtering. This approach is particularly valuable for visible‐light OPAs, where the λ/2 pitch required for aliasing‐free steering is often difficult to achieve. Xue et al. [260] introduced a slab‐assisted OPA architecture in which a free‐propagation‐region slab acted as a spatial filter to remove unwanted lobes before final outcoupling, indicating that grating‐lobe suppression can also be realized through on‐chip manipulation of the emitted optical field.

Beyond these array‐synthesis‐ and aperture‐shaping‐based methods, a distinct system‐level suppression paradigm has emerged through transmit/receive architectural codesign. To transcend the inherent trade‐off between grating‐lobe suppression and field‐of‐view extension, the bistatic Vernier array (BVA) has been proposed. This architecture introduces a slight mismatch in element pitch between the transmitter (TX) and receiver (RX) OPAs, creating a Vernier angular‐period offset that decouples the angular positions of grating lobes between the two arrays. As a result, only the desired beam direction remains spatially aligned in the synthesized response, whereas nontarget grating lobes are spatially misaligned and suppressed. Compared with aperiodic arrays, which typically disperse grating‐lobe energy into a sidelobe floor, the BVA concept preserves periodicity and confines grating‐lobe energy to predetermined nonoverlapping directions, thereby retaining more useful beam power. In addition, the bistatic TX/RX separation improves tolerance to residual reflections in practical photonic integrated circuits.

Since its proposal, the BVA concept has been experimentally validated with promising results. In one implementation, a wavelength‐assisted grating‐based BVA FMCW LiDAR developed at Jilin University employed 128‐channel OPAs with TX and RX element pitches of 3.8 μm and 4.2 μm, respectively, achieving SMSRs of 10.4–25.3 dB and background signal‐to‐noise ratios of 7.3–20 dB while enabling three‐dimensional point‐cloud reconstruction over a 135°–160° angular range [146]. As shown in Figure 18a, these performances were enabled by jointly engineering the array architecture and the antenna radiation envelope. Specifically, a Vernier OPA transceiver was used to suppress aliasing by aligning only the target main lobes of the transmit and receive OPAs while keeping the remaining grating lobes spatially misaligned, whereas a chained grating antenna was introduced to broaden the single‐antenna far‐field envelope and maintain a nearly flat beam‐power profile over an ultrawide angular range. In this way, the OPA‐based FMCW LiDAR preserved sufficient beam quality and signal‐to‐noise performance even near the edge of the field of view. The measured point‐cloud image further verified wide‐scene three‐dimensional imaging over more than 135°, with the reported horizontal imaging field of view extendable to 160°. This work, therefore, highlights an important route toward grating‐lobe suppression in integrated OPAs, where sidelobe suppression and field‐of‐view extension are addressed in a unified manner through the codesign of the array architecture and antenna emission profile.

**FIGURE 18:**
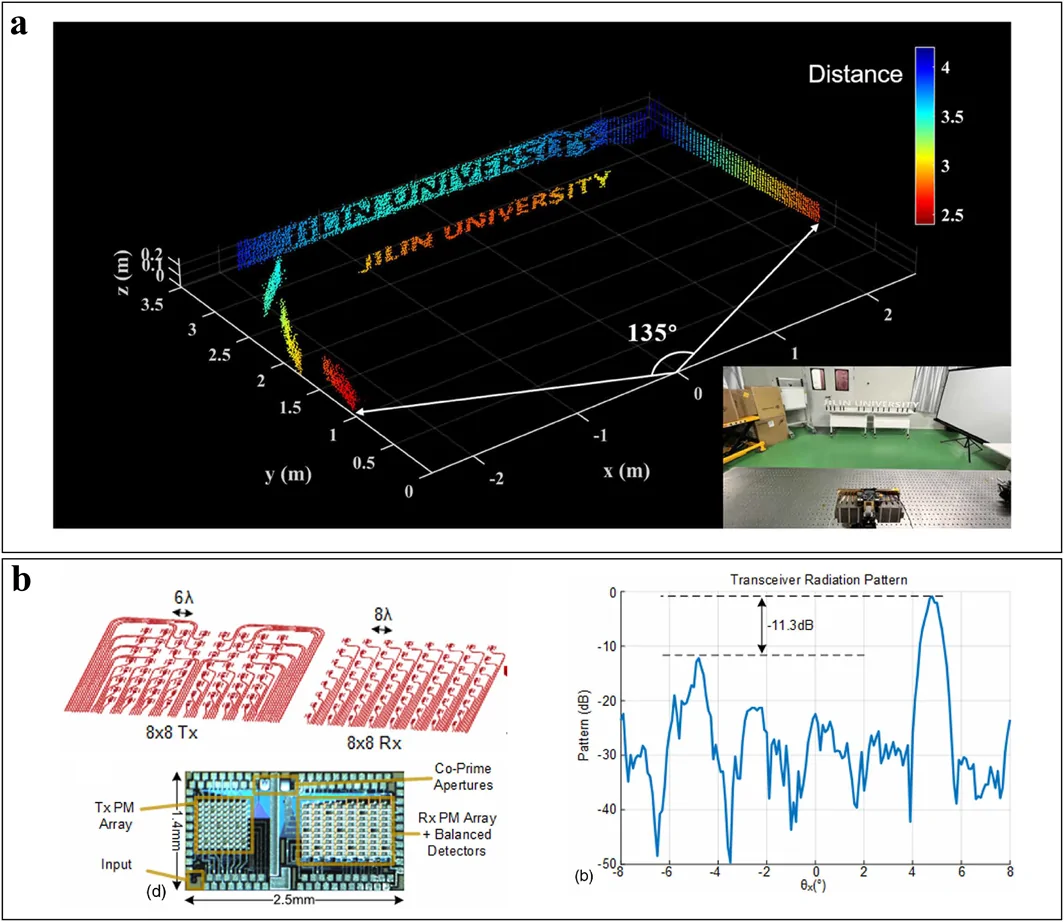
Simulation and demonstrations of bistatic Vernier arrays. (a) Wide‐scene three‐dimensional imaging enabled by a grating‐lobe‐suppressed Vernier optical phased array. It shows the measured three‐dimensional point cloud of a wide‐scene target over a field of view exceeding 135°, where the inset is the corresponding camera image of the scene. By combining the Vernier OPA architecture for aliasing suppression with chained grating antennas for a broad and flat far‐field envelope, the system achieves ultrawide‐angle beam steering and high‐quality point‐cloud reconstruction with minimal blind spots. This result illustrates the system‐level impact of grating‐lobe suppression for OPA‐based LiDAR [146]. (b) Grating‐lobe suppression using a co‐prime photonic beamforming transceiver. It combines the implemented co‐prime transmitter and receiver apertures with different element spacings, the equivalent half‐wavelength uniform array used as a beamwidth reference, and the synthesized transceiver radiation pattern. Although the transmitter and receiver apertures individually exhibit grating lobes, their co‐prime element spacings ensure that only the target beam direction overlaps in the final transceiver response, whereas the remaining grating lobes are spatially misaligned and suppressed. This architecture enables grating‐lobe‐free beamforming with substantially reduced array complexity [246].

This system‐level beam‐alignment philosophy can be generalized beyond Vernier architectures. Although beam‐alignment‐based schemes generally incur a reported main‐lobe power loss of 8.33–20 dB [146, 246, 261], they retain grating‐lobe energy for useful beamforming rather than dissipating it into an incoherent sidelobe floor, which makes them fundamentally different from aperiodic arrays. As shown in Figure 18b, a representative implementation is the coprime transceiver architecture, in which transmitter and receiver apertures with different co‐prime element spacings are codesigned so that only the target beam direction overlaps in the synthesized response, whereas the remaining grating lobes are spatially misaligned and suppressed [246]. In this way, grating‐lobe‐free beamforming can be achieved with far fewer radiating elements and lower photonic/electronic complexity than an equivalent densely filled half‐wavelength array while experimentally maintaining a sidelobe level of about −11.3 dB over the full scan range [246].

Taken together, grating‐lobe suppression in integrated OPAs has evolved far beyond simple pitch reduction. Current solutions can be broadly grouped into array synthesis and sparse‐aperture engineering, dense low‐crosstalk half‐wavelength arrays, aperture shaping and optical‐field filtering, and transceiver‐architecture‐based beam alignment. These complementary strategies collectively expand the grating‐lobe‐free steering range, improve main‐lobe quality, reduce parasitic beam formation, and support wider‐angle and more power‐efficient beam steering. As a result, grating‐lobe suppression is increasingly becoming a problem of coordinated array, aperture, emitter, and system codesign rather than a single geometric constraint.

### 6.4 System‐Level Challenges: Optical Loss, Main‐Lobe Emission Efficiency, and Field of View

In large‐scale integrated optical phased arrays (OPAs), optical loss, main‐lobe emission efficiency, and field of view (FoV) are not independent performance metrics but strongly coupled system‐level constraints that jointly determine how much optical power can ultimately be delivered into the desired free‐space beam [77, 262]. Their interplay fundamentally limits the scalability, efficiency, and practical applicability of OPAs in LiDAR and free‐space systems. In addition, these trade‐offs are further complicated by fabrication variability and robustness constraints, which increasingly dominate performance in large‐scale arrays. As a result, system‐level OPA optimization must be understood not as the isolated improvement of a single metric but as a coordinated balance among power budget, beam concentration, practical FoV, and manufacturability.

A first limitation arises from the on‐chip optical power budget, which is directly constrained by propagation loss, coupling loss, insertion loss, and nonlinear power‐handling limits. In III/V‐on‐SOI platforms, although heterogeneous integration enables on‐chip amplification and lasing to compensate for input coupling loss and waveguide attenuation, residual *evanescent coupling loss* between III/V and silicon waveguides, fabrication‐induced parasitic loss, and limited *plug‐to‐wall efficiency* persist [263, 264]. The low electrical‐to‐optical efficiency (approximately 10%) of III/V amplifiers leads to substantial thermal accumulation and *thermally induced gain degradation*, as experimentally observed in arrayed OPAs with performance degradation exceeding 3.5 dB due to thermal crosstalk [265]. Even at the phase‐control level, III/V electro‐optic phase shifters introduce non‐negligible insertion loss, which accumulates across large arrays, although it remains lower than that of silicon free‐carrier‐based phase shifters [27, 266, 267, 268, 269]. Multilayer SiN‐on‐SOI platforms alleviate the nonlinear loss and power‐handling limitations of silicon bus waveguides, enabling watt‐level to tens‐of‐watts continuous‐wave throughput [76, 200, 206]. However, the weaker optical confinement of SiN waveguides introduces new loss mechanisms. Pure SiN grating antennas are more susceptible to optical crosstalk, leading to beamforming losses exceeding 10 dB in many demonstrations [70, 72, 74, 149]. In addition, cascaded interlayer coupling in Si–SiN architectures, even with high‐yield evanescent couplers, introduces cumulative insertion loss that becomes significant in large‐scale routing networks [163]. Another system‐level constraint is power handling under high optical intensity. Chen et al. [270] addressed this issue using a thick‐silicon SOI platform, where increased silicon thickness and waveguide cross‐section reduced optical power density and mitigated nonlinear losses associated with two‐photon absorption and free‐carrier absorption. Their demonstration of a two‐dimensional 64‐element OPA highlights that propagation efficiency and power tolerance are central considerations in high‐power OPA systems.

Beyond the available power budget, a second system‐level bottleneck is how efficiently the remaining optical power can be concentrated into the desired main beam. In practical OPAs, a substantial fraction of the radiated power is diverted into grating lobes, sidelobes, substrate leakage, or parasitic Fabry–Pérot interference [271, 272]. Experimental measurements on near‐field‐focusing OPAs indicate that, after accounting for waveguide loss, power‐divider loss, antenna inefficiency, and grating‐lobe leakage, the net on‐chip‐to‐main‐lobe efficiency can be as low as −12 to −18.5 dB [272]. These effects are particularly pronounced in arrays with super‐Nyquist element spacing, nonideal amplitude tapering, and waveguide‐grating antennas exhibiting intrinsic up/down radiation symmetry, even when upward emission efficiency is locally improved using distributed Bragg reflectors or metallic mirrors [271]. A complementary route is to improve the effective aperture and main‐lobe concentration through coherent synthesis of multiple arrays. Yan et al. [273] demonstrated the coherent combination of two silicon OPAs using off‐chip phase locking and piston‐phase stabilization, achieving a 319% increase in far‐field main‐lobe peak intensity together with a 62% reduction in main‐lobe width relative to a single OPA. This result highlights coherent aperture synthesis as an effective strategy for overcoming the output‐power and aperture limits of individual integrated OPAs.

A third major constraint is the practically usable field of view, which is fundamentally coupled to both optical loss and main‐lobe efficiency. In electronically steered OPAs, the maximum steering angle is limited by array pitch because super‐Nyquist spacing inevitably produces grating lobes whose intensity can approach that of the main lobe, thereby restricting the usable FoV [69, 162]. Although half‐wavelength element spacing can suppress grating lobes in principle, it is difficult to implement in large‐scale photonic integrated circuits because of submicron fabrication constraints, increased crosstalk, and routing complexity [162]. In wavelength‐assisted steering schemes based on waveguide‐grating antennas, the FoV is additionally constrained by the wavelength‐to‐angle tuning efficiency and available laser bandwidth, with large steering angles often accompanied by reduced radiation efficiency and elevated sidelobe levels [274]. At the same time, antenna radiation directionality deteriorates at large angles, increasing substrate leakage and angle‐dependent loss, which further narrows the practical FoV [272]. To partially recover the practical FoV constrained by these effects, recent studies have increasingly turned to system‐level codesign rather than pitch scaling alone. Kim et al. [275] improved longitudinal steering efficiency in thermo‐optically tuned silicon OPAs through backside air‐trench thermal isolation. Zhao et al. [276] enhanced the vertical FoV of wavelength‐steered OPAs through polarization multiplexing, whereas Guo et al. [125] synthesized four partially overlapping subfields into a continuous 360° lateral scan range using a multibeam top‐facing silicon OPA. Liu and Hu [277] further showed that slab‐grating beamforming can jointly support ultrawide two‐dimensional steering and high main‐lobe efficiency. Taken together, these results indicate that practical FoV scaling increasingly depends on thermal engineering, polarization multiplexing, optical‐field synthesis, and subfield stitching.

Beyond nominal optical performance, manufacturability and robustness are also major system‐level constraints for large‐scale OPAs. Zhang et al. [278] showed that intrawafer spatial variation, layout pattern‐density mismatch, and line‐edge roughness can substantially degrade the far‐field beam quality of splitter‐tree‐based OPAs by disturbing phase and amplitude balance. Their results emphasize that fabrication tolerance and process robustness must be considered alongside optical efficiency and FoV in practical OPA system design.

These coupled effects reveal a fundamental system‐level trade‐off: Extending the FoV often intensifies optical loss and reduces main‐lobe emission efficiency, whereas aggressive grating‐lobe and sidelobe suppression frequently requires denser apertures and stricter fabrication tolerances that further increase loss and complexity [77, 255]. Consequently, high‐performance OPAs cannot be realized through the isolated optimization of individual components. Instead, they require holistic co‐optimization of array geometry, antenna radiation engineering, power distribution networks, thermal management, phase‐calibration strategies, and fabrication robustness in order to simultaneously approach wide FoV, high main‐lobe efficiency, acceptable optical loss, and scalable manufacturability in large‐scale integrated systems.

### 6.5 Chip–Package Codesign and Electronic–Photonic Cointegration

Electronic driving and control architecture play a decisive role in determining the scalability, update rate, and system‐level power consumption of optical phased arrays (OPAs). Early OPA demonstrations predominantly relied on off‐chip electronics—including external DACs, FPGA controllers, and benchtop drivers—to address and tune individual phase shifters. Such configurations offer maximal flexibility in voltage swing, reconfigurable control logic, and thermal management and are, therefore, well suited to device characterization and algorithm development. However, as the aperture scales, off‐chip architectures face severe practical bottlenecks: The number of I/O channels and interconnects grows roughly linearly with the element count; long traces, cables, and wire bonds introduce parasitic resistance, inductance, and capacitance that degrade signal integrity; and the achievable phase‐update rate is often constrained to the kHz regime in large‐channel systems. Together, these factors increase total power consumption, complicate calibration, and lead to bulky form factors that hinder deployment in size‐ and power‐constrained applications such as LiDAR, AR/VR, robotics, and mobile platforms. As a result, as OPA arrays scale toward hundreds or thousands of emitters, the dominant bottleneck increasingly shifts from photonic device feasibility to electronic routing density, driver placement, sensing infrastructure, and electronic–photonic co‐optimization.

To address these limitations, recent efforts have increasingly moved toward on‐chip, near‐chip, or co‐packaged driver electronics, in which CMOS drivers, multiplexed DACs, or row–column addressing networks are integrated on the same die, on a closely coupled electronic die, or within the package. By shortening interconnect length and reducing parasitic loading, such architectures can substantially extend the achievable electrical bandwidth and phase‐update capability while reducing the energy and area cost per controlled channel. A closely related direction is multilayer photonic integration for high‐density OPA layouts. Zhang et al. [279] proposed a double‐layer silicon OPA transceiver in which routing and part of the phase control were assigned to a lower layer, whereas the antenna array and in‐aperture phase shifters were arranged in an upper layer. By combining vertical functional partitioning with interlayer coupling, the design achieved a 5‐μm antenna pitch while preserving independent phase control, highlighting vertical integration as a practical route toward compact, high‐density two‐dimensional beam‐steering architectures. Beyond routing‐density relief, tighter integration also opens new possibilities for embedded monitoring and closed‐loop stabilization. On‐chip photodiodes or in situ sensors can support automated phase stabilization and drift compensation that are difficult to scale with purely off‐chip hardware. Beyond on‐chip sensing, package‐level monitoring is also becoming increasingly important. Jin et al. [280] demonstrated a packaged OPA system in which a single photodetector mounted above the chip monitored lens‐reflected light to retrieve channelwise phase errors, thereby enabling in situ beamforming without external far‐field cameras or complex on‐chip monitoring networks. This work shows that packaging is no longer merely a passive optical and electrical interface but an active part of the beamforming, monitoring, and calibration architecture. Nevertheless, tighter electronic–photonic integration introduces new constraints, including higher thermal density, limited voltage headroom in advanced CMOS nodes, and increased process complexity associated with heterogeneous integration across Si, SiN, and LNOI platforms. These trade‐offs motivate system‐level codesign spanning packaging, power delivery, thermal management, sensor placement, and driver architecture [281].

Although co‐packaged optics (CPO) was originally driven by datacenter interconnects, several integration concepts developed in the CPO literature are directly relevant to large‐format OPAs because the two classes of systems face closely related bottlenecks in routing density, thermal drift, device variability, monitoring, and feedback control [282, 283, 284, 285]. Microring modulator (MRR) transmitter arrays, for example, constitute deeply cointegrated PIC–CMOS systems that must contend with thermal drift, device‐to‐device variability, multiphysics electro‐optic‐thermal coupling, and closed‐loop stabilization—challenges that closely parallel those of dense OPA arrays [286]. As emphasized in the CPO literature [287], MRR systems rely on continuous monitoring and adaptive control using on‐chip photodiodes [288], CLIPP sensors [289], and feedback algorithms [288, 290, 291, 292, 293, 294, 295, 296]. In the context of OPAs, these methods motivate persistent phase calibration, real‐time drift compensation, and sensor‐assisted stabilization to counter fabrication nonuniformity, thermal gradients, and electrical noise. Likewise, heater‐induced thermal crosstalk and MIMO‐style compensation methods developed for MRR arrays provide a useful blueprint for high‐density thermo‐optic phase‐shifter networks, in which tuning one element perturbs its neighbors and requires matrix‐based thermal compensation together with electro‐optic‐thermal cosimulation.

Complementary guidance arises from Mach–Zehnder modulator (MZM) transmitter arrays, where driver architecture determines achievable bandwidth, energy efficiency, and voltage swing. Techniques such as stacked‐FET output stages [297, 298, 299], traveling‐wave electrodes [300], distributed amplification [301, 302, 303, 304], and bandwidth‐extension networks—originally engineered for high‐speed MZM signaling—highlight packaging‐relevant design principles that are equally important for EO OPA arrays: delivering sufficient phase excursion under limited voltage headroom, maintaining wide electrical bandwidth over distributed electrodes, and mitigating parasitic loading introduced by routing and packaging. In this sense, the CPO literature provides not only useful analogies but also a directly relevant methodology for electronic–photonic codesign in scalable OPA systems.

At the subsystem level, these ideas are already beginning to converge in integrated beam‐steering engines. A representative example was reported by Lukashchuk et al., who demonstrated a photonic–electronic integrated LiDAR engine consisting of a hybrid Vernier laser, a high‐voltage arbitrary‐waveform‐generator ASIC, and an on‐chip erbium‐doped waveguide amplifier [305]. Implemented by combining III–V gain chips, ${\text{Si}}_{3}N_{4}$ photonic integrated circuits, and SiGe BiCMOS electronics, this system illustrates how compact source, driver, and amplification modules can be cointegrated to replace bulky external components in practical OPA‐based LiDAR engines. More broadly, as OPAs scale toward kilopixel‐class apertures, the dominant challenges increasingly shift from the feasibility of individual photonic devices to the co‐optimization of routing density, power delivery, thermal floorplanning, monitoring infrastructure, and driver placement. These are precisely the domains in which chip–package codesign and electronic–photonic cointegration become enabling, rather than merely supportive, technologies.

In summary, off‐chip electronics remain indispensable for early‐stage prototyping because of their flexibility and ease of access, but they scale poorly in terms of interconnect complexity, signal integrity, and system footprint. By contrast, on‐chip, near‐chip, and CPO‐inspired cointegrated architectures provide a practical route toward the integration density, bandwidth, stability, and energy efficiency required for large‐format OPAs. As the field advances toward hundreds to thousands of emitters, chip–package codesign and electronic–photonic cointegration are likely to become primary enablers of manufacturable, compact, and high‐performance beam‐steering systems.

## 7 Applications and Future Outlook

### 7.1 Established and Near‐Term Applications

On‐chip optical phased arrays (OPAs) are rapidly emerging as transformative platforms across multiple application domains, as shown in Figure 19, with LiDAR representing the most technologically mature and commercially visible direction. By replacing mechanically rotating components with digitally programmable beam steering, OPAs enable inertia‐free operation, compact solid‐state integration, and high‐resolution three‐dimensional sensing. Their ability to combine beam steering, calibration, and control within a photonic integrated platform offers clear advantages in robustness, size, and system complexity, making them particularly attractive for autonomous driving, advanced driver‐assistance systems (ADAS), robotics, and drone sensing. Although widespread deployment still depends on overcoming challenges such as high‐yield fabrication of large‐scale arrays, phase‐uniform control, crosstalk suppression, and the cointegration of high‐power light sources and low‐noise detectors, OPAs are widely regarded as a strong candidate for near‐term mid‐range, high‐resolution LiDAR and longer‐term fully solid‐state automotive perception systems.

**FIGURE 19:**
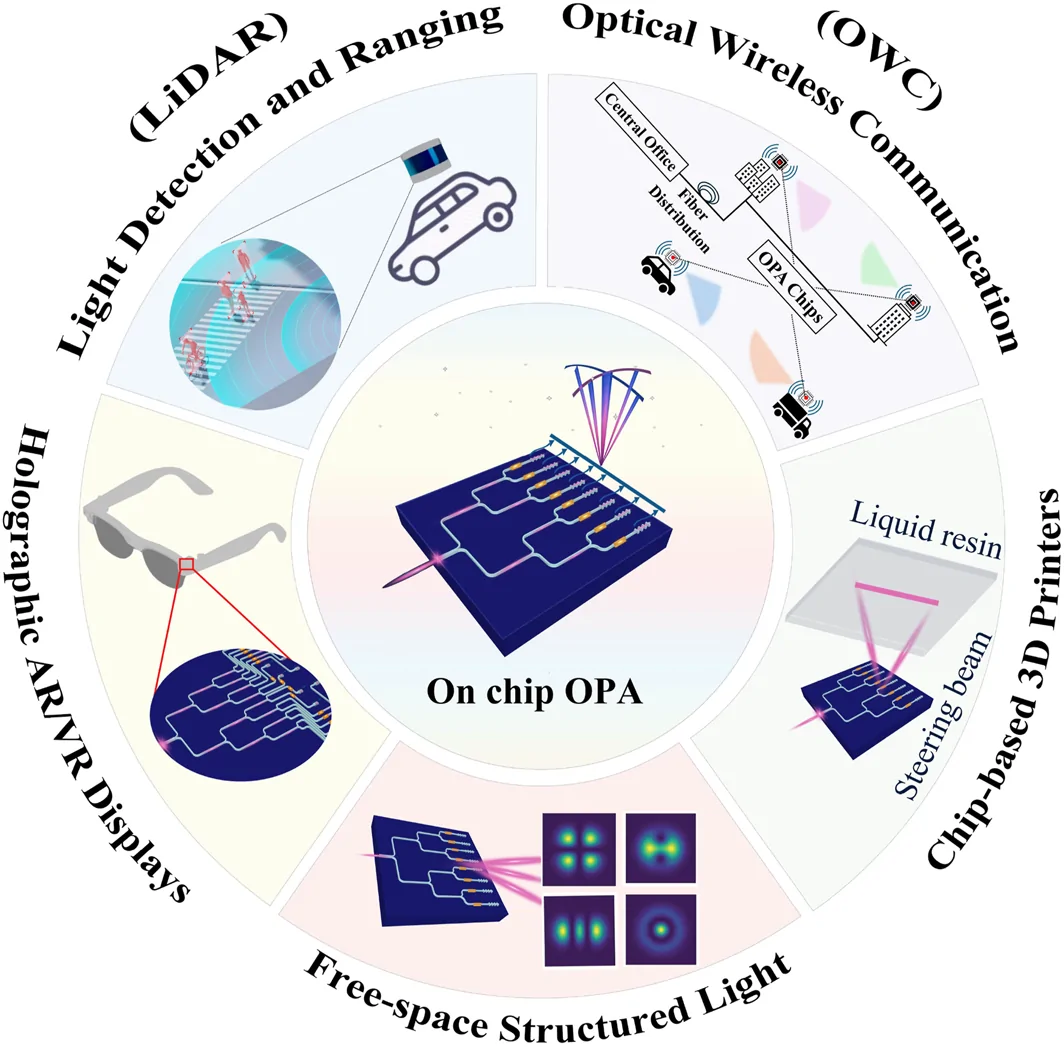
Applications of on‐chip optical phased arrays in multiple technological domains.

Among the various application directions of on‐chip OPAs, LiDAR has become one of the most active and technologically mature. Early studies mainly established the core capabilities required for solid‐state ranging, including beam steering, multibeam generation, and angle estimation. For example, Yang et al. [258] demonstrated a Vernier OPA transceiver on a SiN‐on‐SOI platform, where mismatched channel spacing between the transmitting and receiving arrays, together with amplitude apodization, enabled wide‐field beam steering with strong sidelobe suppression for FMCW LiDAR. Zhou et al. [306] explored a Butler‐matrix‐based shared‐aperture architecture for wavelength‐assisted two‐dimensional multibeam steering and parallel ranging, indicating a practical route toward higher sampling efficiency with reduced system complexity. Yuan et al. [307] further showed that angle‐estimation accuracy can also be substantially improved at the algorithmic level through difference‐beam comparison.

More recent progress has pushed OPA‐based LiDAR from beam‐steering demonstrations toward increasingly complete system implementations. Jin et al. [308] developed a coherent line‐beam FMCW LiDAR based on a hybrid ${\text{Si}}_{3}N_{4}$–polymer OPA, showing that line‐beam operation can simplify scanning while preserving simultaneous distance and velocity measurement. Li et al. [309] advanced this direction with a Si–SiN focal‐plane switch array chip integrating beam emission, steering, and coherent reception for high‐precision ranging together with 3D and 4D point‐cloud imaging. Lee et al. [310] demonstrated a monolithically integrated polymer–SiN OPA LiDAR system based on line‐beam scanning, highlighting a practical balance among optical throughput, power efficiency, and solid‐state imaging capability. Zhang et al. [311] introduced a phase‐modulated continuous‐wave (PhMCW) OPA LiDAR architecture as an alternative to both ToF and conventional FMCW schemes, whereas Okano et al. [312] showed that fast‐axis OPA steering combined with wavelength‐dispersive slow‐axis scanning can support compact swept‐source FMCW LiDAR with full‐frame 3D imaging. Taken together, these studies indicate that OPA‐based LiDAR is maturing from isolated beam‐steering hardware into increasingly complete sensing architectures.

As these systems move closer to deployment, calibration and control are becoming as important as the optical hardware itself. Deterministic pointwise optimization has been used to compensate for random initial phase errors in ${\text{LiNbO}}_{3}$‐based OPAs and improve both diffraction efficiency and sidelobe suppression [313]. For larger arrays, Gao et al. [314] proposed a physics‐informed neural‐network calibration framework for a 128‐channel silicon OPA, reducing dependence on measured data while improving calibration accuracy. Sved et al. [315] further showed that machine learning‐assisted control can directly infer phase‐driving signals from far‐field responses, enabling more efficient compensation for fabrication errors, wavelength‐dependent variations, and thermal crosstalk. These developments suggest that practical OPA LiDAR will increasingly rely on the codevelopment of system architecture, signal processing, calibration, and intelligent control.

Beyond laboratory demonstrations, the commercialization of OPA‐based sensing is also beginning to provide useful system‐level guidance. Commercial efforts based on CMOS‐compatible silicon photonics indicate that large, highly uniform emitter arrays, efficient high‐speed phase shifters, and tight electronic–photonic cointegration are likely to be central requirements for scalable OPA productization. More broadly, these developments suggest that practical OPA deployment will depend not only on photonic‐device performance but also on manufacturing yield, packaging, calibration stability, and compact system integration.

In addition to LiDAR, OPAs are increasingly being explored for free‐space optical communication (FSO) and related wireless links. Early studies mainly established OPA‐based beam steering as an enabling function for directional communication [316, 317, 318, 319, 320, 321, 322, 323, 324]. Ren et al. [325], for example, demonstrated an 8×8 matrix‐vector optical phased array for one‐to‐multipoint laser communication, achieving four‐channel transmission over an equivalent distance of 100 km with an aggregate capacity of 40 Gbit/s. In a related hybrid photonic–wireless configuration, Kamiura et al. [326] combined an OPA with a UTC‐PD array to realize beam‐steerable 300‐GHz terahertz wireless communication, illustrating the use of OPAs as optical‐domain phase‐control units for photonic beamforming.

Beyond directional link establishment, OPAs have also been exploited to improve link robustness and spatial scalability. Balasiano et al. [327] demonstrated a 10‐km atmospheric FSO system using OPA‐assisted coherent beam combining, where turbulence‐induced beam wander and scintillation were mitigated, whereas data rates of up to 100 Gbit/s were maintained. Wang et al. [328] further extended OPA‐enabled communication from single‐link transmission to spatially parallel operation, using a 16×16 OPA to generate eight parallel free‐space channels with an aggregate data rate of 8 Gbit/s. More recently, Li et al. [329] demonstrated an integrated silicon OPA transceiver capable of full‐duplex line‐of‐sight communication and non‐line‐of‐sight transmission through an OPA‐based repeater, with data rates of up to 220 Gbit/s. Kan et al. [330] further showed that, for FSO phase‐front correction, two‐dimensional OPAs can be engineered to prioritize narrow beam divergence and enhanced fundamental‐lobe power rather than wide‐angle scanning. Taken together, these studies indicate that OPAs are evolving from beam‐steering elements into versatile communication engines for directional transmission, parallel links, turbulence mitigation, and reconfigurable optical wireless networking.

Despite this progress, one important near‐term bottleneck for OPA‐based free‐space communication is compatibility with wavelength‐division multiplexing (WDM). In conventional phase‐shifter‐based OPAs, beam steering is realized through wavelength‐independent phase gradients across the array. Because the resulting steering angle scales explicitly with optical wavelength, different WDM channels are inherently directed to slightly different angles. This wavelength sensitivity prevents multiple channels from sharing a common optical path and therefore limits the scalability of OPA‐based transmitters in multi‐λ systems. The problem is further exacerbated in long‐range links, where atmospheric turbulence introduces additional wavelength‐dependent modal distortions that amplify interchannel imbalance.

Recent progress in free‐space optical communication has highlighted the importance of addressing these wavelength‐dependent effects from a complementary receiver‐side perspective. For example, Brinkley et al. demonstrated that WDM transmission over kilometer‐scale free‐space links is fundamentally challenged by chromatic modal distortions induced by atmospheric turbulence [331]. By employing a machine learning‐assisted multi‐wavelength receiver that learns wavelength‐dependent wavefront distortions and adaptively projects each channel onto its optimal modal basis, their approach significantly improves WDM robustness under turbulence. Although this work primarily addresses receiver‐ and channel‐side impairments, it underscores a broader requirement for future OPA‐based transmitters: Beam steering must eventually be decoupled from optical wavelength if OPAs are to function as scalable WDM‐compatible communication front ends.

Taken together, these considerations indicate that truly WDM‐compatible OPA transmitters will likely require architectural innovations beyond conventional phase‐gradient steering, such as true‐time‐delay beamforming or wavelength‐independent structured‐light generation. Addressing this challenge will be essential for fully exploiting the potential of OPAs in high‐capacity, long‐range optical wireless communication systems.

### 7.2 Emerging Applications

Beyond sensing and communication, optical phased arrays (OPAs) are increasingly enabling a new class of emerging applications that exploit their unique combination of solid‐state operation, high‐speed programmability, and precise free‐space wavefront control. A broader trend emerging from recent studies is that integrated OPAs are no longer being viewed merely as compact beam‐deflection devices but increasingly as programmable optical‐field platforms. In this perspective, their value lies not only in steering light but also in shaping, synthesizing, and manipulating free‐space optical fields in ways that support advanced functions in structured‐light generation, complex‐environment imaging, atomic and quantum photonics, reconfigurable optical reception, and even photonic information processing [126, 265, 332, 333, 334, 335, 336, 337, 338, 339, 340, 341, 342, 343]. These developments indicate that the role of OPAs is expanding from beam steering toward active optical‐field generation and manipulation across diverse physical domains.

A first major direction is programmable free‐space field generation, where OPAs are used not only to redirect light but also to synthesize tailored beam profiles and visible optical fields. OPAs have the potential to fundamentally reshape optical 3D printing by offering a compact, fully solid‐state, and programmable route to optical fabrication. With submicrometer precision and ultrafast beam steering, OPAs enable volumetric exposure on a millimeter‐scale chip, bypassing the mechanical constraints of conventional layer‐by‐layer approaches [344]. This opens avenues for portable manufacturing platforms for rapid prototyping and field repair, as well as microscale and nanoscale fabrication of functional devices in industrial and biomedical domains [345, 346, 347, 348]. OPA‐enabled 3D printing may further evolve into intelligent and highly automated platforms through integration with CMOS electronics, adaptive feedback, and advanced photochemical materials. This application direction is further reinforced by recent progress in structured‐light generation and visible‐spectrum optical‐field control. Integrated silicon and InP OPAs have been used to generate quasi‐Bessel, high‐order quasi‐Bessel, Hermite–Gaussian, and orbital‐angular‐momentum beams, while also enabling arbitrary far‐field pattern synthesis, directional nulling, and multibeam control [126, 265, 335, 336]. In addition, recent RGB integrated OPA demonstrations with simultaneous multiwavelength beam control and image projection suggest that visible‐spectrum OPAs may evolve into compact engines for display, optogenetics, chip‐scale 3D printing, and general visible‐light field synthesis [343]. Taken together, these studies show that emerging OPA applications increasingly rely on programmable beam formation rather than beam steering alone.

A second major direction is adaptive optical interaction with complex environments, where OPAs act as dynamically reconfigurable optical front ends for biological manipulation, imaging, and optical‐field delivery under nonideal propagation conditions. In biophotonic applications, OPAs are poised to transform optical tweezers by providing chip‐scale, fully reconfigurable, and high‐density beam control. Unlike conventional bulk or early integrated tweezers, OPAs enable nonmechanical manipulation over millimeter‐scale working distances, significantly expanding accessible biological environments—from standard culture wells to in vivo systems [34, 349]. High‐speed phase control supports precise trapping, translation, and multidimensional manipulation, whereas dense OPA integration opens the door to massively parallel tweezing for high‐throughput assays. These capabilities point toward compact platforms for single‐cell manipulation, molecular force spectroscopy, volumetric trapping, and high‐throughput biophotonics. A closely related emerging direction is imaging and wavefront control in complex optical environments. OPAs have been used for high‐speed wavefront shaping through scattering media, multimode‐fiber speckle imaging, and computational ghost imaging, thereby extending their role from beam delivery to active illumination control and computational imaging [333, 334, 341]. These developments are significant because they indicate that OPA‐based photonic chips may function not only as transmitters but also as compact optical front ends for imaging in scattering, multimode, or otherwise difficult environments.

A third emerging direction is the use of OPAs as compact optical front ends for display, reception, atomic interaction, and other nontraditional photonic functions. OPAs hold strong promise for next‐generation AR/VR and holographic display systems by enabling precise, high‐speed wavefront control at the chip scale [350, 351]. Unlike conventional head‐mounted displays that rely on stereoscopic cues and suffer from vergence–accommodation conflicts, OPAs provide dynamic and multidimensional beam shaping that supports high‐fidelity holographic reconstruction with natural depth perception. Their fast modulation, wide phase‐tuning range, and compact low‐power integration offer substantial advantages over conventional spatial light modulators, enabling rapid refresh, high pixel density, and extended field of view while mitigating diffraction‐induced contrast loss [352]. Beyond imaging and display, OPAs are also beginning to enter atomic and quantum photonics, as well as more general transceiver architectures. A representative example is a chip‐scale saturated absorption spectrometer based on a ${\text{Si}}_{3}N_{4}$ OPA integrated with a rubidium vapor cell, demonstrating that OPAs can support compact atomic references and free‐space atom–light interaction on chip [332]. At the same time, dual‐mode OPA receivers have shown that mode diversity and coherent combination can enlarge the reception field of view for LiDAR and free‐space optical systems [339, 340], whereas OPA‐enabled underwater optical links suggest that electronically steerable integrated beams may also benefit agile communication in propagation environments beyond air [342]. These studies indicate that future OPAs may increasingly evolve from transmitter‐side steering units into more general optical front ends for emission, reception, and environment‐adaptive beam control.

At a more exploratory level, several recent works suggest that OPA concepts can be extended into nontraditional information‐processing functions. Integrated OPAs have been demonstrated as XY Hamiltonian solvers for optical optimization [337], whereas phased‐array phase engineering has also been transferred to nonreciprocal multiport optical routing [338]. Although still at an early stage, these efforts indicate that the long‐term significance of OPAs may extend beyond sensing, communication, and imaging into broader roles in programmable photonic information processing.

Overall, recent progress indicates that integrated OPAs are transitioning from dedicated beam‐steering hardware into multifunctional programmable optical‐field platforms. Their emerging applications now span structured‐light generation, optical fabrication, complex‐media imaging, biophotonics, holographic display, atomic and quantum photonics, reconfigurable optical reception, underwater communication, and physics‐inspired photonic computing [126, 265, 332, 333, 334, 335, 336, 337, 338, 339, 340, 341, 342, 343].

### 7.3 Future Outlook

Looking forward, no single material platform is likely to simultaneously satisfy all the key requirements of high‐performance optical phased arrays (OPAs), including efficient light generation, low‐loss optical transport, high‐speed phase modulation, and high‐power operation. A central future direction is, therefore, heterogeneous integration, which combines the strengths of disparate materials on a unified substrate through techniques such as wafer bonding, transfer printing, and microassembly. In this framework, InP can provide efficient on‐chip light sources, ${\text{Si}}_{3}N_{4}$ can support ultralow‐loss distribution networks, and thin‐film lithium niobate (TFLN) can deliver high‐speed and low‐power phase modulation. Such heterogeneous integration is not only a particularly promising route toward high‐performance, multifunctional, and cost‐effective OPAs but is also likely to become a major driving force for the broader commercialization of large‐scale photonic integrated circuits.

Beyond materials and integration, the functional scope of OPAs is also expanding significantly. Recent advances in on‐chip structured‐light generation suggest that OPAs may evolve far beyond conventional beam steering into programmable engines for free‐space optical‐field synthesis. In particular, Bütow et al. demonstrated that a fully programmable integrated photonic processor can synthesize a broad set of free‐space field distributions—including Hermite–Gaussian, Laguerre–Gaussian, and superposed modes—with high fidelity using only a compact 4×4 emitter array [353]. Although developed in the context of programmable photonic meshes, such architectures can be interpreted as a generalized form of an optical phased array, in which both the amplitude and phase of each emitter are independently and dynamically controlled. This work points to an important conceptual shift: OPAs may evolve from beam‐steering components into reconfigurable optical‐field generators.

A complementary step toward practical deployment has been demonstrated at the system level, where structured‐light functionality is realized with scalability and compactness in mind. For example, Hu et al. demonstrated a row‐gated optical phased‐array architecture capable of generating dense programmable structured‐light patterns for three‐dimensional imaging [228]. By employing diode‐based row–column addressing, the system substantially reduced electrical control complexity while enabling single‐wavelength, eye‐safe structured‐light projection at 1550 nm. In this approach, horizontal subarray interference was exploited to generate high‐density point patterns, whereas vertical row gating enabled efficient two‐dimensional scanning without wavelength tuning. This work illustrates how OPA‐based structured‐light generation may progress from a proof of programmable field synthesis into a scalable, calibration‐efficient, and application‐oriented platform.

The ability to generate structured optical fields is particularly relevant for emerging communication and sensing systems, where mode‐division multiplexing, spatial diversity, and tailored wavefronts can provide additional degrees of freedom for increasing capacity and mitigating channel impairments. Structured modes such as orbital‐angular‐momentum and higher‐order spatial modes exhibit distinct propagation and scattering characteristics that, when combined with suitable detection and compensation strategies, may enable high‐dimensional encoding and improved robustness against turbulence. At the same time, the calibration and control of large‐scale programmable OPA‐like architectures naturally lend themselves to data‐driven and machine learning‐assisted optimization. Such methods may support adaptive field synthesis, real‐time distortion compensation, and intelligent beam control in systems with large parameter spaces and dynamic operating conditions.

Beyond device‐level scaling, an increasingly important future direction is the integration of OPAs into higher‐level system architectures. In this context, two trends are particularly noteworthy: the convergence of OPA beamforming with integrated sensing‐and‐communication frameworks and the expansion of OPA systems from single‐chip apertures toward modular multiaperture platforms.

One representative direction is optical wireless integrated sensing and communication (OW‐ISAC), in which OPAs may serve not merely as beam‐steering devices but as programmable spatial interfaces for jointly optimized communication‐and‐sensing networks. Wen et al. [354] explored this possibility by investigating the joint design of OPA beamforming and OFDM waveform optimization in an OW‐ISAC framework. By extending RF‐ISAC concepts into the optical domain, their work showed that OPA beamforming can be co‐optimized with communication power allocation and sensing‐subcarrier design under direct‐detection constraints. This result suggests that future OPAs may evolve from isolated beam‐control modules into key enabling infrastructure for integrated optical communication‐and‐sensing systems.

Another important direction concerns the expansion of OPA systems beyond the intrinsic field‐of‐view limitation of a single chip. Guo et al. [355] proposed a modular multiaperture OPA architecture for seamless full‐space beam steering, in which multiple wide‐field chip‐level OPAs were spatially tiled and coordinated to achieve continuous 4π‐steradian coverage. This approach points to a fundamentally different scaling route: Instead of relying exclusively on ever‐larger or denser single‐chip arrays, future full‐space optical perception may be realized through modular multichip coordination and system‐level aperture synthesis. Such architectures are particularly attractive for omnidirectional solid‐state LiDAR and full‐space sensing platforms.

Taken together, these developments indicate that OPAs are evolving from application‐specific beam‐steering components into general‐purpose, programmable optical front ends. In the longer term, their competitiveness will depend increasingly on system‐level functional convergence, architectural scalability, and the ability to operate as programmable optical infrastructure within larger photonic platforms. Through continued advances in heterogeneous integration, structured‐light generation, intelligent control, and modular system design, OPAs are poised to become a foundational technology for future free‐space communication, intelligent sensing, advanced manufacturing, and immersive display systems.

## 8 Conclusion

On‐chip optical phased arrays (OPAs) have emerged as a cornerstone of photonic integration, enabling compact, digitally reconfigurable beam steering that overcomes the inertia, scalability, and reliability limitations of mechanical systems. Their rapid progress has been driven by advances in key building blocks—including on‐chip lasers, phase shifters, and grating antennas—across mature silicon, silicon nitride, and III–V material platforms, whereas thin‐film lithium niobate on insulator (LNOI) has recently redefined performance boundaries through its unique combination of ultralow loss, strong electro‐optic response, and high‐speed operation. Together, these complementary platforms illustrate the increasingly heterogeneous and system‐oriented nature of OPA development.

Despite these advances, significant challenges remain before large‐scale OPAs can be widely deployed. Reducing electronic overhead, suppressing optical and thermal crosstalk, mitigating grating lobes, and achieving robust calibration across large apertures remain critical bottlenecks. Addressing these challenges will require holistic design strategies that extend beyond individual devices, encompassing material innovation, photonic–electronic cointegration, advanced packaging, and system‐level co‐optimization. In particular, emerging approaches such as heterogeneous integration, aperiodic and structured‐light array architectures, and co‐packaged electronic–photonic systems are expected to play central roles in overcoming current scalability limits.

Looking forward, OPAs are poised to transition from laboratory demonstrations into versatile, programmable optical front ends underpinning a broad range of technologies, including solid‐state LiDAR, free‐space optical communication, imaging, structured‐light generation, and holographic displays. As integration density, control intelligence, and fabrication maturity continue to improve, OPAs may ultimately evolve from application‐specific beam‐steering components into general‐purpose platforms for high‐dimensional wavefront synthesis and optical information processing, shaping the future landscape of integrated photonics.

## Author Contributions

**Jingwei Li:** writing – review and editing, writing – original draft, conceptualization, methodology, software, data curation, investigation. **Yuyuan Han:** writing – review and editing, writing – original draft. **Aoxiang Chen:** writing – review and editing, writing – original draft. **Pengpeng Li:** writing – review and editing, writing – original draft. **Xinglin Zhu:** writing – review and editing, writing – original draft. **Guanbao Zhao:** writing – review and editing, writing – original draft. **Luohan Peng:** funding acquisition, writing – original draft, writing – review and editing. **Huaibin Zheng:** writing – review and editing, writing – original draft.

## Funding

The authors have nothing to report.

## Conflicts of Interest

The authors declare no conflicts of interest.

## Data Availability

The data that support the findings of this study are available from the corresponding author upon reasonable request.

## References

[1] D. Thomson , A. Zilkie , J. E. Bowers , et al., “Roadmap on Silicon Photonics,” Journal of Optics 18, no. 7 (2016): 073003, 10.1088/2040-8978/18/7/073003.

[2] H. Lee , C. Tong , Li Jiang , O. Painter , and K. J. Vahala , “Ultra‐Low‐Loss Optical Delay Line on a Silicon Chip,” Nature Communications 3, no. 1 (2012): 867, 10.1038/ncomms1876. 22643894

[3] Z. Ye , P. Zhao , K. Twayana , M. Karlsson , V. Torres‐Company , and P. A. Andrekson , “Overcoming the Quantum Limit of Optical Amplification in Monolithic Waveguides,” Science Advances 7, no. 38 (2021): eabi8150, 10.1126/sciadv.abi8150. PMC8443169 34524857

[4] J. Liu , G. Huang , R. N. Wang , et al., “High‐Yield, Wafer‐Scale Fabrication of Ultralow‐Loss, Dispersion‐Engineered Silicon Nitride Photonic Circuits,” Nature Communications 12, no. 1 (2021): 2236, 10.1038/s41467-021-21973-z. PMC8052462 33863901

[5] J. F. Bauters , M. J. R. Heck , D. John , et al., “Ultra‐Low‐Loss High‐Aspect‐Ratio Si3N4 Waveguides,” Optics Express 19, no. 4 (2011): 3163–3174, 10.1364/oe.19.003163. 21369138

[6] Y. Gao , Yi Sun , I. Rebolledo‐Salgado , R. Van Laer , V. Torres‐Company , and J. Schröder , “Tightly‐Confined and Long Z‐Cut Lithium Niobate Waveguide With Ultralow‐Loss,” Laser & Photonics Reviews 19, no. 21 (2025): e00042, 10.1002/lpor.202500042.

[7] G. Zhao , J. Zhang , M. Zhu , L. Peng , and J. Xiao , “Demonstration of a Thin‐Film Lithium Niobate Modulator With Integrated Bias Controllers,” IEEE Photonics Technology Letters 37, no. 20 (2025): 1201–1204, 10.1109/lpt.2025.3585706.

[8] J. Wang , S. Xu , J. Chen , and W. Zou , “A Heterogeneous Silicon on Lithium Niobate Modulator for Ultra‐Compact and High‐Performance Photonic Integrated Circuits,” IEEE Photonics Journal 13, no. 1 (2021): 1–12, 10.1109/jphot.2020.3048690.

[9] W. Zhang , S. Yang , S. Zheng , and B. Wang , “Low‐Loss Ultra‐Compact Silicon Photonic Integrated Micro‐Disk Modulator for Large‐Scale WDM Optical Interconnection,” Journal of Lightwave Technology 42, no. 9 (2024): 3306–3313, 10.1109/jlt.2024.3361068.

[10] C. Wang , M. Zhang , B. Stern , M. Lipson , and M. Loncar , “Nanophotonic Lithium Niobate Electro‐Optic Modulators,” Optics Express 26, no. 2 (2018): 1547–1555, 10.1364/oe.26.001547. 29402028

[11] B. Shen , P. Wang , R. Polson , and R. Menon , “An Integrated‐Nanophotonics Polarization Beamsplitter With 2.4 x 2.4μm^2^ Footprint,” Nature Photonics 9, no. 6 (2015): 378–382, 10.1038/nphoton.2015.80.

[12] C. Li and D. Dai , “Compact Polarization Beam Splitter for Silicon Photonic Integrated Circuits With a 340‐nm‐Thick Silicon Core Layer,” Optics Letters 42, no. 21 (2017): 4243–4246, 10.1364/ol.42.004243. 29088134

[13] H.‐S. Jang , D. Lee , H. Heo , et al., “Aluminum Nitride Waveguide Beam Splitters for Integrated Quantum Photonic Circuits,” Photonics Research 11, no. 7 (2023): 1196–1202, 10.1364/prj.461723.

[14] M. Garrett , Y. Liu , M. Merklein , et al., “Integrated Microwave Photonic Notch Filter Using a Heterogeneously Integrated Brillouin and Active‐Silicon Photonic Circuit,” Nature Communications 14, no. 1 (2023): 7544, 10.1038/s41467-023-43404-x. PMC10662262 37985657

[15] M. Jalali Mehrabad , A. P. Foster , N. J. Martin , et al., “Chiral Topological Add‐Drop Filter for Integrated Quantum Photonic Circuits,” Optica 10, no. 3 (2023): 415–421, 10.1364/optica.481684.

[16] G. Liu , K. Ye , O. Daulay , Q. Tan , H. Yu , and D. Marpaung , “Linearized Integrated Microwave Photonic Circuit for Filtering and Phase Shifting,” APL Photonics 8, no. 5 (2023): 051303, 10.1063/5.0148464.

[17] M. J. R. Heck , “Highly Integrated Optical Phased Arrays: Photonic Integrated Circuits for Optical Beam Shaping and Beam Steering,” Nanophotonics 6, no. 1 (2017): 93–107, 10.1515/nanoph-2015-0152.

[18] K. Wang , A. Nirmalathas , C. Lim , et al., “High‐speed indoor optical wireless communication system employing a silicon integrated photonic circuit,” Optics Letters 43, no. 13 (2018): 3132–3135, 10.1364/ol.43.003132. 29957797

[19] C. Haffner , W. Heni , Y. Fedoryshyn , et al., “All‐Plasmonic Mach‐Zehnder Modulator Enabling Optical High‐Speed Communication at the Microscale,” Nature Photonics 9, no. 8 (2015): 525+–528, 10.1038/nphoton.2015.127.

[20] B. J. Shastri , A. N. Tait , T. Ferreira de Lima , et al., “Photonics for Artificial Intelligence and Neuromorphic Computing,” Nature Photonics 15, no. 2 (2021): 102–114, 10.1038/s41566-020-00754-y.

[21] W. Bogaerts , D. Perez , J. Capmany , et al., “Programmable Photonic Circuits,” Nature 586, no. 7828 (2020): 207–216, 10.1038/s41586-020-2764-0. 33028997

[22] T. Jin , J. Zhou , and P. T. Lin , “Real‐Time and Non‐Destructive Hydrocarbon Gas Sensing Using Mid‐Infrared Integrated Photonic Circuits,” RSC Advances 10, no. 13 (2020): 7452–7459, 10.1039/c9ra10058j. PMC7793566 33425327

[23] A. Perez‐Serrano , C. Quevedo‐Galan , V. R. Aguilera , P. Castera , J. M. G. Tijero , and I. Esquivias , “Differential Absorption LiDAR Transmitter Based on an Indium Phosphide Photonic Integrated Circuit for Carbon Dioxide Sensing,” IEEE Journal of Selected Topics in Quantum Electronics 28, no. 5 (2022), 10.1109/JSTQE.2022.3156183.

[24] A. W. Elshaari , W. Pernice , K. Srinivasan , O. Benson , and V. Zwiller , “Hybrid Integrated Quantum Photonic Circuits,” Nature Photonics 14, no. 5 (2020): 285–298, 10.1038/s41566-020-0609-x. PMC8607459 34815738

[25] Y. Ding , D. Bacco , K. Dalgaard , et al., “High‐Dimensional Quantum Key Distribution Based on Multicore Fiber Using Silicon Photonic Integrated Circuits,” NPJ Quantum Information 3, no. 1 (2017): 25, 10.1038/s41534-017-0026-2.

[26] C. V. Poulton , A. Yaacobi , D. B. Cole , et al., “Coherent Solid‐State LiDAR With Silicon Photonic Optical Phased Arrays,” Optics Letters 42, no. 20 (2017): 4091–4094, 10.1364/ol.42.004091. 29028020

[27] C. Vincent Poulton , M. J. Byrd , P. Russo , et al., “Long‐Range LiDAR and Free‐Space Data Communication With High‐Performance Optical Phased Arrays,” IEEE Journal of Selected Topics in Quantum Electronics 25, no. 5 (2019): 1–8, 10.1109/jstqe.2019.2908555.

[28] C.‐P. Hsu , B. Li , B. Solano‐Rivas , et al., “A Review and Perspective on Optical Phased Array for Automotive LiDAR,” IEEE Journal of Selected Topics in Quantum Electronics 27, no. 1 (2020): 1–16, 10.1109/jstqe.2020.3022948.

[29] C. V. Poulton , M. J. Byrd , P. Russo , et al., “Coherent LiDAR With an 8,192‐Element Optical Phased Array and Driving Laser,” IEEE Journal of Selected Topics in Quantum Electronics 28, no. 5 (2022): 1–8, 10.1109/jstqe.2022.3187707.

[30] N. Dostart , B. Zhang , A. Khilo , et al., “Serpentine Optical Phased Arrays for Scalable Integrated Photonic LiDAR Beam Steering,” Optica 7, no. 6 (2020): 726–733, 10.1364/optica.389006.

[31] H. Zheng , Y. Han , L. Qiu , et al., “Long‐Range Imaging LiDAR With Multiple Denoising Technologies,” Applied Sciences 14, no. 8 (2024): 3414, 10.3390/app14083414.

[32] Y. Wu , S. Shao , Y. Li , et al., “Multi‐Beam Optical Phase Array for Long‐Range LiDAR and Free‐Space Data Communication,” Optics and Laser Technology 151 (2022): 108027, 10.1016/j.optlastec.2022.108027.

[33] J. Notaros , “Integrated Optical Phased Arrays for Augmented Reality, LiDAR, and Beyond,” in 2022 Optical Fiber Communications Conference And Exhibition (OFC) (IEEE, 2022): AC Photon Inc; Alibaba Grp; Amphenol; Anritsu; Ciena; Corning; Effect Photon; Infinera; Ligentec; OFS; OZOptics; Poet Technologies; Samtec; Sanwa; Usconec; VeEX; Xilinx; VPIphotonics. Optical Fiber Communications Conference and Exhibition (OFC), San Diego, CA, MAR 06‐10, 2022.

[34] T. Sneh , S. Corsetti , M. Notaros , K. Kikkeri , J. Voldman , and J. Notaros , “Optical Tweezing of Microparticles and Cells Using Silicon‐Photonics‐Based Optical Phased Arrays,” Nature Communications 15, no. 1 (2024): 8493, 10.1038/s41467-024-52273-x. PMC11450221 39362852

[35] Li Jie , Y. Xue , Z. Yan , Yu Han , and K. May Lau , “III‐V Selective Regrowth on SOI for Telecom Lasers in Silicon Photonics,” Journal of Applied Physics 133, no. 13 (2023), 10.1063/5.0144377.

[36] J. Li , Y. Xue , L. Lin , Z. Xing , K. S. Wong , and K. M. Lau , “Telecom InGaAs/InP Quantum Well Lasers Laterally Grown on Silicon‐on‐Insulator,” Journal of Lightwave Technology 40, no. 16 (2022): 5631–5635, 10.1109/jlt.2022.3181723.

[37] Z. Wang , B. Tian , M. Pantouvaki , et al., “Room‐Temperature InP Distributed Feedback Laser Array Directly Grown on Silicon,” Nature Photonics 9, no. 12 (2015): 837–842, 10.1038/nphoton.2015.199.

[38] D. Gonzalez‐Andrade , S. Guerber , E. Duran‐Valdeiglesias , et al., “Ultra‐Wideband Dual‐Polarization Silicon Nitride Power Splitter Based on Modal Engineered Slot Waveguides,” Optics Letters 45, no. 2 (2020): 527–530, 10.1364/ol.383757.

[39] L. Chrostowski and M. Hochberg , Silicon Photonics Design: From Devices to Systems (Cambridge University Press, 2015).

[40] Xi Xiao , M. Li , L. Wang , D. Chen , Qi Yang , and S. Yu , “High speed silicon photonic modulators,” in Optical Fiber Communication Conference, Tu2H–1 (Optica Publishing Group, 2017).

[41] L. Qiao , W. Tang , and T. Chu , “32 × 32 Silicon Electro‐Optic Switch With Built‐in Monitors and Balanced‐Status Units,” Scientific Reports 7, no. 1 (2017): 42306, 10.1038/srep42306. PMC5299409 28181557

[42] Z. Wang , X. Li , J. Ji , et al., “Fast‐Speed and Low‐Power‐Consumption Optical Phased Array Based on Lithium Niobate Waveguides,” Nanophotonics 13, no. 13 (2024): 2429–2436, 10.1515/nanoph-2024-0066. PMC11501545 39633667

[43] W.‐B. Lee , Y.‐J. Kwon , Do‐H. Kim , Y.‐Ho Sunwoo , and S.‐S. Lee , “Hybrid Integrated Thin‐Film Lithium Niobate–Silicon Nitride Electro‐Optical Phased Array Incorporating Silicon Nitride Grating Antenna for Two‐Dimensional Beam Steering,” Optics Express 32, no. 6 (2024): 9171–9183, 10.1364/oe.518961. 38571156

[44] W. Li , Xu Zhao , J. He , et al., “High‐Speed 2D Beam Steering based on a Thin‐Film Lithium Niobate Optical Phased Array With a Large Field of View,” Photonics Research 11, no. 11 (2023): 1912–1918, 10.1364/prj.502439.

[45] G. Yue and Y. Li , “Integrated Lithium Niobate Optical Phased Array for Two‐Dimensional Beam Steering,” Optics Letters 48, no. 14 (2023): 3633–3636, 10.1364/ol.491748. 37450712

[46] J. Li , Y. He , H. Zheng , et al., “Cascaded Domain Engineering Optical Phased Array for Beam Steering,” Applied Physics Reviews 10, no. 3 (2023): 031413, 10.1063/5.0135099. PMC11501413 39635637

[47] J. Chen , W. Li , Z. Kang , et al., “Single Soliton Microcomb Combined With Optical Phased Array for Parallel FMCW LiDAR,” Nature Communications 16, no. 1 (2025): 1056, 10.1038/s41467-025-56483-9. PMC11770108 39865065

[48] Y. Wu , R. Jiang , Y. Wang , and W. Fu , “Hybrid Integrated Silicon Nitride Optical Phased Array With Phase Calibration for Two‐Dimensional Beam Steering,” Optics Express 33, no. 5 (2025): 11123–11137, 10.1364/oe.539929. 40798741

[49] J. Wu , Z. Liang , X. Wang , et al., “End‐Fire Optical Phased Array for Passive Beam Steering on Thin‐Film Lithium Niobate,” Optics Letters 49, no. 18 (2024): 5087–5090, 10.1364/ol.536761. 39270236

[50] M. R. Kossey , C. Rizk , and A. C. Foster , “End‐Fire Silicon Optical Phased Array With Half‐Wavelength Spacing,” APL Photonics 3, no. 1 (2018): 011301, 10.1063/1.5000741.

[51] S. Y. Siew , B. Li , F. Gao , et al., “Review of Silicon Photonics Technology and Platform Development,” Journal of Lightwave Technology 39, no. 13 (2021): 4374–4389, 10.1109/jlt.2021.3066203.

[52] A. Rahim , E. Ryckeboer , A. Z. Subramanian , et al., “Expanding the Silicon Photonics Portfolio With Silicon Nitride Photonic Integrated Circuits,” Journal of Lightwave Technology 35, no. 4 (2017): 639–649: Optical Fiber Communications Conference and Exhibition (OFC), Anaheim, CA, MAR 20‐24, 2016, 10.1109/jlt.2016.2617624.

[53] L. M. Augustin , R. Santos , E. den Haan , et al., “InP‐Based Generic Foundry Platform for Photonic Integrated Circuits,” IEEE Journal of Selected Topics in Quantum Electronics 24, no. 1 (2018): 1–10, 10.1109/jstqe.2017.2720967.

[54] V. Rustichelli , C. Calo , F. Lemaitre , et al., “Monolithic Integration of Buried‐Heterostructures in a Generic Integrated Photonic Foundry Process,” IEEE Journal of Selected Topics in Quantum Electronics 25, no. 5 (2019): 1–8, 10.1109/jstqe.2019.2927576.

[55] F. Lemaitre , C. Fortin , N. Lagay , et al., “Foundry Photonic Process Extension With Bandgap Tuning Using Selective Area Growth,” IEEE Journal of Selected Topics in Quantum Electronics 25, no. 5 (2019): 1–8, 10.1109/jstqe.2019.2922069.

[56] J. J. G. M. van der Tol , Y. S. Oei , U. Khalique , R. Notzel , and M. K. Smit , “InP‐Based Photonic Circuits: Comparison of Monolithic Integration Techniques,” Progress in Quantum Electronics 34, no. 4 (2010): 135–172, 10.1016/j.pquantelec.2010.02.001.

[57] Yi Wang , Y. Jiao , and K. Williams , “Scaling Photonic Integrated Circuits With InP technology: A Perspective,” APL Photonics 9, no. 5 (2024): 050902, 10.1063/5.0200861.

[58] J. Lin , F. Bo , Ya Cheng , and J. Xu , “Advances in On‐Chip Photonic Devices Based on Lithium Niobate on Insulator,” Photonics Research 8, no. 12 (2020): 1910–1936, 10.1364/prj.395305.

[59] L. Huang , C. Yang , L. Liang , et al., “Integrated Light Sources Based on Micro‐Ring Resonators for Chip‐Based LiDAR,” Laser & Photonics Reviews 19, no. 2 (2025): 2400343, 10.1002/lpor.202400343.

[60] Di Zhu , L. Shao , M. Yu , et al., “Integrated Photonics on Thin‐Film Lithium Niobate,” Advances In Optics and Photonics 13, no. 2 (2021): 242–352, 10.1364/aop.411024.

[61] Z. Xie , Bo Fang , J. Lin , et al., “Recent Development in Integrated Lithium Niobate Photonics,” Advances In Physics‐X 9, no. 1 (2024), 10.1080/23746149.2024.2322739.

[62] B. Bhandari , C. Wang , Ji‐Y. Gwon , et al., “Dispersive Silicon–Nitride Optical Phased Array Incorporating Arrayed Waveguide Delay Lines for Passive Line Beam Scanning,” Scientific Reports 12, no. 1 (2022): 18759, 10.1038/s41598-022-23456-7. PMC9637176 36335252

[63] K. Van Acoleyen , W. Bogaerts , and R. Baets , “Two‐Dimensional Dispersive Off‐Chip Beam Scanner Fabricated on Silicon‐on‐Insulator,” IEEE Photonics Technology Letters 23, no. 17 (2011): 1270–1272, 10.1109/lpt.2011.2159785.

[64] M. Kandil , M. S. Dahlem , and W. Bogaerts , “System Trade‐Offs in a Swept Source FMCW LiDAR With Dispersive OPA Beam steering,” in Smart Photonic and Optoelectronic Integrated Circuits 2024, vol. 12890 (2024), 102–114.

[65] W. Bogaerts , S. Dwivedi , R. Jansen , X. Rottenberg , and M. S. Dahlem , “A 2D Pixelated Optical Beam Scanner Controlled by the Laser Wavelength,” IEEE Journal of Selected Topics in Quantum Electronics 27, no. 1 (2020): 1–12, 10.1109/jstqe.2020.3017230.

[66] C. Wang , W.‐B. Lee , Y.‐Ho Sunwoo , Y.‐J. Kwon , M.‐C. Oh , and S.‐S. Lee , “Angularly Offset Multiline Dispersive Optical Phased Array Enabling Large Field of View and Plateau Envelope,” Optics Express 32, no. 8 (2024): 13048–13064, 10.1364/oe.515498. 38859285

[67] M. Kandil , M. Prost , J. Kjellman , W. Bogaerts , and M. Dahlem , “Experimental Evaluation of Continuous and Pixelated Dispersive Optical Phased Arrays for 2D Beam Steering,” Photonics Research 13, no. 5 (2025): 1330–1340, 10.1364/prj.546057.

[68] W. G. Joseph , Introduction to Fourier Optics (Roberts and Company Publishers, 2005).

[69] K. Van Acoleyen , W. Bogaerts , J. Jágerská , N. Le Thomas , R. Houdré , and R. Baets , “Off‐Chip Beam Steering With a One‐Dimensional Optical Phased Array on Silicon‐on‐Insulator,” Optics Letters 34, no. 9 (2009): 1477–1479, 10.1364/ol.34.001477. 19412311

[70] Y. Li , B. Chen , Q. Na , et al., “Wide‐Steering‐Angle High‐Resolution Optical Phased Array,” Photonics Research 9, no. 12 (2021): 2511–2518, 10.1364/prj.437846.

[71] C. Sun , B. Li , Wu Shi , et al., “Large‐Scale and Broadband Silicon Nitride Optical Phased Arrays,” IEEE Journal of Selected Topics in Quantum Electronics 28, no. 6 (2022): 1–10: High Density Integr. Multipurpose Photon. Circ, 10.1109/jstqe.2022.3182306.

[72] L. Yu , P. Ma , G. Luo , et al., “Adoption of Large Aperture Chirped Grating Antennas in Optical Phase Array for Long Distance Ranging,” Optics Express 30, no. 15 (2022): 28112–28120, 10.1364/oe.464358. 36236966

[73] H. Qiu , Y. Liu , X. Meng , X. Guan , Y. Ding , and H. Hu , “Bidirectional High Sidelobe Suppression Silicon Optical Phased Array,” Photonics Research 11, no. 4 (2023): 659–668, 10.1364/prj.479880.

[74] Z. Wang , L. Yu , Y. Yang , et al., “Wide Field of View Optical Phased Array With a High‐Directionality Antenna,” Optics Express 31, no. 13 (2023): 21192–21199, 10.1364/oe.492317. 37381224

[75] M. Ye , Y. Chen , J. Li , et al., “Large Steering Range and Low‐Loss Integrated Optical Phased Array With SiN‐Si Dual‐Layer Non‐Uniform Antenna,” Optics Express 31, no. 26 (2023): 44564–44574, 10.1364/oe.507381. 38178524

[76] W. Xu , Y. Guo , X. Li , et al., “Fully Integrated Solid‐State LiDAR Transmitter on a Multi‐Layer Silicon‐Nitride‐on‐Silicon Photonic Platform,” Journal of Lightwave Technology 41, no. 3 (2022): 832–840, 10.1109/JLT.2022.3204096.

[77] W. Xu , Q. Yuan , Y. Yang , L. Lu , J. Chen , and L. Zhou , “Progress and Prospects for LiDAR‐Oriented Optical Phased Arrays Based on Photonic Integrated Circuits,” npj Nanophotonics 2, no. 1 (2025): 14, 10.1038/s44310-025-00059-4.

[78] M. I. Skolnik , Introduction to Radar Systems, Vol. 3 (McGraw‐Hill, 1980).

[79] K. Wang , Y. Wang , S. Gao , et al., “2 × 2 Silicon Integrated Optical Phased Array for Beam Steering Applications,” in 2015 International Topical Meeting on Microwave Photonics (MWP) (2015), 1–4.

[80] T. Fukui , R. Tanomura , K. Komatsu , et al., “Non‐Redundant Optical Phased Array,” Optica 8, no. 10 (2021): 1350–1358, 10.1364/optica.437453.

[81] H. Qiu , Y. Liu , X. Meng , X. Guan , Y. Ding , and H. Hu , “Ultra‐Sparse Aperiodic Silicon Optical Phased Array Using High‐Performance Thermo‐Optic Phase Shifter,” Laser & Photonics Reviews 18, no. 10 (2024): 2301177, 10.1002/lpor.202301177.

[82] Y. Zhang , H. Chang , Z. Nie , et al., “High‐Speed Reconfigurable Spatiotemporal Beam Scanning Enabled by a Frequency‐Encoded Optical Phased Array,” Optics Letters 50, no. 4 (2025): 1212–1215, 10.1364/ol.544872. 39951766

[83] Y. Liu and H. Hu , “Silicon Optical Phased Array With a 180‐Degree Field of View for 2D optical Beam Steering,” Optica 9, no. 8 (2022): 903–907, 10.1364/optica.458642.

[84] W. Li , J. Chen , D. Liang , D. Dai , and Y. Shi , “Silicon Optical Phased Array With Calibration‐Free Phase Shifters,” Optics Express 30, no. 24 (2022): 44029–44038, 10.1364/oe.475350. 36523087

[85] F. Ashtiani and F. Aflatouni , “N × N Optical Phased Array With 2N Phase Shifters,” Optics Express 27, no. 19 (2019): 27183–27190, 10.1364/oe.27.027183. 31674584

[86] D. Fowler , S. Guerber , O. Castany , et al., “SiN Optical Phased Array Directly Coupled to a High Power 905 nm Tapered Diode Laser,” Optics Express 33, no. 19 (2025): 40180–40190, 10.1364/oe.569871. 41215374

[87] J. K. Doylend , M. J. R. Heck , J. T. Bovington , et al., “Hybrid III/V Silicon Photonic Source With Integrated 1D Free‐Space Beam Steering,” Optics Letters 37, no. 20 (2012): 4257–4259, 10.1364/ol.37.004257. 23073429

[88] W. Guo , P. R. A. Binetti , C. Althouse , et al., “Two‐Dimensional Optical Beam Steering With InP‐Based Photonic Integrated Circuits,” IEEE Journal of Selected Topics in Quantum Electronics 19, no. 4 (2013): 6100212, 10.1109/jstqe.2013.2238218.

[89] J. C. Hulme , J. K. Doylend , M. J. R. Heck , et al., “Fully Integrated Hybrid Silicon Two Dimensional Beam Scanner,” Optics Express 23, no. 5 (2015): 5861–5874, 10.1364/oe.23.005861. 25836813

[90] J. Notaros , N. Li , C. V. Poulton , et al., “CMOS‐Compatible Optical Phased Array Powered by a Monolithically‐Integrated Erbium Laser,” Journal of Lightwave Technology 37, no. 24 (2019): 5982–5987, 10.1109/jlt.2019.2944607.

[91] Y. Zhu , S. Zeng , and L. Zhu , “Optical Beam Steering by Using Tunable, Narrow‐Linewidth Butt‐Coupled Hybrid Lasers in a Silicon Nitride Photonics Platform,” Photonics Research 8, no. 3 (2020): 375–380, 10.1364/prj.382852.

[92] Y. Misugi , H. Okayama , and T. Kita , “Compact and Low Power‐Consumption Solid‐State Two‐Dimensional Beam Scanner Integrating a Passive Optical Phased Array and Hybrid Wavelength‐Tunable Laser Diode,” Journal of Lightwave Technology 41, no. 11 (2023): 3505–3512, 10.1109/jlt.2023.3244847.

[93] B. J. Frey , D. B. Leviton , and T. J. Madison , “Temperature‐Dependent Refractive Index of Silicon and Germanium,” in Optomechanical Technologies for Astronomy, vol. 6273 (SPIE, 2006), 790–799. 10.1117/12.672850.

[94] K. Van Acoleyen , K. Komorowska , W. Bogaerts , and R. Baets , “One‐Dimensional Off‐Chip Beam Steering and Shaping Using Optical Phased Arrays on Silicon‐on‐Insulator,” Journal of Lightwave Technology 29, no. 23 (2011): 3500–3505, 10.1109/jlt.2011.2171477.

[95] M. Prost , Y.‐C. Ling , S. Cakmakyapan , et al., “Solid‐State MWIR Beam Steering Using Optical Phased Array on Germanium‐Silicon Photonic Platform,” IEEE Photonics Journal 11, no. 6 (2019): 1–9, 10.1109/jphot.2019.2953222.

[96] W. Xu , L. Zhou , L. Lu , and J. Chen , “Aliasing‐Free Optical Phased Array Beam‐Steering With a Plateau Envelope,” Optics Express 27, no. 3 (2019): 3354–3368, 10.1364/oe.27.003354. 30732357

[97] Z. Du , C. Hu , G. Cao , et al., “Integrated Wavelength Beam Emitter on Silicon for Two‐Dimensional Optical Scanning,” IEEE Photonics Journal 11, no. 6 (2019): 1–10, 10.1109/jphot.2019.2943641.

[98] S. Zhu , T. Hu , Yu Li , et al., “CMOS‐Compatible Integrated Silicon Nitride Optical Phase Array for Electrically Tunable Off‐Chip Laser Beam Steering,” in 2019 Electron Devices Technology and Manufacturing Conference (EDTM) (IEEE, 2019), 228–230.

[99] C. V. Poulton , P. Russo , E. Timurdogan , et al., “High‐Performance Integrated Optical Phased Arrays for Chip‐Scale Beam Steering and Lidar,” in CLEO: Applications and Technology, ATu3R–2 (Optica Publishing Group, 2018).

[100] S.W. Chung , M. Nakai , and H. Hashemi , “Low‐Power Thermo‐Optic Silicon Modulator for Large‐Scale Photonic Integrated Systems,” Optics Express 27, no. 9 (2019): 13430–13459, 10.1364/oe.27.013430. 31052866

[101] R. L. Espinola , M. C. Tsai , J. T. Yardley , and R. M. Osgood , “Fast and Low‐Power Thermooptic Switch on Thin Silicon‐on‐Insulator,” IEEE Photonics Technology Letters 15, no. 10 (2003): 1366–1368, 10.1109/lpt.2003.818246.

[102] M. Chalupnik , A. Singh , J. Leatham , M. Lončar , and M. Soltani , “Scalable and Ultralow Power Silicon Photonic Two‐Dimensional Phased Array,” APL Photonics 8, no. 5 (2023): 051305, 10.1063/5.0139538.

[103] S. Yan , X. Zhu , L. Hagedorn Frandsen , et al., “Slow‐Light‐Enhanced Energy Efficiency for Graphene Microheaters on Silicon Photonic Crystal Waveguides,” Nature Communications 8, no. 1 (2017): 14411, 10.1038/ncomms14411. PMC5309776 28181531

[104] M. R. Watts , J. Sun , C. DeRose , D. C. Trotter , R. W. Young , and G. N. Nielson , “Adiabatic Thermo‐Optic Mach–Zehnder Switch,” Optics Letters 38, no. 5 (2013): 733–735, 10.1364/ol.38.000733. 23455281

[105] K. Murray , Z. Lu , H. Jayatilleka , and L. Chrostowski , “Dense Dissimilar Waveguide Routing for Highly Efficient Thermo‐Optic Switches on Silicon,” Optics Express 23, no. 15 (2015): 19575–19585, 10.1364/oe.23.019575. 26367615

[106] P. Sun and R. M. Reano , “Submilliwatt Thermo‐Optic Switches Using Free‐Standing Silicon‐on‐Insulator Strip Waveguides,” Optics Express 18, no. 8 (2010): 8406–8411, 10.1364/oe.18.008406. 20588686

[107] Z. Lu , K. Murray , H. Jayatilleka , and L. Chrostowski , “Michelson Interferometer Thermo‐Optics Witch on SOI With a 50‐*μ*W Power Consumption,” IEEE Photonics Technology Letters 27, no. 22 (2015): 2319–2322, 10.1109/LPT.2015.2462341.

[108] H. Qiu , Y. Liu , C. Luan , D. Kong , H. Hu , and Y. Ding , “Energy‐Efficient Thermo‐Optic Silicon Phase Shifter With Well‐Balanced Overall Performance,” Optics Letters 45, no. 17 (2020): 4806, 10.1364/ol.400230. 32870862

[109] H. Qiu , Y. Liu , X. Meng , X. Guan , Y. Ding , and H. Hu , “Energy‐Efficient Integrated Silicon Optical Phased Array,” Frontiers of Optoelectronics 16, no. 1 (2023): 23, 10.1007/s12200-023-00076-1. PMC10516849 37737551

[110] A. Densmore , S. Janz , R. Ma , et al., “Compact and Low Power Thermo‐Optic Switch Using Folded Silicon Waveguides,” Optics Express 17, no. 13 (2009): 10457–10465, 10.1364/oe.17.010457. 19550441

[111] Y. Wang , L. Liang , Y. Chen , et al., “Improved Performance of Optical Phased Arrays Assisted by Transparent Graphene Nanoheaters and Air Trenches,” RSC Advances 8, no. 15 (2018): 8442–8449, 10.1039/c7ra13154b. PMC9078513 35542001

[112] H. W. Yoo , N. Druml , D. Brunner , et al., “MEMS‐Based LiDAR for Autonomous Driving,” e & i Elektrotechnik und Informationstechnik 135, no. 6 (2018): 408–415, 10.1007/s00502-018-0635-2.

[113] G. Kang , S.‐H. Kim , J.‐B. You , et al., “Silicon‐Based Optical Phased Array Using Electro‐Optic $ p $‐$ i $‐$ n $ Phase Shifters,” IEEE Photonics Technology Letters 31, no. 21 (2019): 1685–1688, 10.1109/lpt.2019.2939550.

[114] F. Aflatouni , B. Abiri , A. Rekhi , and H. Ali , “Nanophotonic Projection System,” Optics Express 23, no. 16 (2015): 21012–21022, 10.1364/oe.23.021012. 26367953

[115] C. Zhao , C. Peng , and W. Hu , “Blueprint for Large‐Scale Silicon Optical Phased Array Using Electro‐Optical Micro‐Ring Pixels,” Scientific Reports 7, no. 1 (2017): 17727, 10.1038/s41598-017-18040-3. PMC5735160 29255245

[116] W. Xie , T. Komljenovic , J. Huang , M. L. Davenport , and J. E. Bowers , “Dense III‐V/Si Phase‐Shifter Based Optical Phased Array,” Preprint arXiv:1904.01104 (2019).

[117] S.‐H. Kim , J.‐B. You , Y.‐Gi Ha , et al., “Thermo‐Optic Control of the Longitudinal Radiation Angle in a Silicon‐Based Optical Phased Array,” Optics Letters 44, no. 2 (2019): 411–414, 10.1364/ol.44.000411. 30644913

[118] E. Timurdogan , C. V. Poulton , M. J. Byrd , and M. R. Watts , “Electric Field‐Induced Second‐Order Nonlinear Optical Effects in Silicon Waveguides,” Nature Photonics 11, no. 3 (2017): 200–206, 10.1038/nphoton.2017.14.

[119] J. Li , H. Zheng , Y. Han , et al., “Design and Analysis of Single‐Electrode Integrated Lithium Niobate Optical Phased Array for Two‐Dimensional Beam Steering,” Optics and Lasers in Engineering 184 (2025): 108617, 10.1016/j.optlaseng.2024.108617.

[120] C. Wang , M. Zhang , Xi Chen , et al., “Integrated Lithium Niobate Electro‐Optic Modulators Operating at CMOS‐Compatible Voltages,” Nature 562, no. 7725 (2018): 101–104, 10.1038/s41586-018-0551-y. 30250251

[121] U. Chakraborty , J. Carolan , G. Clark , et al., “Cryogenic Operation of Silicon Photonic Modulators Based on the DC Kerr Effect,” Optica 7, no. 10 (2020): 1385–1390, 10.1364/optica.403178.

[122] D. Lian , S. Zhao , W. Li , J. Chen , D. Dai , and Y. Shi , “Grating‐Lobe‐Free Optical Phased Array With 2‐D Circular Sparse Array Aperture and High‐Efficiency Phase Calibration,” Nanophotonics 13, no. 1 (2024): 29–37, 10.1515/nanoph-2023-0519. PMC11501881 39633994

[123] J. Chen , W. Li , D. Lian , S. Zhao , D. Dai , and Y. Shi , “Coaxial Transceiving LiDAR Based on a Silicon Photonic Optical Phased Array,” Optics Letters 49, no. 12 (2024): 3424–3427, 10.1364/ol.525376. 38875636

[124] Z. Wang , B. Wu , J. Liao , et al., “Calibration of 16 × 16 SOI Optical Phased Arrays via Improved SPGD Algorithm,” Optics Laser Technology 157 (2023): 108743, 10.1016/j.optlastec.2022.108743.

[125] J. Guo , W. Zhang , Z. Liao , C. Zhang , Yu Yu , and X. Zhang , “Multi‐Beam Top‐Facing Optical Phased Array Enabling a 360° Field of View,” Photonics Research 13, no. 4 (2025): 889–896, 10.1364/prj.543338.

[126] W. Ren , Q. Liang , J. Feng , et al., “Free‐Space Beam Shaping and Steering Based on a Silicon Optical Phased Array,” Photonics Research 11, no. 12 (2023): 2093–2099, 10.1364/prj.494546.

[127] D. Fowler , S. Guerber , J. Faugier‐Tovar , N. A. Tyler , and B. Szelag , “Integrated Optical Phased Array Based on a Binary Splitter Tree With Reduced Number of Control Voltages,” Journal of Lightwave Technology 40, no. 12 (2022): 4027–4032, 10.1109/jlt.2022.3154971.

[128] S. Guerber , D. Fowler , J. Faugier‐Tovar , K. A. Carim , B. Delplanque , and B. Szelag , “Wafer‐Level Calibration of Large‐Scale Integrated Optical Phased Arrays,” Optics Express 30, no. 20 (2022): 35246–35255, 10.1364/oe.464540. 36258480

[129] S.W. Chung , H. Abediasl , and H. Hashemi , “A Monolithically Integrated Large‐Scale Optical Phased Array in Silicon‐on‐Insulator CMOS,” IEEE Journal of Solid‐State Circuits 53, no. 1 (2017): 275–296, 10.1109/jssc.2017.2757009.

[130] K. Shang , C. Qin , Yu Zhang , et al., “Uniform Emission, Constant Wavevector Silicon Grating Surface Emitter for Beam Steering With Ultra‐Sharp Instantaneous Field‐of‐View,” Optics Express 25, no. 17 (2017): 19655–19661, 10.1364/oe.25.019655. 29041653

[131] S. A. Miller , Y.‐C. Chang , C. T. Phare , et al., “Large‐Scale Optical Phased Array Using a Low‐Power Multi‐Pass Silicon Photonic Platform,” Optica 7, no. 1 (2020): 3–6, 10.1364/optica.7.000003.

[132] W. Tong , Y. Wei , H. Zhou , J. Dong and X. Zhang . “The Design of a Low‐Loss, Fast‐Response, Metal Thermo‐Optic Phase Shifter Based on Coupled‐Mode Theory.” in Photonics, vol 9, no. 7 (MDPI, 2022), 447, 10.3390/photonics9070447.

[133] Y. Ye , D. Lian , Z. Chen , et al., “Visible‐Light Optical Phased Array on a Thin‐Film Lithium Niobate Platform for High‐Speed Beam Steering,” Optics Letters 50, no. 9 (2025): 3090–3093, 10.1364/ol.559117. 40310843

[134] K. Komatsu , Y. Kohno , Y. Nakano , and T. Tanemura , “Large‐Scale Monolithic InP‐Based Optical Phased Array,” IEEE Photonics Technology Letters 33, no. 20 (2021): 1123–1126, 10.1109/lpt.2021.3107277.

[135] Y. Liu , C. Zhang , D. M. DeSantis , et al., “High‐Resolution Arrayed Waveguide Grating‐Assisted Passive Optical Phased Array for 2D Beam Steering,” Optics Express 33, no. 4 (2025): 7714–7722, 10.1364/oe.549588. 40798486

[136] D. Maurya , D. Chack , and G. Vickey , “Compact and Efficient Transverse Spliced Waveguide Grating Antenna for Integrated Optical Phased Array,” IEEE Transactions on Nanotechnology (2024).

[137] W. Liu , K. Zhang , B. Li , et al., “Blue‐Green Wavelength Division Demultiplexing via Polarization‐Sensitive Optical Phased Array,” Journal of Lightwave Technology (2024).

[138] M. Nakai , I. Takai , S.W. Chung , and H. Hashemi , “Adaptive Beam Forming Across Temperature Variation in Optical Phased Array Enabled With Deep Neural Network,” Optics Express 32, no. 11 (2024): 18746–18760, 10.1364/oe.516722. 38859025

[139] A. Garcia Coleto , M. Notaros , and J. Notaros , “Visible‐Light Uniform and Unidirectional Grating‐Based Antennas for Integrated Optical Phased Arrays,” Optics Express 32, no. 26 (2024): 46447–46466, 10.1364/oe.540886.

[140] J. Duan , W. Yao , X. Zhao , et al., “Vertical Directional Coupling Based Grating Emission Engineering for Optical Phased Arrays,” Optics Letters 49, no. 11 (2024): 3102–3105, 10.1364/ol.524198. 38824338

[141] H. Farheen , A. Strauch , J. Christoph Scheytt , V. Myroshnychenko , and J. Förstner , “Optimized, Highly Efficient Silicon Antennas for Optical Phased Arrays,” Photonics and Nanostructures‐Fundamentals and Applications 58 (2024): 101207, 10.1016/j.photonics.2023.101207.

[142] S. Khajavi , J. Zhang , P. Cheben , et al., “Experimental Demonstration of a Silicon Nanophotonic Antenna for Far‐Field Broadened Optical Phased Arrays,” Photonics Research 12, no. 9 (2024): 1954–1961, 10.1364/prj.515222.

[143] M. Raval , C. V. Poulton , and M. R. Watts , “Unidirectional Waveguide Grating Antennas With Uniform Emission for Optical Phased Arrays,” Optics Letters 42, no. 13 (2017): 2563–2566, 10.1364/ol.42.002563. 28957285

[144] R. Marchetti , C. Lacava , L. Carroll , K. Gradkowski , and P. Minzioni , “Coupling Strategies for Silicon Photonics Integrated Chips,” Photonics Research 7, no. 2 (2019): 201–239, 10.1364/prj.7.000201.

[145] D. M. DeSantis , B. M. Mazur , M. Notaros , and J. Notaros , “Multi‐Beam Solid‐State LiDAR Using Star‐Coupler‐Based Optical Phased Arrays,” Optics Express 32, no. 21 (2024): 36656–36673, 10.1364/oe.537489. 39573551

[146] B. Chen , Y. Li , Q. Xie , et al., “SiN‐on‐SOI Optical Phased Array LiDAR for Ultra‐Wide Field of View and 4D Sensing,” Laser & Photonics Reviews 18, no. 10 (2024): 2301360, 10.1002/lpor.202301360.

[147] D. M. DeSantis , M. R. Torres , A. Garcia Coleto , et al., “Spiral Integrated Optical Phased Arrays for Tunable Near‐Field‐Focusing Emission,” Optics Express 32, no. 25 (2024): 44567–44580, 10.1364/oe.540171.

[148] M. Zadka , Y.‐C. Chang , A. Mohanty , C. T. Phare , S. P. Roberts , and M. Lipson , “On‐Chip Platform for a Phased Array With Minimal Beam Divergence and Wide Field‐of‐View,” Optics Express 26, no. 3 (2018): 2528–2534, 10.1364/oe.26.002528. 29401791

[149] B. Chen , Y. Li , L. Zhang , et al., “Unidirectional Large‐Scale Waveguide Grating With Uniform Radiation for Optical Phased Array,” Optics Express 29, no. 13 (2021): 20995–21010, 10.1364/oe.427999. 34266175

[150] Y. Zhang , R. Wang , M. Wei , G. Zhao , R. Jiang , and K. Du , “Design of High‐Efficiency and Large‐Field Silicon‐Based Transceiver Integrated Optical Phased Array,” Optics & Laser Technology 171 (2024): 110421, 10.1016/j.optlastec.2023.110421.

[151] Y. Zhang , R. Wang , H. Liu , et al., “Intensive and Efficient Design of a Two‐Dimensional 8 × 8 Silicon‐Based Optical Phased Array Transceiver,” Sensors 23, no. 23 (2023): 9396, 10.3390/s23239396. PMC10708612 38067769

[152] L.‐M. Leng , Y. Shao , P.‐Y. Zhao , G.‐F. Tao , S.‐N. Zhu , and W. Jiang , “Waveguide Superlattice‐Based Optical Phased Array,” Physical Review Applied 15, no. 1 (2021): 014019, 10.1103/physrevapplied.15.014019.

[153] D. Benedikovič , Q. Liu , A. Sánchez‐Postigo , et al., “Circular Optical Phased Array With Large Steering Range and High Resolution,” Sensors 22, no. 16 (2022): 6135, 10.3390/s22166135. PMC9412481 36015897

[154] P. Ginel‐Moreno , D. Pereira‐Martín , A. Hadij‐ElHouati , et al., “Highly Efficient Optical Antenna With Small Beam Divergence in Silicon Waveguides,” Optics Letters 45, no. 20 (2020): 5668–5671, 10.1364/ol.404012. 33057254

[155] P. Ginel‐Moreno , A. Sánchez‐Postigo , J. de Oliva‐Rubio , et al., “Millimeter‐Long Metamaterial Surface‐Emitting Antenna in the Silicon Photonics Platform,” Optics Letters 46, no. 15 (2021): 3733–3736, 10.1364/ol.431983. 34329268

[156] P. Ginel‐Moreno , A. Hadij‐ElHouati , A. Sánchez‐Postigo , et al., “On‐Chip Metamaterial Antenna Array With Distributed Bragg Deflector for Generation of Collimated Steerable Beams,” Laser & Photonics Reviews 16, no. 9 (2022): 2200164, 10.1002/lpor.202200164.

[157] Y. Jin , Q. Zhou , H. Li , et al., “Visible Optical Phased Array With Large Critical Dimensions for On‐Chip Fluorescence Sensing,” Journal of Lightwave Technology 43, no. 8 (2025): 3801–3807, 10.1109/jlt.2025.3529849.

[158] Y. Guo , Y. Guo , C. Li , X. Zhou , Z. Huang , and L. Zhang , “Bidirectional Wide‐Angle Waveguide Grating Antennas With Flat‐Top Far‐Field Patterns for Optical Phased Arrays,” Optics Express 31, no. 5 (2023): 9072–9080, 10.1364/oe.484362. 36860007

[159] J. Yoon , H. Yoon , J.‐Y. Kim , et al., “Demonstration of High‐Accuracy 3D Imaging Using a Si Optical Phased Array With a Tunable Radiator,” Optics Express 31, no. 6 (2023): 9935–9944, 10.1364/oe.481229. 37157557

[160] Y.‐J. Kwon , Y.‐Ho Sunwoo , Ji‐Y. Gwon , C.‐S. Im , and S.‐S. Lee , “On‐Chip Metalens‐Based Beamformer Enabling In‐Situ Beamforming in a Silicon Optical Phased Array,” Optics Express 33, no. 17 (2025): 35303–35313, 10.1364/oe.569162. 40984326

[161] X. Zhou , X. Yang , J. Jiang , et al., “Optical Phased Array Beam Scanning Technology Using Phased Fiber Laser Array Cascaded Microlens Array Scanners,” Journal of Lightwave Technology 43, no. 24 (2025): 11027–11036, 10.1109/jlt.2025.3618685.

[162] J. Sun , E. Timurdogan , A. Yaacobi , E. Shah Hosseini , and M. R. Watts , “Large‐Scale Nanophotonic Phased Array,” Nature 493, no. 7431 (2013): 195–199, 10.1038/nature11727. 23302859

[163] W. D. Sacher , Y. Huang , G.‐Q. Lo , and K. S. P. Joyce , “Multilayer Silicon Nitride‐on‐Silicon Integrated Photonic Platforms and Devices,” Journal of Lightwave Technology 33, no. 4 (2015): 901–910, 10.1109/jlt.2015.2392784.

[164] J. Sun , E. Timurdogan , A. Yaacobi , et al., “Large‐Scale Silicon Photonic Circuits for Optical Phased Arrays,” IEEE Journal of Selected Topics in Quantum Electronics 20, no. 4 (2013): 264–278, 10.1109/JSTQE.2013.2293316.

[165] Y. Ren , L. Zhang , W. Zhao , et al., “Compact 2D Serpentine Optical Phased Array,” Applied Optics 60, no. 24 (2021): 7158–7163, 10.1364/ao.431942. 34613002

[166] X. Yi , H. Zeng , S. Gao , and C. Qiu , “Design of an Ultra‐Compact Low‐Crosstalk Sinusoidal Silicon Waveguide Array for Optical Phased Array,” Optics Express 28, no. 25 (2020): 37505–37513, 10.1364/oe.405802. 33379583

[167] Y. Liu , X. Meng , and H. Hu , “1000‐Channel Integrated Optical Phased Array With 180° Field of View, High Resolution, and High Scalability,” Photonics Research 14, no. 4 (2026): 1035–1052, 10.1364/prj.567167.

[168] J. Shim , J.‐B. You , H.‐W. Rhee , et al., “On‐Chip Monitoring of Far‐Field Patterns Using a Planar Diffractor in a Silicon‐Based Optical Phased Array,” Optics Letters 45, no. 21 (2020): 6058–6061, 10.1364/ol.399263. 33137068

[169] R. Jiang , R. Wang , J. Guo , et al., “Design and Analysis of a Two‐Dimensional Large‐Scale Silicon‐Photonic Optical Phased Array,” Optics Laser Technology 156 (2022): 108551, 10.1016/j.optlastec.2022.108551.

[170] H. Zhang , Z. Zhang , J. Lv , C. Peng , and W. Hu , “Fast Beam Steering Enabled by a Chip‐Scale Optical Phased Array With 8 × 8 Elements,” Optics Communications 461 (2020): 125267, 10.1016/j.optcom.2020.125267.

[171] Y. Zhang , Y.‐C. Ling , K. Zhang , et al., “Sub‐Wavelength‐Pitch Silicon‐Photonic Optical Phased Array for Large Field‐of‐Regard Coherent Optical Beam Steering,” Optics Express 27, no. 3 (2019): 1929–1940, 10.1364/oe.27.001929. 30732239

[172] J.‐Y. Kim , S. Lee , S. Hong , et al., “Experimental Demonstration of Dual‐Polarization Multiplexed Optical Phased Array Empowered by Inverse Design,” Nanophotonics 14, no. 14 (2025): 2495–2505, 10.1515/nanoph-2025-0148. PMC12273540 40687571

[173] Z. Wang , Y. Sun , J. Liao , et al., “High‐Resolution Dynamic Imaging System Based on a 2D Optical Phased Array,” Optics Express 29, no. 24 (2021): 39342–39351, 10.1364/oe.439285. 34809300

[174] Y. Han , H. Zheng , B. Li , et al., “Loop Differential Ghost Imaging Based on Line Pattern,” Optics Communications 580 (2025): 131589, 10.1016/j.optcom.2025.131589.

[175] P. Lu , W. Xu , C. Zhu , et al., “Integrated Multi‐Beam Optical Phased Array Based on a 4 × 4 Butler Matrix,” Optics Letters 46, no. 7 (2021): 1566–1569, 10.1364/ol.419828. 33793487

[176] H. A. Clevenson , S. J. Spector , L. Benney , et al., “Incoherent Light Imaging Using an Optical Phased Array,” Applied Physics Letters 116, no. 3 (2020): 031105, 10.1063/1.5130697.

[177] H.‐W. Rhee , J.‐B. You , H. Yoon , et al., “32 Gbps Data Transmission With 2D Beam‐Steering Using a Silicon Optical Phased Array,” IEEE Photonics Technology Letters 32, no. 13 (2020): 803–806, 10.1109/lpt.2020.2998162.

[178] Y. Kim , L. Kulmer , K. Keller , et al., “Monolithically Integrated Optical Phased Array for Optical Wireless Communication,” Journal of Lightwave Technology (2024).

[179] S. Zhao , D. Lian , W. Li , J. Chen , D. Dai , and Y. Shi , “Low Sidelobe Silicon Optical Phased Array With Chebyshev Amplitude Distribution,” Nanophotonics 13, no. 3 (2024): 263–269, 10.1515/nanoph-2023-0507. PMC11501257 39633679

[180] A. Sharma , J. N. Straguzzi , T. Xue , et al., “Optimization of a Programmable *λ*/2‐Pitch Optical Phased Array,” Nanophotonics 13, no. 12 (2024): 2241–2249, 10.1515/nanoph-2023-0819. PMC11614342 39634504

[181] C. Chen , D. Kong , Y. Tao , et al., “Half‐Wavelength‐Pitch Silicon Optical Phased Array With a 180° Field of View, High Sidelobe Suppression Ratio, and Complex‐Pattern Beamforming,” Optica 11, no. 11 (2024): 1575–1582, 10.1364/optica.537785.

[182] L. Yu , P. Wang , P. Ma , et al., “Two‐Dimensional Beam Scanning of Passive Optical Phased Array Based on Silicon Nitride Delay Line,” Journal of Lightwave Technology 41, no. 9 (2023): 2756–2764, 10.1109/JLT.2023.3239002.

[183] C.‐S. Im , S.‐M. Kim , K.‐P. Lee , et al., “Hybrid Integrated Silicon Nitride–Polymer Optical Phased Array for Efficient Light Detection and Ranging,” Journal of Lightwave Technology 39, no. 13 (2021): 4402–4409, 10.1109/jlt.2021.3070386.

[184] C. V. Poulton , M. J. Byrd , M. Raval , et al., “Large‐Scale Silicon Nitride Nanophotonic Phased Arrays at Infrared and Visible Wavelengths,” Optics Letters 42, no. 1 (2016): 21–24, 10.1364/ol.42.000021. 28059212

[185] Z. Liu , N. L. Thomas , and R. Baets , “Broadband, High Resolution, Sensitive Spectrometer Using an Integrated Optical Phased Array in Silicon Nitride and Fourier Imaging,” Journal of Lightwave Technology (2024).

[186] C.‐S. Im , B. Bhandari , K.‐P. Lee , S.‐M. Kim , M.‐C. Oh , and S.‐S. Lee , “Silicon Nitride Optical Phased Array Based on a Grating Antenna Enabling Wavelength‐Tuned Beam Steering,” Optics Express 28, no. 3 (2020): 3270–3279, 10.1364/oe.383304. 32121999

[187] P. Muñoz , D. Pastor , L. A. Bru , et al., “Scalable Switched Slab Coupler Based Optical Phased Array on Silicon Nitride,” IEEE Journal of Selected Topics in Quantum Electronics 28, no. 5 (2022): 1–16, 10.1109/jstqe.2022.3162577.

[188] E.‐S. Lee , J. Jin , K.‐W. Chun , S.‐S. Lee , and M.‐C. Oh , “High‐Performance Optical Phased Array for LiDARs Demonstrated by Monolithic Integration of Polymer and SiN Waveguides,” Optics Express 31, no. 17 (2023): 28112–28121, 10.1364/oe.499868. 37710873

[189] W. Jiao , J. Chen , D. Lian , et al., “Two‐Dimensional Multi‐Beam Steering for Parallel Free‐Space Optical Communication Based on a Silicon Nitride Optical Phase Array,” APL Photonics 10, no. 5 (2025): 056110, 10.1063/5.0264068.

[190] X. Wang , P. Ji , Ze Zhang , G. Zhang , and S. Yang , “Chip‐Scale Optical Phased Array for Broadband Two‐Dimensional Beam Steering at Visible Wavelengths,” Optics & Laser Technology 181 (2025): 111615, 10.1016/j.optlastec.2024.111615.

[191] Q. Zhang , D. Lian , Z. Xu , et al., “Four Directional Circular Optical Phased Array for Uniform Two‐Dimensional Beam Steering at Visible Wavelength,” Optics Express 33, no. 7 (2025): 15954–15963, 10.1364/oe.559081. 40219495

[192] X. Yu , Z. Wu , J. Zeng , S. Lin , X. Cai , and Y. Zhang , “532 nm Silicon Nitride Optical Phased Array and High‐Speed Calibration and Control System,” Chinese Optics Letters 23, no. 6 (2025): 061301, 10.3788/col202523.061301.

[193] Y. Fu , B. Chen , W. Yue , et al., “Target‐Adaptive Optical Phased Array Lidar,” Photonics Research 12, no. 5 (2024): 904–911, 10.1364/prj.514468.

[194] D. S. Wesley , F.‐D. Chen , H. Moradi‐Chameh , et al., “Optical Phased Array Neural Probes for Beam‐Steering in Brain Tissue,” Optics Letters 47, no. 5 (2022): 1073–1076, 10.1364/OL.441609. 35230293

[195] S. Arsanjani , M. S. Khan , J. Bardong , A. Frank , L. Zimmermann , and C. Tommaso , “Two Dimensional Beam Forming and Steering Based on Silicon Nitride 1 × 32 Optical Phased Array,” Optics Express 32, no. 25 (2024): 43778–43787, 10.1364/oe.534684.

[196] P. Wang , A. Kazemian , X. Zeng , Y. Zhuang , and Y. Yi , “Optimization of Aperiodic 3D Optical Phased Arrays Based on Multilayer Si3N4/SiO2 Platforms,” Applied Optics 60, no. 3 (2021): 484–491, 10.1364/ao.411718. 33690419

[197] Li Yang , H. Chen , R. Liu , et al., “Design of Optical Phased Array With Low‐Sidelobe Beam Steering in Thin Film Lithium Niobate,” Optics Laser Technology 171 (2024): 110432, 10.1016/j.optlastec.2023.110432.

[198] P. Wang , G. Luo , Y. Xu , et al., “Design and Fabrication of a SiN‐Si Dual‐Layer Optical Phased Array Chip,” Photonics Research 8, no. 6 (2020): 912–919, 10.1364/prj.387376.

[199] H. Hu , Y. He , B. Chen , et al., “Silicon‐Based Optical Phased Array With a Reconfigurable Aperture for “Gaze” Scanning of LiDAR,” Photonics Research 12, no. 5 (2024): 932–940, 10.1364/prj.515496.

[200] L. Zhang , Y. Li , Yu Hou , et al., “Investigation and Demonstration of a High‐Power Handling and Large‐Range Steering Optical Phased Array Chip,” Optics Express 29, no. 19 (2021): 29755–29765, 10.1364/oe.434067. 34614714

[201] Q. Wang , S. Wang , L. Jia , Y. Cai , W. Yue , and M. Yu , “Silicon Nitride Assisted 1 × 64 Optical Phased Array Based on a SOI Platform,” Optics Express 29, no. 7 (2021): 10509–10517, 10.1364/oe.420921. 33820184

[202] Y. Li , B. Chen , Q. Na , et al., “High‐Data‐Rate and Wide‐Steering‐Range Optical Wireless Communication via Nonuniform‐Space Optical Phased Array,” Journal of Lightwave Technology 41, no. 15 (2023): 4933–4940, 10.1109/jlt.2023.3252166.

[203] J. Øyvind Kjellman , M. Prost , A. Marinins , et al., “Densely Integrated Phase Interrogators for Low‐Complexity on‐Chip Calibration of Optical Phased Arrays,” Journal of Lightwave Technology 40, no. 16 (2022): 5660–5667, 10.1109/jlt.2022.3179437.

[204] M. Prost , J. Ø. Kjellman , S. Dwivedi , et al., “Optical Phased Array With on‐Chip Phase Calibration,” Optics Letters 47, no. 23 (2022): 6081–6084, 10.1364/ol.467779. 37219177

[205] Q. Wang , S. Wang , L. Jia , Y. Cai , W. Yue , and M. Yu , “Increasing Wavelength‐Controlled Steering Range of An Optical Phased Array by Using Two Subarrays,” Applied Optics 60, no. 18 (2021): 5424–5429, 10.1364/ao.422721. 34263782

[206] L. Zhang , Y. Li , B. Chen , et al., “Two‐Dimensional Multi‐Layered SiN‐on‐SOI Optical Phased Array With Wide‐Scanning and Long‐Distance Ranging,” Optics Express 30, no. 4 (2022): 5008–5018, 10.1364/oe.446733. 35209472

[207] R. S. Weis and T. K. Gaylord , “Lithium Niobate: Summary of Physical Properties and Crystal Structure,” Applied Physics A 37, no. 4 (1985): 191–203, 10.1007/bf00614817.

[208] P. Kharel , C. Reimer , K. Luke , L. He , and M. Zhang , “Breaking Voltage–Bandwidth Limits in Integrated Lithium Niobate Modulators Using Micro‐Structured Electrodes,” Optica 8, no. 3 (2021): 357–363, 10.1364/optica.416155.

[209] M. Xu , Y. Zhu , F. Pittalà , et al., “Dual‐Polarization Thin‐Film Lithium Niobate in‐Phase Quadrature Modulators for Terabit‐Per‐Second Transmission,” Optica 9, no. 1 (2022): 61–62, 10.1364/optica.449691.

[210] Y. Li , S. Deng , X. Ma , et al., “Narrow Beam and Low‐Sidelobe Electro‐Optic Beam Steering on Thin‐Film Lithium Niobate Optical Phased Array,” Opto‐Electronic Science 5, no. 2 (2026): 260002–1, 10.29026/oes.2026.260002.

[211] X. Ma , M. Yuan , J. Li , et al., “Multi‐Target and Ultra‐High‐Speed Optical Wireless Communication Using a Thin‐Film Lithium Niobate Optical Phased Array,” Nature Communications (2025). 10.1038/s41467-025-67696-3 PMC12847847 41397980

[212] J. Li , H. Zheng , Y. He , Y. Liu , X. Wei , and Z. Xu , “Cascaded Domain Engineering Optical Phased Array for 2D Beam Steering,” Nanophotonics 12, no. 21 (2023): 4017–4030, 10.1515/nanoph-2023-0382. PMC11501413 39635637

[213] Z. Wang , J. Ji , X. Ye , et al., “On‐Chip Integration of Metasurface‐Doublet for Optical Phased Array With Enhanced Beam Steering,” Nanophotonics 12, no. 13 (2023): 2425–2432, 10.1515/nanoph-2022-0697. PMC11501765 39633745

[214] Z. Wang , W. Song , Y. Chen , et al., “Metasurface Empowered Lithium Niobate Optical Phased Array With an Enlarged Field of View,” Photonics Research 10, no. 11 (2022): B23–B29, 10.1364/prj.463118.

[215] M. Gagino , M. B. J. Van Rijn , E. A. J. M. Bente , M. K. Smit , and V. Dolores‐Calzadilla , “Broadband Operation of an InP Optical Phased Array,” IEEE Photonics Technology Letters 34, no. 10 (2022): 541–544, 10.1109/lpt.2022.3171979.

[216] B. S. Vikram , M. Gagino , A. Millan‐Mejia , L. Augustin , K. A. Williams , and V. D. Calzadilla , “Demonstration of Inherently Low Differential Phase Noise Across C‐Band in InP Integrated, Amplifying Optical Phased Arrays,” IEEE Journal of Quantum Electronics 60, no. 6 (2024): 1–7, 10.1109/jqe.2024.3404009.

[217] Su Tan , X. Dai , J. Lou , et al., “Low‐Power Consumption InP‐Based Optical Phase Arrays With Non‐Uniformly Spaced Output Waveguides,” Optics Express 31, no. 2 (2023): 3199–3211, 10.1364/oe.479334. 36785317

[218] J. Midkiff , K. M. Yoo , J.‐D. Shin , H. Dalir , M. Teimourpour , and R. T. Chen , “Optical Phased Array Beam Steering in the Mid‐Infrared on an InP‐Based Platform,” Optica 7, no. 11 (2020): 1544–1547, 10.1364/optica.400441.

[219] J. Midkiff , P.‐Y. Hsiao , P. T. Camp , and R. T. Chen , “Mid‐Infrared 2D Nonredundant Optical Phased Array of Mirror Emitters in an InGaAs/InP Platform,” Optics Express 32, no. 24 (2024): 43329–43341, 10.1364/oe.533285.

[220] S.‐I. Kuo , J.‐W. Wang , Z. Ahmad , et al., “Reconfigurable Scan Lens Based on an Actively Controlled Optical Phased Array,” Optics Letters 47, no. 15 (2022): 3676–3679, 10.1364/ol.461697. 35913287

[221] M. Nickerson , B. Song , J. Brookhyser , G. Erwin , J. Kleinert , and J. Klamkin , “Gallium Arsenide Optical Phased Array Photonic Integrated Circuit,” Optics Express 31, no. 17 (2023): 27106–27122, 10.1364/oe.492556. 37710792

[222] Y. Miyamoto , M. Miura , J. Shibasaki , et al., “Optical Phased Array With Dual‐Layer Electro‐Optic Polymer and Silicon Nitride Waveguides,” IEEE Photonics Journal 18, no. 1 (2025): 1–8, 10.1109/jphot.2025.3639001.

[223] A. Yaacobi , J. Sun , M. Moresco , G. Leake , D. Coolbaugh , and M. R. Watts , “Integrated Phased Array for Wide‐Angle Beam Steering,” Optics Letters 39, no. 15 (2014): 4575–4578, 10.1364/ol.39.004575. 25078232

[224] C. V. Poulton , M. J. Byrd , B. Moss , E. Timurdogan , R. Millman , and M. R. Watts , “8192‐Element Optical Phased Array With 100° Steering Range and Flip‐CHIP CMOS,” in CLEO: QELS_Fundamental Science (Optical Society of America, 2020): JTh4A–3.

[225] J. A. Thomas and Y. Fainman , “Optimal Cascade Operation of Optical Phased‐Array Beam Deflectors,” Applied Optics 37, no. 26 (1998): 6196–6212, 10.1364/ao.37.006196. 18286118

[226] S. Zhu , Yu Li , T. Hu , et al., “Silicon Nitride Optical Phased Arrays With Cascaded Phase Shifters for Easy and Effective Electronic Control,” in CLEO: Applications and Technology, AW4K–5 (Optica Publishing Group, 2019).

[227] M. T. I. Badal , J. Scott , and K. Wang , “Multimode Optical Phased Array for Parallel Beam Steering–Feasibility Study,” Optics Express 31, no. 12 (2023): 18907–18930, 10.1364/oe.484937. 37381321

[228] H. Hu , W. Yue , J. Li , et al., “Row‐Gated Optical Phased Array Using Integrated Diodes for 3D Structured Light Imaging,” Optica 12, no. 6 (2025): 789–798, 10.1364/optica.559206.

[229] A. D. Ushakov , J. Li , M. A. Chuvakova , et al., “Cascaded Optical Phased Array for Beam Steering Created by Domain Engineering in PMN–PT Single Crystal,” Bulletin of the Russian Academy of Sciences: Physics 89, no. S3 (2025): 1–7, 10.1134/s1062873825714734.

[230] D. Kwong , A. Hosseini , J. Covey , et al., “Corrugated Waveguide‐Based Optical Phased Array With Crosstalk Suppression,” IEEE Photonics Technology Letters 26, no. 10 (2014): 991–994, 10.1109/lpt.2014.2311454.

[231] G. Zhou , S.‐W. Qu , J. Wu , and S. Yang , “Design of a Low‐Crosstalk Sub‐Wavelength‐Pitch Silicon Waveguide Array for Optical Phased Array,” IEEE Photonics Journal 13, no. 4 (2021): 1–8, 10.1109/jphot.2021.3103392.

[232] L. Li , H.‐N. Xiong , X. Li , et al., “Crosstalk Reduction for Arrayed Waveguide Gratings on Silicon‐on‐Insulator Platform,” Optics Laser Technology 175 (2024): 110817, 10.1016/j.optlastec.2024.110817.

[233] S. Jahani and Z. Jacob , “Transparent Subdiffraction Optics: Nanoscale Light Confinement Without Metal,” Optica 1, no. 2 (2014): 96–100, 10.1364/optica.1.000096.

[234] Ziyun K. , “Design and Characterization of Optical Phased Array With Half‐Wavelength Spacing.” (PhD diss., Purdue University, 2021).

[235] M. Faiyaz Kabir , M. Borhan Mia , I. Ahmed , N. Jaidye , S. Z. Ahmed , and S. Kim , “Anisotropic Leaky‐Like Perturbation With Subwavelength Gratings Enables Zero Crosstalk,” Light: Science Applications 12, no. 1 (2023): 135, 10.1038/s41377-023-01184-5. PMC10238401 37268648

[236] L. Wang , Z. Chen , H. Wang , et al., “Design of a Low‐Crosstalk Half‐Wavelength Pitch Nano‐Structured Silicon Waveguide Array,” Optics Letters 44, no. 13 (2019): 3266–3269, 10.1364/ol.44.003266. 31259937

[237] Y. Yang , Y. Guo , Y. Huang , et al., “Crosstalk Reduction of Integrated Optical Waveguides With Nonuniform Subwavelength Silicon Strips,” Scientific Reports 10, no. 1 (2020): 4491, 10.1038/s41598-020-61149-1. PMC7066159 32161297

[238] A. Debevc , M. Topič , and K. Janez , “Increasing Integration Density of Photonic Integrated Circuits by Employing Optimized Dielectric Metamaterial Structures,” IEEE Photonics Journal 13, no. 6 (2021): 1–9, 10.1109/jphot.2021.3124626.

[239] K. Amin , L. Chrostowski , Z. Lu , and R. Bojko , “Significant Crosstalk Reduction Using All‐Dielectric CMOS‐Compatible Metamaterials,” IEEE Photonics Technology Letters 28, no. 24 (2016): 2787–2790, 10.1109/LPT.2016.2623349.

[240] S. Jahani , S. Kim , J. Atkinson , et al., “Controlling Evanescent Waves Using Silicon Photonic All‐Dielectric Metamaterials for Dense Integration,” Nature Communications 9, no. 1 (2018): 1893, 10.1038/s41467-018-04276-8. PMC5951946 29760394

[241] C. Chen , X. Zhao , S. Tang , X. Liu , and H. Lv , “Realization of Low‐Cross‐Talk Half‐Wavelength Pitch Waveguide Array on a Silicon‐on‐Insulator Platform,” Optics Letters 47, no. 12 (2022): 2955–2958, 10.1364/ol.455977. 35709024

[242] A. Sharma , J. N. Straguzzi , T. Xue , et al., “Crosstalk‐Compensated Optical Phased Arrays for Wide‐Angle Beam‐Steering,” in Optical Fiber Communication Conference, Th2A–3 (Optica Publishing Group, 2024).

[243] Ye Yuan , C. Jiang , M. Wu , and J. Zhu , “Non‐Uniform Optical Phased Array Based on Dual‐Adaption Genetic Algorithm Improved by Chaos Sequence,” Optics and Lasers in Engineering 183 (2024): 108500, 10.1016/j.optlaseng.2024.108500.

[244] D. Liang , W. Li , X. Wang , et al., “Grating Lobe‐Free Silicon Optical Phased Array With Periodically Bending Modulation of Dense Antennas,” Optics Express 31, no. 7 (2023): 11423–11430, 10.1364/oe.479150. 37155777

[245] Q. Huang , H. Yu , Z. Zhang , et al., “Sparse 2‐D Optical Phased Array With Large Grating‐Lobe‐Free Steering Range Based on an Aperiodic Grid,” Optics Letters 48, no. 11 (2023): 2849–2852, 10.1364/ol.488891. 37262226

[246] A. Khachaturian , R. Fatemi , and A. Hajimiri , “Achieving Full Grating‐Lobe‐Free Field of View With Low‐Complexity Co‐Prime Photonic Beamforming Transceivers,” Photonics Research 10, no. 5 (2022): A66–A73, 10.1364/prj.437518.

[247] D. Zhang , F. Zhang , and S. Pan , “Grating‐Lobe‐Suppressed Optical Phased Array With Optimized Element Distribution,” Optics Communications 419 (2018): 47–52, 10.1016/j.optcom.2018.03.007.

[248] L. P. Julián , I. Aldaya , O. J. S. Santana , L. E. E. De Araujo , P. Dainese , and L. H. Gabrielli , “Side‐Lobe Level Reduction in Bio‐Inspired Optical Phased‐Array Antennas,” Optics Express 25, no. 24 (2017): 30105–30114, 10.1364/OE.25.030105. 29221044

[249] V. Kakdarvishi , “Two‐Layer Optical Phased Array With Effective Subwavelength Pitch for Grating‐Lobe‐Free Beam Steering,” Optics Express 34, no. 3 (2026): 4115–4126, 10.1364/oe.582411. 41715462

[250] Z. Gao , X. Yang , Q. Zhou , et al., “Evanescent Field‐Modulated Non‐Uniform Optical Phased Array With Large Lateral Steering Angle,” Optics Express 34, no. 6 (2026): 10363–10373, 10.1364/oe.589077. 42071413

[251] Z. Zang , J. Wu , Q. Huang , and X. Zhang , “Holistic Design of Optical Phased Arrays With Wide Beam Scanning Range and High Side Mode Suppression Ratio Using an Improved Nutcracker Optimization Algorithm,” Optics and Lasers in Engineering 194 (2025): 109220, 10.1016/j.optlaseng.2025.109220.

[252] Y. Lei , L. Zhang , Y. Xue , et al., “Suppressing Grating Lobes of Large‐Aperture Optical Phased Array With Circular Array Design,” Applied Optics 62, no. 15 (2023): 4110–4114, 10.1364/ao.488916. 37706724

[253] F. Zhang , D. Zhang , and S. Pan , “Fast and Wide‐Range Optical Beam Steering With Ultralow Side Lobes by Applying an Optimized Multi‐Circular Optical Phased Array,” Applied Optics 57, no. 18 (2018): 4977–4984, 10.1364/ao.57.004977. 30117954

[254] B. Zhang , Yu Liu , Z. Zhao , and P. Yan , “Multi‐Beam Steering With Low Grating Lobes Using Optimized Unequally Spaced Phased Array,” Optics Communications 427 (2018): 48–53, 10.1016/j.optcom.2018.06.035.

[255] T. Komljenovic , R. Helkey , L. Coldren , and J. E. Bowers , “Sparse Aperiodic Arrays for Optical Beam Forming and LIDAR,” Optics Express 25, no. 3 (2017): 2511–2528, 10.1364/oe.25.002511. 29519096

[256] T. Dong , J. He , X. He , Y. Xu , and J. Zhou , “Hybrid Design Approach of Optical Phased Array With Wide Beam Steering Range and Low Side‐Lobe Level,” Optics Letters 47, no. 4 (2022): 806–809, 10.1364/ol.449369. 35167530

[257] J. Liu , B. Wang , J. Wang , et al., “Taylor Amplitude Distribution for Low Sidelobe in Thin‐Film Lithium Niobate Optical Phased Array,” Journal of Lightwave Technology (2025).

[258] D. Yang , C. Wang , H. Hu , et al., “A Vernier Optical Phased Array With Quasi Gaussian Intensity Distribution for Wide Field of View and High Sidelobe Suppression,” Optics Express 34, no. 5 (2026): 8698–8713, 10.1364/oe.583406. 41845811

[259] G. Zhou , S.‐W. Qu , and J. Wu , “Grating Lobe Suppression in Optical Phased Arrays by Loading Near‐Wavelength Grating,” Optics Letters 45, no. 20 (2020): 5664–5667, 10.1364/ol.401484. 33057253

[260] T. Xue , Y. Jung , J. N. Straguzzi , et al., “Single‐Lobe Steering of Blue Light With an Active Optical Phased Array,” Optics Letters 50, no. 16 (2025): 5177–5180, 10.1364/ol.569055. 40815769

[261] N. Dostart , B. Zhang , M. Brand , et al., “Vernier Optical Phased Array LiDAR Transceivers,” Optics Express 30, no. 14 (2022): 24589–24601, 10.1364/oe.451578. 36237010

[262] Y. Liu and H. Hu , “Integrated Optical Phased Array With 180‐Degree Field of View for Solid‐State 2D Optical Beam Steering,” Journal of Lightwave Technology (2025).

[263] J. A. Smith , D. Jevtics , B. Guilhabert , M. D. Dawson , and M. J. Strain , “Hybrid Integration of Chipscale Photonic Devices Using Accurate Transfer Printing Methods,” Applied Physics Reviews 9, no. 4 (2022): 041317, 10.1063/5.0121567.

[264] Di Liang , G. Roelkens , R. Baets , and J. E. Bowers , “Hybrid Integrated Platforms for Silicon Photonics,” Materials 3, no. 3 (2010): 1782–1802, 10.3390/ma3031782.

[265] M. Gagino , A. Millan‐Mejia , L. Augustin , K. Williams , E. Bente , and V. Dolores‐Calzadilla , “Integrated Optical Phased Array With On‐Chip Amplification Enabling Programmable Beam Shaping,” Scientific Reports 14, no. 1 (2024): 9590, 10.1038/s41598-024-60204-5. PMC11053068 38671206

[266] W. Xie , T. Komljenovic , J. Huang , et al., “Heterogeneous Silicon Photonics Sensing for Autonomous Cars,” Optics express 27, no. 3 (2019): 3642–3663, 10.1364/oe.27.003642. 30732381

[267] A. Rahim , A. Hermans , B. Wohlfeil , et al., “Taking Silicon Photonics Modulators to a Higher Performance Level: State‐of‐the‐Art and a Review of New Technologies,” Advanced Photonics 3, no. 2 (2021): 024003–024003, 10.1117/1.ap.3.2.024003.

[268] G. Kang , S.‐H. Kim , J.‐B. You , et al., “Silicon Based Optical Phased Array Using Electro‐Optic *p*‐*i*‐*n* Phase Shifters,” IEEE Photonics Technology Letters 31, no. 21 (2019): 1685–1688, 10.1109/LPT.2019.2939550.

[269] Z. Zhang , H. Yu , Q. Huang , et al., “High‐Speed and Low‐Power Silicon Optical Phased Array Based on the Carrier‐Depletion Mechanism,” IEEE Photonics Technology Letters 34, no. 5 (2022): 271–274, 10.1109/lpt.2022.3151059.

[270] J. Chen , D. Lian , S. Zhao , et al., “High‐Power‐Compatible Thick Silicon‐on‐Insulator Waveguide: An Implementation in Optical Phased Array,” IEEE Photonics Journal 18, no. 1 (2026): 1–6, 10.1109/jphot.2026.3652317.

[271] Y. Yi , D. Wu , V. Kakdarvishi , B. Yu , Y. Zhuang , and A. Khalilian , “Photonic Integrated Circuits for an Optical Phased Array,” Photonics 11, no. 3 (2024): 243: MDPI, 10.3390/photonics11030243.

[272] J. Notaros , C. V. Poulton , M. Raval , and M. R. Watts , “Near‐Field‐Focusing Integrated Optical Phased Arrays,” Journal of Lightwave Technology 36, no. 24 (2018): 5912–5920, 10.1109/jlt.2018.2880462.

[273] X. Yan , F. Li , H. Wang , et al., “Coherent Synthesis of Silicon Optical Phased Arrays Toward Enhanced Power and Aperture,” ACS Photonics (2026).

[274] D. N. Hutchison , J. Sun , J. K. Doylend , et al., “High‐Resolution Aliasing‐Free Optical Beam Steering,” Optica 3, no. 8 (2016): 887–890, 10.1364/optica.3.000887.

[275] J. Kim , Y. J. Lee , J.‐Y. Kim , et al., “High‐Efficiency Thermo‐Optic Control of a Longitudinal Radiation Angle in a Silicon‐Based Optical Phased Array by Leveraging the Backside Air Trench Structure,” Optics Letters 49, no. 15 (2024): 4338–4341, 10.1364/ol.524469. 39090928

[276] S. Zhao , D. Lian , J. Chen , D. Dai , and Y. Shi , “Polarization Multiplexing Silicon Photonic Optical Phased Array With a Wide Scanning Range,” Optics Letters 48, no. 23 (2023): 6092–6095, 10.1364/ol.504336. 38039199

[277] Y. Liu and H. Hu , “Integrated Optical Phased Array With 180‐Degree Field of View for Solid‐State 2D Optical Beam Steering,” Journal of Lightwave Technology (2025), 10.1109/JLT.2025.3557369.

[278] Z. Zhang , M. Notaros , Z. Gao , U. Chakraborty , J. Notaros , and D. S. Boning , “Impact of Process Variations on Splitter‐Tree‐Based Integrated Optical Phased Arrays,” Optics Express 31, no. 8 (2023): 12912–12921, 10.1364/oe.487096. 37157440

[279] Y. Zhang , R. Wang , M. Wei , R. Jiang , H. Zhang , and J.‐S. Huang , “Double‐Layer Silicon‐Based Optical Phased Array Transceiver Based on Cascaded Phase Control,” Scientific Reports 15, no. 1 (2025): 35996, 10.1038/s41598-025-19751-8. PMC12528711 41093947

[280] J. Jin , E.‐Su Lee , K.‐W. Chun , S.‐S. Lee , and M.‐C. Oh , “Practical In‐Situ Beamforming Method for Optical Phased Array LiDAR Monitoring a Single Far‐Field Point,” Journal of Lightwave Technology 43, no. 2 (2025): 589–595, 10.1109/jlt.2024.3457948.

[281] W. Bogaerts and L. Chrostowski , “Silicon Photonics Circuit Design: Methods, Tools and Challenges,” Laser Photonics Reviews 12, no. 4 (2018): 1700237, 10.1002/lpor.201700237.

[282] Yu Sun , F. Liu , and H. Xue , “High‐Speed and High‐Density Optoelectronic Co‐Package Technologies,” ZTE Technology Journal 24, no. 4 (2018): 27–32, 10.3969/j.issn.1009-6868.2018.04.005.

[283] L. Liao , S. Fathololoumi , and D. Hui , “High Density Silicon Photonic Integrated Circuits and Photonic Engine for Optical Co‐Packaged Ethernet Switch,” in 2020 European Conference on Optical Communications (ECOC) (IEEE, 2020), 1–4.

[284] R. Mahajan , X. Li , J. Fryman , et al., “Co‐Packaged Photonics for High Performance Computing: Status, Challenges and Opportunities,” Journal of Lightwave Technology 40, no. 2 (2021): 379–392, 10.1109/jlt.2021.3104725.

[285] CPO JDF , “Co‐Packaged Optical Module Discussion Document.” V1. 0.

[286] H. Li , G. Balamurugan , T. Kim , et al., “A 3‐D‐Integrated Silicon Photonic Microring‐Based 112‐Gb/s PAM‐4 Transmitter With Nonlinear Equalization and Thermal Control,” IEEE Journal of Solid‐State Circuits 56, no. 1 (2020): 19–29, 10.1109/jssc.2020.3022851.

[287] M. Tan , J. Xu , S. Liu , et al., “Co‐Packaged Optics (CPO): Status, Challenges, and Solutions,” Frontiers of Optoelectronics 16, no. 1 (2023): 1, 10.1007/s12200-022-00055-y. PMC10027985 36939942

[288] S. Agarwal , M. Ingels , M. Pantouvaki , M. Steyaert , P. Absil , and J. Van Campenhout , “Wavelength Locking of a Si Ring Modulator Using an Integrated Drop‐Port OMA Monitoring Circuit,” IEEE Journal of Solid‐State Circuits 51, no. 10 (2016): 2328–2344, 10.1109/jssc.2016.2592691.

[289] S. Grillanda , M. Carminati , F. Morichetti , et al., “Wavelength Locking of a Silicon Microring Resonator Assisted by ContactLess Integrated Photonic Probe,” in 11th International Conference on Group IV Photonics (GFP) (IEEE, 2014), 124–125.

[290] C. Sun , M. Wade , M. Georgas , et al., “A 45 nm CMOS‐SOI Monolithic Photonics Platform With Bit‐Statistics‐Based Resonant Microring Thermal Tuning,” IEEE Journal of Solid‐State Circuits 51, no. 4 (2016): 893–907, 10.1109/jssc.2016.2519390.

[291] Y. Thonnart , M. Zid , J. Luis Gonzalez‐Jimenez , et al., “A 10Gb/s Si‐Photonic Transceiver With 150*μ*W 120*μ*s‐Lock‐Time Digitally Supervised Analog Microring Wavelength Stabilization for 1Tb/s/mm 2 Die‐to‐Die Optical Networks,” in 2018 IEEE International Solid‐State Circuits Conference‐(ISSCC) (IEEE, 2018), 350–352.

[292] Z. Wang , Yu Y. , Xi Xiao , et al., “A Time‐Division‐Multiplexing Scheme for Simultaneous Wavelength Locking of Multiple Silicon Micro‐Rings,” in 2018 IEEE International Symposium on Circuits and Systems (ISCAS) (IEEE, 2018), 1–4.

[293] M.‐H. Kim , L. Zimmermann , and W.‐Y. Choi , “A Temperature Controller IC for Maximizing Si Micro‐Ring Modulator Optical Modulation Amplitude,” Journal of Lightwave Technology 37, no. 4 (2019): 1200–1206, 10.1109/jlt.2018.2889899.

[294] M. Kim , M.‐H. Kim , Y. Jo , et al., “A Fully Integrated 25 Gb/s Si Ring Modulator Transmitter With a Temperature Controller,” in 2020 Optical Fiber Communications Conference and Exhibition (OFC) (2020), 1–3.

[295] Da Ming , Z. Wang , Y. Wang , and M. Tan , “First Demonstration of Closed‐Loop PWM Wavelength Locking of a Microring Resonator in a Monolithic Photonic‐BiCMOS Platform,” in 2020 IEEE International Conference on Integrated Circuits, Technologies and Applications (ICTA) (IEEE, 2020), 163–164.

[296] H. Li , Z. Xuan , A. Titriku , et al., “A 25 Gb/s, 4.4 V‐Swing, AC‐Coupled Ring Modulator‐Based WDM Transmitter With Wavelength Stabilization in 65 nm CMOS,” IEEE Journal of Solid‐State Circuits 50, no. 12 (2015): 3145–3159, 10.1109/jssc.2015.2470524.

[297] S. Pornpromlikit , J. Jeong , C. D. Presti , A. Scuderi , and P. M. Asbeck , “A Watt‐Level Stacked‐FET Linear Power Amplifier in Silicon‐on‐Insulator CMOS,” IEEE Transactions on Microwave Theory and Techniques 58, no. 1 (2009): 57–64, 10.1109/TMTT.2009.2036323.

[298] H.‐T. Dabag , B. Hanafi , F. Golcuk , A. Agah , J. F. Buckwalter , and P. M. Asbeck , “Analysis and Design of Stacked‐FET Millimeter‐Wave Power Amplifiers,” IEEE Transactions on Microwave Theory and Techniques 61, no. 4 (2013): 1543–1556, 10.1109/tmtt.2013.2247698.

[299] K. Kim , K. Lee , G. Shin , et al., “A 50‐Gb/s Compact RadCom E‐Band Transmitter With Phase‐Controlled Push–Push Quadrupler and Stacked‐FET Power Amplifier,” IEEE Solid‐State Circuits Letters 4 (2021): 150–153, 10.1109/lssc.2021.3108486.

[300] Z. Xing , A. Samani , M. Xiang , et al., “100 Gb/s PAM4 Transmission System for Datacenter Interconnects Using a SiP ME‐MZM Based DAC‐Less Transmitter and a VSB Self‐Coherent Receiver,” Optics express 26, no. 18 (2018): 23969–23979, 10.1364/oe.26.023969. 30184891

[301] Q. Liao , N. Qi , M. Li , et al., “A 50‐Gb/s PAM4 Si‐Photonic Transmitter With Digital‐Assisted Distributed Driver and Integrated CDR in 40‐nm CMOS,” IEEE Journal of Solid‐State Circuits 55, no. 5 (2020): 1282–1296, 10.1109/jssc.2020.2967560.

[302] P. Rito , I. Garcia Lopez , A. Ahmed , M. Ko , A. C. Ulusoy , and D. Kissinger , “A DC‐90‐GHz 4‐Vpp Modulator Driver in a 0.13‐*μ*m SiGe: C BiCMOS Process,” IEEE Transactions on Microwave Theory and Techniques 65, no. 12 (2017): 5192–5202, 10.1109/TMTT.2017.2757927.

[303] J. He , Y. Zhang , H. Liu , et al., “A 56‐Gb/s Reconfigurable Silicon‐Photonics Transmitter Using High‐Swing Distributed Driver and 2‐Tap in‐Segment Feed‐Forward Equalizer in 65‐nm CMOS,” IEEE Transactions on Circuits and Systems I: Regular Papers 69, no. 3 (2021): 1159–1170, 10.1109/tcsi.2021.3127723.

[304] Q. Liao , Y. Zhang , S. Ma , et al., “A 50‐Gb/s PAM‐4 Silicon‐Photonic Transmitter Incorporating Lumped‐Segment MZM, Distributed CMOS Driver, and Integrated CDR,” IEEE Journal of Solid‐State Circuits 57, no. 3 (2021): 767–780, 10.1109/jssc.2021.3134874.

[305] A. Lukashchuk , H. K. Yildirim , A. Bancora , et al., “Photonic‐Electronic Integrated Circuit‐Based Coherent LiDAR Engine,” Nature Communications 15, no. 1 (2024): 3134, 10.1038/s41467-024-47478-z. PMC11009237 38605067

[306] Z. Zhou , W. Xu , C. Liu , et al., “Butler Matrix Enabled Multi‐Beam Optical Phased Array for Two‐Dimensional Beam‐Steering and Ranging,” Photonics Research 12, no. 5 (2024): 912–920, 10.1364/prj.509595.

[307] J. Yuan , G. Wu , S. Yang , et al., “Angle Measurement Using the Integrated Amplitude of Difference Beam From an Optical Phased Array,” Optics Express 33, no. 6 (2025): 13785–13795, 10.1364/oe.554236. 40798183

[308] J. Jin , E.‐S. Lee , K.‐W. Chun , and M.‐C. Oh , “Coherent Line‐Beam LiDARs Using Polymer Waveguide Optical Phased Array,” Optics Express 34, no. 5 (2026): 9158–9165, 10.1364/oe.588571. 41845843

[309] Y. Li , B. Chen , X. Li , et al., “Parallel 4D Solid‐State LiDAR Enabled by an Integrated Si‐SiN Focal Plane Switch Array Chip,” Laser & Photonics Reviews 20, no. 12 (2026): e03167, 10.1002/lpor.202503167.

[310] E.‐S. Lee , J. Jin , Y.‐H. Sunwoo , et al., “Line‐Beam Scanning LiDARs Utilizing 128‐Channel Optical Phased Array on a Monolithically Integrated Polymer–SiN Platform,” Journal of Lightwave Technology (2026), 10.1109/JLT.2026.3674454.

[311] Z. Mingshi , W. Yubing , Z. Lanxuan , et al., “Phase‐Modulated Continuous‐Wave Coherent Ranging Method for Optical Phased Array Lidar,” Optics Express 31, no. 4 (2023): 6514–6528, 10.1364/oe.477952. 36823905

[312] M. Okano , C. Chong , and H. Hashemi , “Solid‐State Swept Source LiDAR: Single‐Sweep‐Per‐Frame 3D FMCW LiDAR With OPA‐Plus‐Dispersion Beam Steering,” Optics Express 34, no. 3 (2026): 4142–4157, 10.1364/oe.582856. 41715464

[313] J. Shan , H. Zhang , L. Li , W. Deng , and M. Sun , “Fast and Deterministic Optical Phased Array Calibration via Pointwise Optimisation,” Light: Advanced Manufacturing 4, no. 2 (2023): 115–123, 10.37188/lam.2023.010.

[314] W. Gao , W. Yao , J. Wang , et al., “High‐Precision Large‐Scale Optical Phased Array Calibration Using Physics‐Informed Neural Network With Transfer Learning,” Journal of Lightwave Technology 43, no. 22 (2025): 10203–10209, 10.1109/jlt.2025.3608167.

[315] J. Sved , S. Song , Y. Chen , L. Zhou , R. Minasian , and X. Yi , “Machine Learning Assisted Two‐Dimensional Beam‐Steering for Integrated Optical Phased Arrays,” Optics Communications 540 (2023): 129517, 10.1016/j.optcom.2023.129517.

[316] M. Mukherjee , “Wireless Communication‐Moving From RF to optical,” in 2016 3rd International Conference on Computing for Sustainable Global Development (INDIACom) (IEEE, 2016), 788–795.

[317] Z. Ghassemlooy , S. Zvanovec , M.‐A. Khalighi , W. O. Popoola , and J. Perez , “Optical Wireless Communication Systems,” Optik 151 (2017): 1–6: Optical Wireless Communication Systems, 10.1016/j.ijleo.2017.11.052.

[318] T. Koonen , J. Oh , K. Mekonnen , Z. Cao , and E. Tangdiongga , “Ultra‐High Capacity Indoor Optical Wireless Communication Using 2D‐Steered Pencil Beams,” Journal of Lightwave Technology 34, no. 20 (2016): 4802–4809, 10.1109/jlt.2016.2574855.

[319] C. W. Oh , Z. Cao , E. Tangdiongga , and T. Koonen , “Free‐Space Transmission With Passive 2D Beam Steering for Multi‐Gigabit‐Per‐Second Per‐Beam Indoor Optical Wireless Networks,” Optics Express 24, no. 17 (2016): 19211–19227, 10.1364/oe.24.019211. 27557201

[320] F. Feng , P. Sangwongngam , G. Faulkner , and D. O’Brien , “Wide Field‐of‐View Optical Broadcasting for Bi‐Directional Indoor Optical Wireless Communications Employing PAM‐4 Modulation,” Optics Letters 44, no. 24 (2019): 6009–6012, 10.1364/ol.44.006009. 32628206

[321] A. Gomez , K. Shi , C. Quintana , et al., “Beyond 100‐Gb/s Indoor Wide Field‐of‐View Optical Wireless Communications,” IEEE Photonics Technology Letters 27, no. 4 (2015): 367–370, 10.1109/lpt.2014.2374995.

[322] Ke Wang , A. Nirmalathas , C. Lim , K. Alameh , and S. Efstratios , “Full‐Duplex Gigabit Indoor Optical Wireless Communication System With CAP Modulation,” IEEE Photonics Technology Letters 28, no. 7 (2016): 790–793, 10.1109/lpt.2015.2514102.

[323] P. Brandl , S. Schidl , A. Polzer , W. Gaberl , and H. Zimmermann , “Optical Wireless Communication With Adaptive Focus and MEMS‐Based Beam Steering,” IEEE Photonics Technology Letters 25, no. 15 (2013): 1428–1431, 10.1109/lpt.2013.2266574.

[324] T. Koonen , “Indoor Optical Wireless Systems: Technology, Trends, and Applications,” Journal of Lightwave Technology 36, no. 8 (2018): 1459–1467, 10.1109/jlt.2017.2787614.

[325] W. Ren , P. Hou , Y. Jiang , X. Zeng , and J. Sun , “100 km One‐Point to Multi‐Point Simultaneous Laser Communication Based on 8 × 8 Matrix Vector Optical Phased Array,” Journal of Lightwave Technology 43, no. 17 (2025): 8029–8039, 10.1109/JLT.2025.3581583.

[326] Y. Kamiura , R. Doi , S. Hussein , M. Che , Y. Mikami , and K. Kato , “300‐GHz Beam‐Steering Wireless Communication Enabled by 4‐Array InGaAs UTC‐PD on SiC Substrate and Optical Phased Array,” Journal of Lightwave Technology (2026), 10.1109/JLT.2025.3553448.

[327] O. Balasiano , E. Wohlgemuth , I. Attia , et al., “Demonstration of Coherent Beam Combining for Atmospheric Free Space Optical Communication Over 10 km,” Journal of Lightwave Technology 42, no. 20 (2024): 7085–7094, 10.1109/jlt.2024.3419124.

[328] Z. Wang , X. Cai , P. Jiang , et al., “Enhancing Wireless Optical Communication Through Multi‐Beam Scanning and Bandwidth Optimization Using an Optical Phased Array,” Optics Communications 550 (2024): 129981, 10.1016/j.optcom.2023.129981.

[329] Y. Li , B. Chen , H. Du , et al., “Integrated Optical Wireless Communication Featured With Optical Phased Array Transceivers for Full‐Duplex and NonLine‐of‐Sight Transmission,” Laser & Photonics Reviews 20, no. 7 (2025): e00822, 10.1002/lpor.202500822.

[330] W. K. K. Kan , S. Guerber , S. Garcia , et al., “Two‐Dimensional Integrated Optical Phased Array With High Fill‐Factor Antennas,” Optics Express 33, no. 2 (2025): 3554–3561, 10.1364/oe.543258. 39876475

[331] D. Brinkley and S. Palese , and J.‐L. Plateau , “Optical Phased Arrays for Wireless Connectivity,” in ECOC 2024; 50th European Conference on Optical Communication (VDE, 2024), 99–102.

[332] J. Januszewicz , A. P. McWilliam , S. Dyer , et al., “Chip‐Scale Atomic Spectrometer With Silicon Nitride Optical Phased Array,” APL Photonics 10, no. 7 (2025): 076104, 10.1063/5.0273108.

[333] Z. Chen , M. Gagino , Y. Song , V. Dolores‐Calzadilla , E. Tangdiongga , and O. Raz , “InP Optical Phased Array for High‐Speed Wavefront Shaping Through Scattering Media,” Optics Letters 50, no. 12 (2025): 4122–4125, 10.1364/ol.563500. 40512957

[334] G. Hu , Y. Qin , H. Xu , and Ki T. Hon , “High‐Speed Multimode Fiber Imaging Using Binary‐Modulated Silicon Photonics Optical Phased Array,” Laser & Photonics Reviews 19, no. 13 (2025): 2402206, 10.1002/lpor.202402206.

[335] D. Lian , S. Zhao , Y. Ye , et al., “Tunable Optical Orbital Angular Momentum Beams Generation Using 2D Optical Phased Array With an Ultra‐Large Reconstructed Envelope,” Laser & Photonics Reviews 19, no. 24 (2025): e00212, 10.1002/lpor.202500212.

[336] S. Yang , B. Feng , G. Wu , F. Yu , J. Sha , and W. Jiang , “Arbitrary Precise Pattern Former Using Integrated Optical Phased Arrays for On‐Demand Beam Forming,” Laser & Photonics Reviews 20, no. 1 (2026): e00914, 10.1002/lpor.202500914.

[337] M. Chalupnik , A. Singh , J. Leatham , M. Lončar , and M. Soltani , “Nanophotonic Phased Array XY Hamiltonian Solver,” APL Photonics 9, no. 3 (2024): 031306, 10.1063/5.0187545.

[338] W. Yan , X. Song , Di Wu , et al., “Nonreciprocal Optical Routing via a Magneto‐Optical Phased Array on Silicon,” Photonics Research 13, no. 9 (2025): 2432–2441, 10.1364/prj.547240.

[339] E. Zhang and L. Zhang , “Optical Phased Array Receiver With Mode Diversity and Coherent Combination,” Nanophotonics 14, no. 7 (2025): 969–980, 10.1515/nanoph-2025-0038. PMC11980870 40212743

[340] F. Milojković , N. Verellen , R. Jansen , F. Peyskens , X. Rottenberg , and P. Van Dorpe , “Optical Phased Arrays for Wavefront Shaping in Forward Scattering Media,” Nanophotonics 14, no. 20 (2025): 3255–3264, 10.1515/nanoph-2025-0273. PMC12588563 41199930

[341] S. Hirata , N. Matsuda , K. Komatsu , T. Tanemura , and R. Horisaki , “Phase‐Bias‐Blind Computational Ghost Imaging With Optical Phased Array,” Optics Express 33, no. 10 (2025): 21132–21141, 10.1364/oe.563228. 40515021

[342] A. Derakhshandeh , S. Pachnicke , and P. A. Hoeher , “Underwater Wireless Laser‐Based Communications Using Optical Phased Array Antennas,” IEEE Photonics Journal 15, no. 5 (2023): 1–9, 10.1109/jphot.2023.3316729.

[343] H. Crawford‐Eng , A. Garcia Coleto , and J. Notaros , “Visible‐Spectrum‐Spanning Integrated Optical‐Phased‐Array‐Based Systems,” Optics Letters 50, no. 19 (2025): 6065–6068, 10.1364/ol.570107. 41032794

[344] S. Corsetti , M. Notaros , T. Sneh , A. Stafford , Z. A. Page , and J. Notaros , “Silicon‐Photonics‐Enabled Chip‐Based 3D Printer,” Light Science & Applications 13, no. 1 (2024): 132, 10.1038/s41377-024-01478-2. PMC11153580 38839804

[345] B. Narupai and A. Nelson , “100th Anniversary of Macromolecular Science Viewpoint: Macromolecular Materials for Additive Manufacturing,” ACS Macro Letters 9, no. 5 (2020): 627–638, 10.1021/acsmacrolett.0c00200. 35648567

[346] T. J. Wallin , J. Pikul , and R. F. Shepherd , “3D Printing of Soft Robotic Systems,” Nature Reviews Materials 3, no. 6 (2018): 84–100, 10.1038/s41578-018-0002-2.

[347] S. Clark Ligon , R. Liska , J. Stampfl , M. Gurr , and R. Muelhaupt , “Polymers for 3D Printing and Customized Additive Manufacturing,” Chemical Reviews 117, no. 15 (2017): 10212–10290, 10.1021/acs.chemrev.7b00074. PMC5553103 28756658

[348] R. L. Truby and J. A. Lewis , “Printing Soft Matter in Three Dimensions,” Nature 540, no. 7633 (2016): 371–378, 10.1038/nature21003. 27974748

[349] M.‐C. Zhong , W. Xun‐Bin , J.‐H. Zhou , Zi‐Q. Wang , and Li Yin‐Mei , “Trapping Red Blood Cells in Living Animals Using Optical Tweezers,” Nature Communications 4 (2013), 10.1038/ncomms2786. 23612309

[350] M. Raval , A. Yaacobi , D. Coleman , et al., “Nanophotonic Phased Array for Visible Light Image Projection,” in 2016 IEEE PHOTONICS CONFERENCE (IPC), IEEE Photonics Conference (IEEE, 2016), 206–207.

[351] J. Notaros , M. Raval , M. Notaros , and M. R. Watts , “Integrated‐Phased‐Array‐Based Visible‐Light Near‐Eye Holographic Projector,” in 2019 CONFERENCE ON LASERS AND ELECTRO‐OPTICS (CLEO), Conference on Lasers and Electro‐Optics, IEEE; AdValue Photon; Amer Elements; Class5 Photon; Coherent; GoFoton; Light Convers; LightTrans; MKS; OZ Opt Online; Santec; ThorLabs; UQDevices; YSL Photon. Conference on Lasers and Electro‐Optics (CLEO), 2019): San Jose, CA, MAY 05‐10, 2019.

[352] Z. Wang , L. Liu , P. Jiang , et al., “Innovative OPA‐Based Optical Chip for Enhanced Digital Holography,” Optics Express 31, no. 26 (2023): 44028, 10.1364/oe.507097. 38178484

[353] J. Bütow , J. S. Eismann , V. Sharma , D. Brandmüller , and P. Banzer , “Generating Free‐Space Structured Light With Programmable Integrated Photonics,” Nature Photonics 18, no. 3 (2024): 243–249, 10.1038/s41566-023-01354-2.

[354] Y. Wen , F. Yang , J. Song , and Z. Han , “Joint Design of Optical Phased Array Beamforming and OFDM Waveform in Optical Wireless Integrated Sensing and Communication,” IEEE Transactions on Wireless Communications 25 (2026): 9703–9718, 10.1109/twc.2025.3647104.

[355] J. Guo , W. Zhang , Yu Yu , and X. Zhang , “Modular Multi‐Aperture Optical Phased Array System Enabling Solid‐State Full‐Space Beam Steering,” Journal of Lightwave Technology 43, no. 22 (2025): 10058–10064, 10.1109/jlt.2025.3610878.

